# Increasing membrane cholesterol of neurons in culture recapitulates Alzheimer’s disease early phenotypes

**DOI:** 10.1186/1750-1326-9-60

**Published:** 2014-12-18

**Authors:** Catherine Marquer, Jeanne Laine, Luce Dauphinot, Linda Hanbouch, Camille Lemercier-Neuillet, Nathalie Pierrot, Koen Bossers, Mickael Le, Fabian Corlier, Caroline Benstaali, Frédéric Saudou, Gopal Thinakaran, Nathalie Cartier, Jean-Noël Octave, Charles Duyckaerts, Marie-Claude Potier

**Affiliations:** Sorbonne Universités, UPMC Univ Paris 06, Inserm, CNRS, UM 75, U 1127, UMR 7225, ICM 75013 Paris, France; Université catholique de Louvain, Institute of Neuroscience, Brussels, 1200 Belgium; Neuroregeneration Group, Netherlands Institute for Neuroscience, an Institute of the Royal Netherlands Academy of Arts and Sciences, Amsterdam, 1105 BA The Netherlands; Institut Curie, CNRS UMR, Orsay, 91405 France; Departments of Neurobiology, Neurology, and Pathology, The University of Chicago, Chicago, IL 60637 USA; INSERM U986 94276 Le Kremlin-Bicêtre, and University Paris-Sud, 91400 Orsay, France

**Keywords:** Alzheimer’s disease, Amyloid precursor protein, Cholesterol, Endosomes, Neurons, Axonal transport

## Abstract

**Background:**

It is suspected that excess of brain cholesterol plays a role in Alzheimer’s disease (AD). Membrane-associated cholesterol was shown to be increased in the brain of individuals with sporadic AD and to correlate with the severity of the disease. We hypothesized that an increase of membrane cholesterol could trigger sporadic AD early phenotypes.

**Results:**

We thus acutely loaded the plasma membrane of cultured neurons with cholesterol to reach the 30% increase observed in AD brains. We found changes in gene expression profiles that are reminiscent of early AD stages. We also observed early AD cellular phenotypes. Indeed we found enlarged and aggregated early endosomes using confocal and electron microscopy after immunocytochemistry. In addition amyloid precursor protein vesicular transport was inhibited in neuronal processes, as seen by live-imaging. Finally transient membrane cholesterol loading lead to significantly increased amyloid-β42 secretion.

**Conclusions:**

Membrane cholesterol increase in cultured neurons reproduces most early AD changes and could thus be a relevant model for deciphering AD mechanisms and identifying new therapeutic targets.

**Electronic supplementary material:**

The online version of this article (doi:10.1186/1750-1326-9-60) contains supplementary material, which is available to authorized users.

## Background

Neuropathological hallmarks of Alzheimer’s disease (AD) are extracellular senile plaques, composed primarily of amyloid peptides (Aβ), and intracellular neurofibrillary tangles of hyperphosphorylated tau protein [1]. Aβ is derived from sequential proteolytic processing of its membrane precursor Amyloid Precursor Protein (APP) by the β- and γ-secretases [2]. Although transgenic mouse models expressing the mutations of APP and components of the γ-secretase found in familial AD reproduce amyloid pathology, there are currently no animal models mimicking sporadic AD [3].

Cholesterol is increasingly linked to AD pathology [4]. It is increased in AD brains [5, 6] and we found by Time of flight –Secondary Ion Mass Spectrometry (Tof-SIMS), that a 30% cholesterol increase could be observed in AD brain samples most likely in various cell types [7]. Moreover, increase in membrane-associated free cholesterol were correlated with the severity of the disease as opposed to intracellular cholesterol, cholesterol from the extracellular space or from the senile plaques [5]. Additionally, the ϵ4 allele of the *APOE* gene encoding apolipoprotein E, the transporter of cholesterol in the brain, is the most important risk factor of AD [8]. APP as well as β and γ-secretases are residents of cholesterol-enriched membrane microdomains termed lipid rafts [9, 10]. Levels of cholesterol control the partition of APP and its secretases in lipid rafts [11, 12] as well as APP internalization and Aβ production [11, 13]. Moreover, a binding site for cholesterol in APP has been described [14].

Despite this substantial body of literature, it still remains unclear whether sporadic AD could be initiated by a disruption of cholesterol metabolism leading to a change in membrane cholesterol of neurons. To test this hypothesis, we triggered an acute increase of cholesterol at the membrane of neurons and assessed whether cellular changes similar to those detected early in the development of the disease could be observed. A 30% membrane cholesterol increase was produced to mimick what has been observed in AD brain samples. We did not use 3-hydroxy-3-methylglutaryl-coenzyme A (HMG-CoA) inhibitors such as statins, since they lead to non-specific effects via their action on isoprenoids and inflammation, or drugs such as U1866A that lead to accumulation of cholesterol in the lysosomal pathway [15–17]. We did not use uptake of LDL-cholesterol complex either since they can directly affect the endo-lysosomal pathway [18]. Instead we used methyl-beta-cyclodextrin/cholesterol complex (MβCD-cholesterol) which can deliver cholesterol directly at the plasma membrane of cultured neurons [12, 19]. After cholesterol increase we analyzed cellular phenotypes that correlate with sporadic AD progression. Gene expression changes were reported to correlate with AD pathology with a switch occurring at Braak stage III, when profound modifications in amyloid pathology take place [20]. Enlargement of the endosomal compartment was described as one of the earliest phenotypes of AD, present before the formation of plaques and absent in other neurodegenerative diseases [21]. Aβ was found to accumulate in these enlarged endosomes [22]. This phenotype occurs more frequently in individuals with the ϵ4 allele of *APOE,* suggesting a possible link with cholesterol metabolism [21]. Another phenotype associated with sporadic AD is axonal vesicular transport deficits, described in samples from individuals affected by the disease [23] and in AD mice models [24].

In this study, using our published experimental model for loading the plasma membrane of neurons with cholesterol to reach an increase of 30%, corresponding to levels detected in AD brain samples, we could recapitulate cellular phenotypes from early stages of the disease, suggesting a direct causal link between high cholesterol in the brain and cellular AD pathogenesis. We propose membrane cholesterol accumulation in cultured neurons to be a useful cellular model of AD.

## Results and discussion

### Gene expression changes after membrane cholesterol increase were similar to changes observed in early stages of AD

To assess gene expression modifications after loading the plasma membrane with 30% more cholesterol, we performed transcriptome analysis of embryonic rat cortical neurons treated or not with 1.4 mM MβCD-cholesterol. Typically the neuronal culture used contained less than 1% of non-neuronal cells (data not shown). RNAs were reverse-transcribed, labeled and hybridized to microarrays. Among the initial 44000 probes on the chip, 1540 genes were found to be significantly differentially expressed (t test, p < 0.05). These genes are listed in Additional file 1. Among them, 907 genes were down-regulated and 633 were up-regulated after membrane cholesterol loading.

To gain insight into the biological processes over-represented, we performed Gene Ontology (GO) category enrichment analysis (Additional file 2). The most significant enriched GO categories were the ones linked to the cholesterol pathway (GO: 003399: response to lipid, p = 3.36E-08; GO: 0010033: response to organic substance, p = 1.84E-08; GO: 0016126: sterol biosynthetic process, p = 7.86E-06). We thus examined genes involved in cholesterol biosynthesis (PW0000454 in the Rat Genome Database, http://rgd.mcw.edu/pathway/PW0000454/). Eleven of 18 genes from this pathway were significantly down-regulated after cholesterol increase (Table 1). Six genes (*Ebp, Fdps, Hmgcr, Lss, Nsdhl* and *Pvmk*) were not differentially expressed. We detected *Dhcr24* in control conditions only, suggesting that its expression is strongly down-regulated after cholesterol treatment. These results are in agreement with an adaptive compensation due to artificial cholesterol increase. We also observed that *Abca1* expression was significantly up-regulated, with a cholesterol to control ratio of 2.89 (p = 0.0449, t test). *Abca1* codes for ABCA1, a key player in cholesterol efflux from the cell [25].

**Table 1:** **Effects of cholesterol increase on expression levels of genes involved in the cholesterol biosynthetic pathway in cultured neurons**

Gene acronym	Gene full name	Cholesterol/control expression ratio	P value (t test)
***Acat2***	Acetyl-Coenzyme A acetyltransferase 2	0.32	0.00235
***Cyp51***	Cytochrome P450, family 51	0.48	0.00698
*Dhcr24*	24-dehydrocholesterol reductase	NA	NA
***Dhcr7***	7-dehydrocholesterol reductase	0.41	0.0278
*Ebp*	Emopamil binding protein (sterol isomerase)	0.72	0.06112
***Fdft1***	Farnesyl diphosphate farnesyl transferase 1	0.38	0.03888
*Fdps*	Farnesyl diphosphate synthase	0.42	0.11898
*Hmgcr*	3-hydroxy-3-methylglutaryl-CoA reductase	0.57	0.28355
***Hmgcs1***	3-hydroxy-3-methylglutaryl-CoA synthase 1 (soluble)	0.38	0.00355
***Idi1***	Isopentenyl-diphosphate delta isomerase 1	0.38	0.03937
***Lbr***	Lamin B receptor	0.64	0.03795
*Lss*	Lanosterol synthase (2,3-oxidosqualene-lanosterol cyclase)	0.56	0.13084
***Mvd***	Mevalonate (diphospho) decarboxylase	0.28	0.01896
***Mvk***	Mevalonate kinase	0.49	0.00199
*Nsdhl*	NAD(P) dependent steroid dehydrogenase-like	0.49	0.05092
*Pmvk*	Phosphomevalonate kinase	1.16	0.74918
***Sc5d***	Sterol-C5-desaturase	0.41	0.00863
***Sqle***	Squalene epoxidase	0.39	0.018

Genes in bold were found to be significantly differentially expressed (t test, p < 0.05).

**Figure 1:**
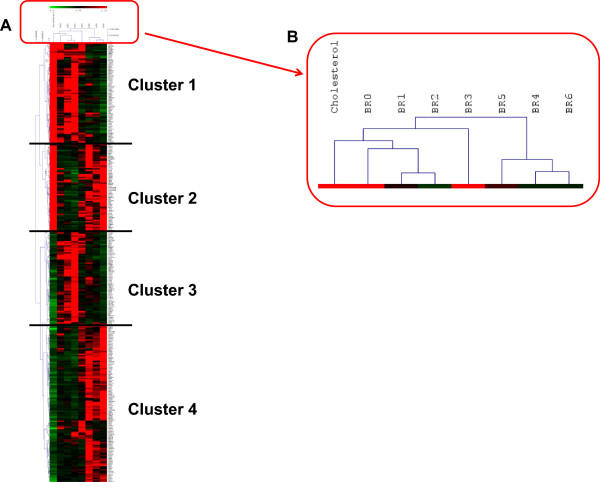
**Hierarchical Clustering of the 308 genes with significant expression changes after membrane cholesterol increase and during AD progression (A).** Magnification of the sample tree shows that early and late Braak stages are clearly discriminated while cholesterol treatment segregates with early stages **(B)**.

We then compared the list of genes differentially expressed after cholesterol increase with gene expression profiles of sporadic AD brain samples from different Braak stages [20]. Braak and Braak formalized the progression of neurofibrillary pathology in the cerebral cortex based on the topographical distribution of neurofibrillary tangles and neuropil threads [26]. Raw data from Bossers et al. [20] were normalized and analyzed by ANOVA. 4365 genes were found to have an interaction between expression level and Braak stage (p < 0.05). Among these, 308 were in common with our list of genes differentially expressed after membrane cholesterol loading in neurons (Additional file 3). To compare gene expression changes after cholesterol increase and between Braak stages, we performed a hierarchical clustering on the average expression levels of the 308 genes which were modulated in the two studies (Figure 1).

Based on the level of expression of these 308 genes, samples clustered according to their Braak stage. Namely, early Braak AD stages I–III, displaying both intraneuronal Aβ and amyloid plaques and late Braak AD stages IV–VI, displaying extracellular amyloid plaques only, belonged to distinct clusters (Figure 1). Samples corresponding to neurons treated with cholesterol segregated with early AD stages samples (Figure 1), suggesting that gene expression changes induced by membrane cholesterol loading are more similar to changes observed in early rather than late stages of AD. We identified four clusters of genes differentially expressed after cholesterol increase. In clusters 1 (71 genes) and 4 (112 genes), representing more than 59% of genes, expression was up- or down-regulated, respectively, as in early Braak stages. We analyzed the Gene Ontology annotation of these genes and found that most genes from cluster 1 were involved in synaptic activity and in mitochondrial function (Additional file 4). Cluster 1 also included genes involved in neuropeptide signaling pathway and in endocytosis (Additional file 4). Most genes from cluster 4 were involved in cell architecture, signaling and regulation of cell cycle (Additional file 5).

Thus a restricted number of genes modulated by cholesterol were sufficient to discriminate between early and late Braak stages, supporting the hypothesis that increasing membrane cholesterol induced changes that mirror early AD phenotypes. We further investigated whether cholesterol increase at the neuronal membrane could induce early AD molecular and cellular phenotypes.

### High membrane cholesterol increased early endosomes size and aggregation

Since endosome enlargement has been reported as an early phenotype of sporadic AD, we searched for genes involved in endocytosis that are differentially expressed after membrane cholesterol loading. One of the main regulator of endocytosis is phosphatidylinositol 4,5-bisphosphate (PIP2) [27]. PIP2 breakdown was shown to increase fission of endocytic vesicles from the plasma membrane [28]. Moreover, PIP2 was recently shown to be linked with cholesterol homeostasis [29]. Gene expression profiles suggested that cholesterol treatment increased PIP2 breakdown. Indeed the expression of two genes implicated in PIP2 breakdown, Plcb1and Pik3r1, was up-regulated after cholesterol treatment (Table 2). Microarrays results were confirmed by quantitative PCR experiments (Table 2). Plcb1 codes for 1-Phosphatidylinositol-4,5-bisphosphate phosphodiesterase beta-1 (PLCβ1) which catalyzes the formation of inositol 1,4,5-trisphosphate (IP3) and diacylglycerol (DAG) from PIP2 [27]. Pik3r1 codes for phosphatidylinositol 3-kinase (PI3K) regulatory subunit alpha (also called p85α). Class I PI3K phosphorylates PIP2, leading to the formation of phosphatidylinositol 3,4,5-trisphosphate (PIP3) [27]. Cholesterol loading could lead to increased PIP2 breakdown and thus to increased fission of endocytic vesicles from the membrane, resulting in higher endocytosis, and in the formation of enlarged endosomes as we have previously shown [13].

**Table 2:** **Effects of cholesterol increase on expression levels of genes involved in the endo-lysosomal pathway in cultured neurons**

Gene acronym	Microarrays		Quantitative PCR	
	Cholesterol/control expression ratio	P value (t test)	Cholesterol/control expression ratio	P value (t test)
***Plcb1***	1.45	0.0471	1.33	0.03239
***Pik3r1***	1.49	0.00279	1.31	0.01416
***Osbpl1a***	1.64	0.00524	1.52	0.02127
***Fyn***	1.58	0.02038	1.21	0.03649

**Figure 2:**
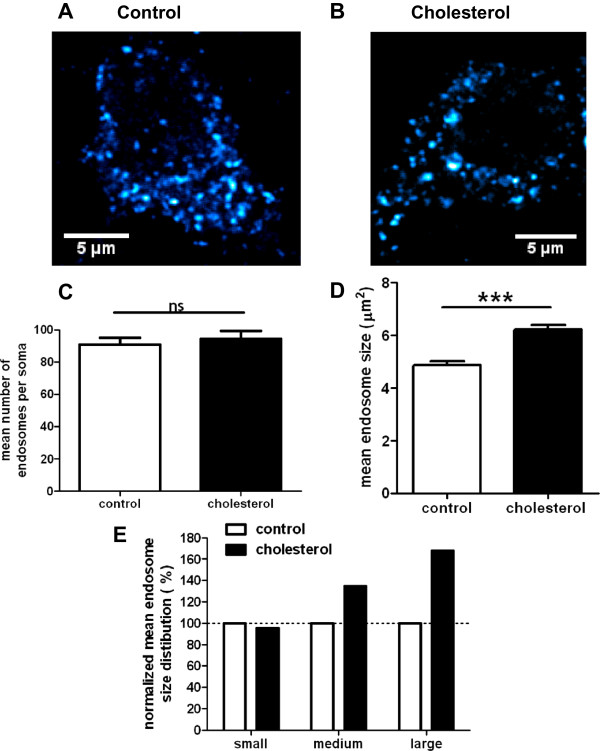
**Membrane cholesterol loading causes neuronal EEA1-positive early endosomes enlargement but does not impact on their number, as assessed by confocal microscopy. (A)** and **(B)** Representative confocal images of cortical neurons stained with an anti-EEA1 antibody in control conditions **(A)** or after cholesterol loading **(B)**. **(C)** Mean number of endosomes per soma (ns stands for p > 0.05 in unpaired Student’s t-test; n = 46 control neurons and 45 cholesterol-treated neurons observed in 3 independent experiments). **(D)** Mean endosomal size (*** stands for p < 0.001, Mann–Whitney test, 4171 control endosomes and 4248 cholesterol-treated endosomes, collected in 3 independent experiments). **(E)** Endosome size distribution of small-, medium- and large-sized endosomes, based on κ-means clustering and normalized to control conditions.

We then explored the impact of membrane cholesterol loading on the morphology of early endosomes. After treatment, neurons were fixed, stained with anti-EEA1 antibody and visualized by confocal microscopy (Figure 2A and B). The number and size of endosomes were quantified with the ICY software as described [30] (Figure 2C and D). The average number of endosomes per neuronal soma was similar in control conditions (91 ± 5 endosomes, n = 46 neurons in 3 independent experiments) and after treatment with MβCD-cholesterol (94 ± 5 endosomes, n = 45 neurons in 3 independent experiments) (p = 0.582, unpaired Student’s t test) (Figure 2C). The average size of early endosomes of treated neurons was 6.2 ± 0.2 μm^2^ (4248 endosomes, collected from 13–17 neurons in 3 independent experiments), as compared to 4.9 ± 0.1 μm^2^ (4172 endosomes, collected from 14–16 neurons in 3 independent experiments) for the control neurons, corresponding to an increase in size of 28% (p < 0.0001, Mann–Whitney test) (Figure 2D). We also analyzed the size distribution of the endosomes and used κ-means to obtain three clusters: small, medium and large (Figure 2E). In the control condition, the small, medium and large size classes represented 88.7, 10.4 and 0.9% of all endosomes, respectively. After cholesterol treatment, 84.5, 14.0 and 1.5% of endosomes were in the large, medium and large size classes, respectively. The distribution of endosome sizes was significantly shifted towards higher size after membrane cholesterol loading (p = 2.8 e-07, χ^2^). We confirmed this change in the morphology of early endosomes in synaptic-competent cortical neurons kept in culture for 14 days (DIV14) and treated similarly with cholesterol (Additional file 6).

**Figure 3:**
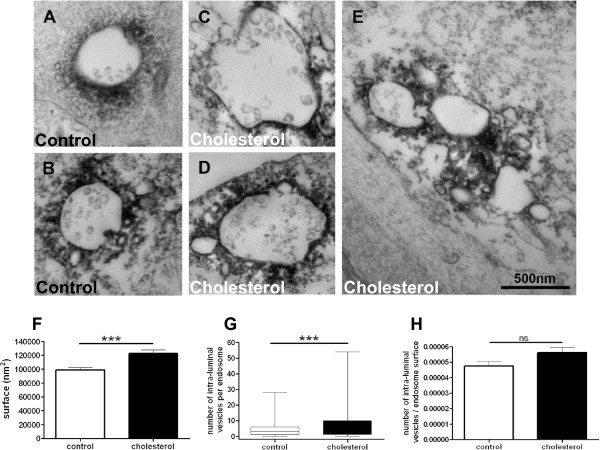
**Transmission electron microscopy reveals that membrane cholesterol increase leads to enlargement and aggregation of EEA1-positive early endosomes containing more intraluminal vesicles (ILVs). (A-E)** Representative electron micrographs of EEA1-labelled structures in control **(A and B)** or cholesterol-treated **(C, D and E)** cortical neurons. Bar in E = 500 nm and is for all micrographs. **(F)** Surface of individual EEA1-labelled endosomes (*** stands for p < 0.001 in unpaired t test with Welch’s correction, n = 244 control and 284 cholesterol-treated endosomes). **(G)** Number of ILVs per EEA1-labelled endosome (*** stands for p < 0.001 in unpaired t test with Welch’s correction, n = 1060 control and 1978 cholesterol-treated ILVs). **(H)** Number of ILVs to endosomal surface ratio (ns stands for p > 0.05 in unpaired Student’s t test, n = 244 control and 284 cholesterol-treated endosomes).

We used transmission electron microscopy (TEM) to examine enlarged endosomes in more detail. Cortical neurons were loaded with cholesterol, labeled using anti-EEA1 antibody, and prepared for TEM (Figure 3). EEA1 immunoperoxidase precipitates were found surrounding closely membrane-bound profiles of early endosomes as previously shown [31], containing variable numbers of intraluminal small vesicles (ILVs). Endosomes were often surrounded by small vesicles (see for instance Figure 3A) and also by some larger ones (Figure 3B-E). After cholesterol treatment, large vesicles surrounding early endosomes were more frequent (Figure 3C and D). The surface of individual EEA1-labelled profiles was quantified (Figure 3F). We observed that the surface of early endosomes in control cells (98635 ± 3457 nm^2^, 244 endosomes) was significantly smaller than it was in cholesterol-loaded neurons (122495 ± 4941 nm^2^, 284 endosomes) (24% increase, p < 0.0001, unpaired t test with Welch’s correction). After cholesterol loading, 17% of EEA1-positive endosomes formed aggregates (Figure 3E), whereas only 4% formed aggregates under control conditions. EEA1-positive endosomes thus appeared more prone to aggregation after membrane cholesterol increase. These results highlight that, after membrane cholesterol loading, endosomes were not only larger in surface but also formed more aggregates.

We investigated the number of ILVs per endosome (Figure 3G). In control conditions, EEA1 positive endosomes exhibited 4.3 ± 0.3 ILVs per endosome (1060 ILVs, 244 endosomes) whereas after cholesterol treatment, the ILVs number was significantly increased to 7.0 ± 0.5 ILVs per endosome (1978 ILVs, 284 endosomes) (p < 0.0001, unpaired t test with Welch’s correction). Thus, there were more ILVs in EEA1-positive endosomes after cholesterol treatment. Nevertheless, when we normalized the number of ILVs per endosome with the endosomal surface for each endosome, we found no significant difference between ratios in control conditions or after membrane cholesterol loading (Figure 3H, 244 control endosomes, 284 endosomes after cholesterol, p = 0.0535, unpaired Student’s t test), suggesting that the enrichment in ILVs may be linked to the endosome surface enlargement.

We thus showed that cholesterol increase at the neuronal membrane led to larger endosomal vesicles that were more prone to aggregation.

### High membrane cholesterol inhibited vesicular trafficking in neuritic processes

**Figure 4:**
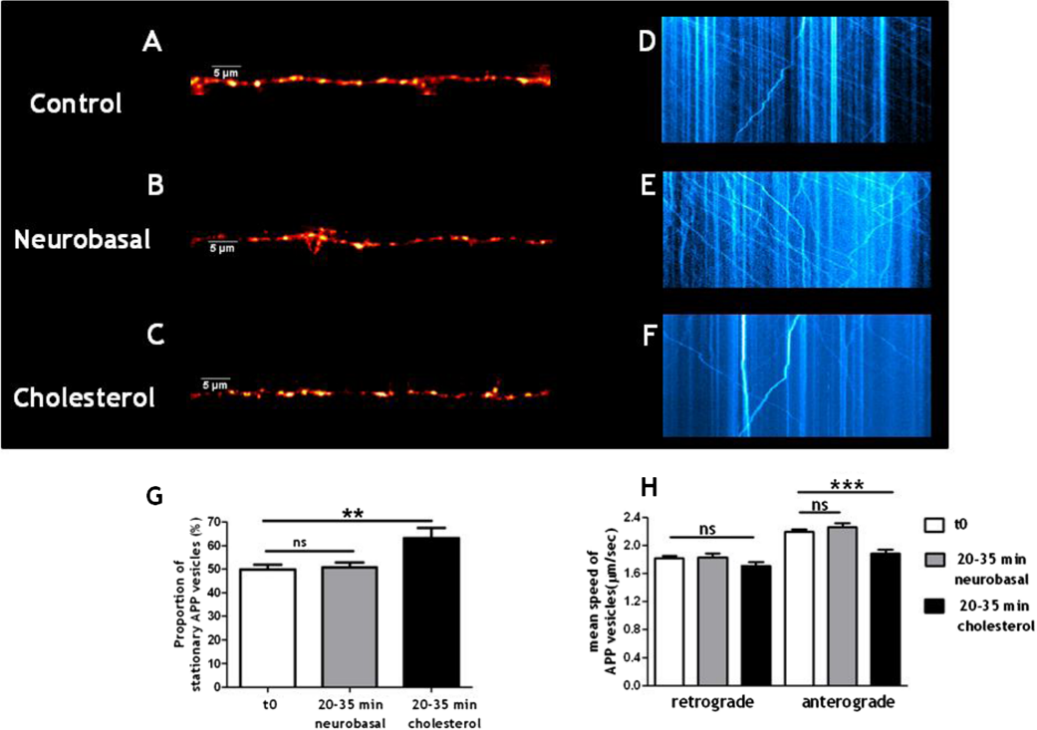
**Membrane cholesterol loading inhibits APP vesicular trafficking in neuronal processes. (A-F)** Representative APP-mCherry expression in neuritic processes and kymographs obtained from live-imaging experiments in control condition **(A and D)**, after neurobasal medium alone addition **(B and E)** or after MβCD-cholesterol dissolved in neurobasal medium treatment **(C and F)**. **(G)** Proportion of stationary APP vesicles (ns stands for p > 0.05 in unpaired Student’s t test, n = 52 control and 24 neurobasal-treated neurons and ** for p < 0.01 in unpaired t test with Welch’s correction, n = 52 control and 30 cholesterol-treated neurons). **(H)** Mean velocity of the remaining moving vesicles. For retrograde transport, ns stands for p > 0.05 in one-way ANOVA with Tukey’s post-test (n = 595 measures for control, 218 measures for neurobasal-treated and 268 measures for cholesterol-treated neurons). For anterograde transport, ns stands for p > 0.05 and *** for p < 0.001 in one-way ANOVA with Tukey’s post-test (n = 726 measures for control, 365 measures for neurobasal-treated and 241 measures for cholesterol-treated neurons).

Defective axonal vesicular transport is associated with early stages of sporadic AD [23, 24, 32]. We thus addressed the effect of membrane cholesterol loading on APP vesicular transport in neuronal processes by performing live imaging experiments on neurons expressing APP-mCherry. Typical APP-mCherry expression in processes is presented in Figure 4 under control condition (A), after addition of neurobasal medium alone (B) or of MβCD-cholesterol dissolved in neurobasal medium (C).

As previously reported, APP exhibited both anterograde (away from the soma) and retrograde transport (towards the soma) [33]. The mean speed of APP vesicles was 2.2 ± 0.03 μm/sec (n = 726 measures from 46 neurons in three independent cultures) for anterograde transport and 1.8 ± 0.03 μm/sec (n = 595 measures from 46 neurons in three independent cultures) for retrograde transport in control conditions. A wide range of values have been reported for APP velocities. Discrepancies could be due to different APP constructs, culture conditions, maturity of the neurons and also to the methods of acquisition and analysis of the data. The mean velocity we obtained for anterograde transport (2.2 μm/sec) fits in the reported range (0.8 to 4.5 μm/sec) [33–38].

The mobility of APP-containing vesicles was determined at time 0 and then kinetically followed over half an hour after neurobasal medium alone or MBCD-cholesterol dissolved in neurobasal medium addition. For each time point, a kymograph was drawn (Figure 4D-F) and the percentage of immobile vesicles (i.e., speed < 0.5 μm/sec in either direction) was extracted (Figure 4G). At time zero, 50 ± 2% of APP vesicles were stationary (n = 52 neurons observed in 13 independent experiments from 4 different cultures). Adding fresh neurobasal medium did not modify the mobility of APP vesicles (51 ± 2%, n = 24 neurons in 6 independent kinetic experiments from three different cultures; p = 0.802, unpaired Student’s t test). However, loading plasma membrane with cholesterol significantly increased the percentage of stationary vesicles (63 ± 4%, n = 30 neurons in 6 independent kinetic experiments from three different cultures; p = 0.008, unpaired t test with Welch’s correction).

We analyzed the velocity of the remaining moving vesicles. The mean speed of retrograde transport of APP vesicles was 1.8 ± 0.05 μm/sec (n = 218 measures from 21 neurons in three independent cultures) after neurobasal medium addition and 1.7 ± 0.05 μm/sec (n = 268 measures from 29 neurons in three independent cultures) after membrane loading with cholesterol (Figure 4H). This retrograde velocity was not modified by neurobasal medium nor by cholesterol (p = 0.1658, one-way ANOVA). The mean speed of anterograde transport of APP vesicles was 2.3 ± 0.05 μm/sec (n = 365 measures from 21 neurons in three independent cultures) after neurobasal medium addition and 1.9 ± 0.06 μm/sec (n = 241 measures from 29 neurons in three independent cultures) after membrane loading with cholesterol (Figure 4H). Mean anterograde transport speeds were significantly different (p < 0.0001, one-way ANOVA). Performing Tukey’s post-test revealed that there was no significant difference between mean anterograde speeds of APP vesicles at time 0 or after neurobasal addition (p > 0.05) while anterograde speeds after membrane loading with cholesterol were significantly decreased from anterograde speeds at time 0 (p < 0.001) or after neurobasal addition (p < 0.001).

We found that cholesterol increase led to decreased mobility of APP-containing vesicles while the speed of the remaining vesicles in movement was not affected in the retrograde direction but significantly decreased in the anterograde direction.

Cholesterol has been suggested to reduce the mobility of vesicles transported along microtubules [39–41] though this effect was not described previously in neurons, to our knowledge. In non-neuronal cell types, cholesterol levels can be sensed by oxysterol-binding protein ORP1L, thus impacting on the rab7/RILP/p150Glued complex and on the dynein/dynactin complex which is essential for retrograde transport [40]. Dynein dysfunction actually disrupts both retrograde and anterograde transport [42], through an increase in rab3 GTPase [42]. Interestingly, rab3 is essential for APP vesicular transport in neuronal processes [43]. We observed that expression of *Osbpl1a,* which encodes ORP1L, was up-regulated after membrane cholesterol loading (Table 2).

### High membrane cholesterol increased endogenous Aβ 42 secretion in cultured neurons

**Figure 5:**
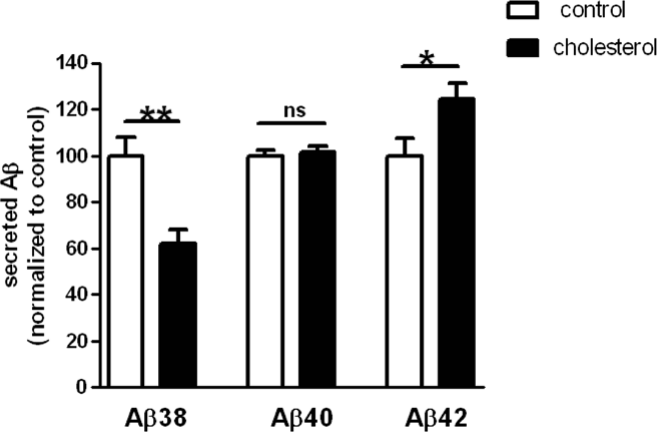
**Membrane cholesterol increase in cultured cortical neurons leads to lower Aβ38 (38% less, p = 0.0017, unpaired Student’s t-test, n = 12 control and 10 cholesterol assays in three independent experiments) and higher Aβ42 secretion (24% more, p = 0.0334, unpaired Student’s t-test, n = 11 control and 10 cholesterol assays in three independent experiments), while Aβ40 stayed unchanged (less than 2% change, p = 0.6322, unpaired Student’s t-test, n = 16 control and 15 cholesterol assays in four independent experiments), as assessed by MesoScale Discovery dosage.** ns stands for non-significant (p > 0.05), *for p < 0.05 and **for p < 0.01 in unpaired Student’s t-test.

We previously showed in cultured cell lines that increase of cholesterol at the plasma membrane can trigger APP endocytosis and Aβ secretion [13]. Here we quantified the levels of endogenously secreted Aβ peptides after loading the plasma membrane of embryonic rat cortical neurons with 30% more cholesterol. Concentrations of Aβ peptides of 38, 40 and 42 amino acids were assessed using Mesoscale Discovery multiplex assay. Conditioned media was collected after 24 hours in order to reach the sensitivity threshold for less-abundant isoforms quantification. Overall, the amount of Aβ peptides was comparable between control and cholesterol-loaded neurons (p = 0.8397, t test). Nevertheless, we observed discrepancies when we analyzed each isoform separately. While the levels of Aβ40 remained unchanged after cholesterol treatment (less than 2% change), Aβ38 exhibited a significant decrease (38% less) and Aβ42 a significant increase (24% more) (Figure 5).

Increased membrane cholesterol was thus associated with a higher production of endogenous neuronal Aβ42 peptides and with an elevated Aβ42 to Aβ40 ratio. Increase of Aβ42 secretion could be highly detrimental to neurons as this isoform is the most prone to form toxic oligomeric species [44]. We previously showed that, in HEK293 cells over-expressing the Swedish mutant of APP, both Aβ40 and Aβ42 peptides were up-regulated after cholesterol augmentation [13], thus highlighting the importance of both cell type and APP isoform when studying APP processing.

It was recently reported that Aβ38 results from γ-secretase processing of either Aβ42 or Aβ43 [45]. Interestingly, APP lysine 624, which was described as essential in regulating the final Aβ peptide length released by γ-secretase [46], is located in the APP/cholesterol interaction site [14]. Excess of cholesterol binding to APP may thus alter γ-secretase processing and impair the switch from Aβ42 to Aβ38, leading to increased Aβ42 and decreased Aβ38 peptides levels we observed.

## Conclusion

Membrane cholesterol has been shown to be increased in post-mortem brains from sporadic AD patients and to correlate with disease progression [5]. We suggested that an increase of membrane cholesterol could be an early event in the etiology of sporadic AD. To test this hypothesis, we acutely increased levels of plasma membrane cholesterol of neurons in culture. This treatment induced a 30% increase mimicking what was observed in AD brains [5–7]. We showed that this transient membrane cholesterol increase triggered Aβ42 over-production, endosomal enlargement, vesicular transport deficits in neuronal processes and gene expression modulation as in early sporadic AD. This model of neuronal primary cultures treated with cholesterol could thus be relevant to study early events in sporadic AD.

Using both confocal and electron microscopy, we investigated the number, surface, ILVs number and aggregation status of EEA1-positive early endosomes after cholesterol treatment. This is to our knowledge the first time that neuronal endosomal enlargement is investigated in such quantitative detail. After membrane cholesterol loading, the number of early endosomes per neuron was not altered but we found that their size was enlarged and that more ILVs were associated with each endosome. EEA1-positive endosomes in neurons loaded with cholesterol were also more prone to form aggregates. As cholesterol is highly concentrated in myelin [47], excess cholesterol in sporadic AD brains could result from demyelination linked to age, the major risk factor for AD. Indeed, genes involved in cholesterol synthesis were down-regulated in a mouse demyelination model [48], as we also observed in our model of external cholesterol addition. It still remains unclear whether myelin degradation could be the origin of higher membrane cholesterol that is described as a marker of disease progression [5]. The topography of cholesterol embedded in the membrane seems to be crucial as was highlighted in an AD mouse model where the cholesterol content of lipid rafts, but not the total brain cholesterol levels, was correlated with amyloid load and behavioral deficits [49]. The link between tau pathology, the other hallmark of AD, and cholesterol is still unclear. Here we show that membrane cholesterol increase leads to overexpression of the kinase Fyn gene using microarray gene profiling and RT-QPCR (Table 2). Tau is known to interact with Fyn and to facilitate Fyn targeting to dendrites. Once in the postsynaptic compartment, Fyn phosphorylates the *N*-methyl-D-aspartate (NMDA) receptor subunit 2B (NR2B) and stabilizes its interaction with postsynaptic density protein 95 (PSD95) (reviewed in [50]. The NR2B/PSD95 interaction is essential in mediating Aβ-induced excitotoxicity [51]. Fyn could thus be an interesting link between cholesterol, Aβ and tau.

In conclusion we have shown that an increase in neuronal membrane cholesterol triggers APP processing, endosomal trafficking and axonal transport abnormalities and induces gene expression changes that are reminiscent of early stages of sporadic AD. We propose that an increase in membrane cholesterol linked with age is one of the initial events that could trigger sporadic AD. Thus specifically decreasing neuronal membrane cholesterol could be an interesting therapeutic strategy, which has already been successfully applied in mouse models of AD [49, 52]. It was recently shown that stimulating cholesterol synthesis in mouse models overexpressing amyloid induces tau phosphorylation and neurofibrillary tangles formation, suggesting that cholesterol increase might trigger amyloid and tau pathologies [53]. Loading the membrane of cultured neurons with cholesterol could thus be used as a new cellular model to study early AD changes, identify new targets and screen new molecules.

## Methods

### Plasmids and reagents

The APP_751_ plasmid was a kind gift from Dr. Frederic Checler (IPMC, Valbonne, France). The APP-mCherry plasmid was generated by introducing the APP_751_ sequence in the pmCherry-N1 vector (Clontech, Mountain View, CA, USA) at the XmaI/AgeI restriction site. MβCD-cholesterol complex, saponin, bovine serum albumin (BSA), sucrose and poly-L-lysine were purchased from Sigma-Aldrich (Saint-Louis, MO, USA). The antibody directed against EEA1 (Early Endosome Antigen 1) was from Cell Signaling Technology (Danvers, MA, USA). Goat anti rabbit IgG coupled to Alexa568 was from Life Technologies (Carlsbad, CA, USA).

### Primary neuronal cultures

Primary hippocampal and cortical cultures were prepared from E16-18 OFA Sprague Dawley rat embryos (Charles River, Wilmington, MA, USA). Hippocampi and cortices were dissected in cold PBS supplemented with 45% glucose (Sigma-Aldrich, Saint-Louis, MO, USA). Digestion was performed in a 0,05% solution of Trypsin-EDTA (Life Technologies, Carlsbad, CA, USA) for 25 min at 37°C. Tissues were then mechanically dissociated in Dulbecco’s Modified Eagle Medium-1 (Life Technologies, Carlsbad, CA, USA) supplemented with 5% fetal bovine serum (Life Technologies, Carlsbad, CA, USA) and centrifuged for 10 min at 800 rpm. Neurons were resuspended in neurobasal medium supplemented with 2% B27, 2 mM glutamax, 1% penicillin/streptomycin (all from Life Technologies, Carlsbad, CA, USA) and counted.

### Cholesterol modulation

Unless otherwise mentioned, neurons were washed twice with neurobasal medium, treated with 1.4 mM MβCD-cholesterol dissolved in neurobasal medium and then washed three times with neurobasal medium. Treatment of neurons with 1.4 mM MβCD-cholesterol for 30 min resulted in an increase of 28.4 ± 6.0% of cellular cholesterol levels, as assessed previously by filipin staining [12].

### Aβ 38, 40 and 42 measurements

Cortical neurons were plated on 12-well plates coated with poly-L-lysine (1 mg/ml) at a density of 2 million neurons per well and maintained at 37°C in a humidified 5% CO_2_ atmosphere. After cholesterol treatment at DIV4-6, neurons were placed in fresh neurobasal medium supplemented with 2% B27, 2 mM glutamax for 24 h. Supernatants were collected on ice in polypropylene tubes (Corning, Corning, NY, USA) containing a protease inhibitor cocktail (Roche, Penzberg, Germany) and were then stored at -80°C. Concentrations of the Aβ38, Aβ40 and Aβ42 species of β-amyloid peptide were measured by multiplex Electro-Chemiluminescence Immuno-Assay (ECLIA). Assays were performed according to the manufacturer's instructions. Briefly, samples were analyzed using Meso Scale Discovery (MSD) SECTOR™ Imager 2400 (Meso Scale Discovery, Gaithersburg, MD, USA), with the Rodent Aβ triplex kit (also from MSD); carbon 96-well plates contained in each well four capture spots, one of which was blocked with BSA (as standard curve control), and the three others coated with isoform specific anti-Aβ antibodies specific for Aβ38, Aβ40, Aβ42, respectively. 100 μl of blocking buffer solution were added to all wells to avoid non-specific binding. The plates were then sealed, wrapped in tin foil, and incubated at room temperature on a plate shaker (600 rpm) for 1 h. Wells were then washed three times with washing buffer, and 25 μl of the standards (Aβ38, Aβ40, Aβ42) and samples were then added to the wells, followed by an Aβ-detecting antibody at 1 μg/ml (MSD) labelled with a Ruthenium (II) trisbipyridine N-hydroxysuccinimide ester; this detection antibody was 4G8 (which recognizes the epitope Aβ18-22 of the human and rodent peptide). Plates were then aspirated and washed 3 times. MSD read buffer (containing TPA) was added to wells before reading on the Sector Imager. A small electric current passed through a micro-electrode present in each well producing a redox reaction of the Ru^2+^ cation, emitting 620 nm red light. The concentration of each Aβ isoform was calculated for each sample, using dose–response curves, the blank being *cell-less* culture medium.

### EEA1 Immunocytochemistry and confocal microscopy

Cells cultured on poly-L-lysine-coated coverslips were fixed at DIV4-5 or DIV14 using a solution of 4% paraformaldehyde and 4% sucrose in Phosphate Buffered Saline (PBS) for 20 min at room temperature. Cells were then washed twice in PBS and incubated with NH4Cl (50 mM in PBS) for 10 min. Cells were washed twice again in PBS. They were permeabilized with solution A (0.3% BSA and 0.05% saponin in PBS) for 45 min at 37°C and were then incubated at room temperature for 1 h with primary antibody against EEA1 diluted (1/1000) in solution A. Cells were subsequently incubated at room temperature for 1 h with goat anti rabbit secondary antibody conjugated to Alexa568 diluted in solution A (1/1000). Coverslips were mounted in Fluoromount-G (SouthernBiotech, Birmingham, AL, USA). Z-stacks of neurons were acquired on a Fluoview FV1000 confocal microscope (Olympus, Tokyo, Japan) with the 543 nm line of a He/Ne laser. Fluorescence was collected with a 60× plan apochromat immersion oil objective (NA 1.35) between 560–660 nm. The mean endosome size and the mean endosome number per neuron were analyzed with ICY software [54]. Between 13 and 17 neurons were analyzed for each experiment. Each experiment was independently repeated 3 times.

### Pre-embedding immunoperoxidase electron microscopy

Neurons grown on Thermanox coverslips were processed through all stages *in situ*. They were fixed with 4% PFA, 0.1% glutaraldehyde diluted in PBS, rinsed with PBS, cryoprotected in 30% glycerol, 30% ethylene glycol in PBS and stored at -20°. After PBS rinses, they were blocked in 5% normal goat serum and incubated at room temperature overnight with the antibody against EEA1 diluted 1/1000 in PBS. A biotinylated anti-rabbit IgG (Vector, CA, USA) was applied as secondary antibody (1/200 in PBS, 2 h), followed by ABC peroxidase complex (Vectastain Elite, Vector, CA, USA) with 0.05% diaminobenzidine as chromogen. After 2% OsO_4_ post-fixation and dehydration in graded acetone including a 1% uranyl staining step in 70% acetone, coverslips were embedded in Epon resin. Thin (70 nm) sections were lightly stained with lead citrate and observed under a Philips CM120 electron microscope (Philips, Eindhoven, The Netherlands) operated at 80 kV. Images were recorded with a Morada digital camera (Olympus Soft Imaging Solutions GmbH, Münster, Germany). The measurements were performed with the associated iTEM software.

### Videomicroscopy

Cortical neurons in suspension (5 million) were electroporated with Amaxa Nucleofector kit for rat neurons (Lonza, Basel, Switzerland) according to the supplier’s manual. Electroporated neurons were plated on 8-well Labteks coated with poly-L-lysine (1 mg/ml). After 3 hours, medium was replaced with fresh neurobasal supplemented with 2% B27, 2 mM glutamax and 1% penicillin/streptomycin. Neurons were maintained at 37°C in a humidified 5% CO_2_ atmosphere. Sixteen hours before performing video experiments, 10 μM forskolin (Sigma-Aldrich, Saint-Louis, MO, USA) and 100 μM IBMX (Sigma-Aldrich, Saint-Louis, MO, USA) were added to the medium. Videomicroscopy experiments were performed at DIV3-5. APP-containing vesicles were imaged in control condition and then for 35 minutes after adding neurobasal medium alone or MβCD-cholesterol dissolved in neurobasal medium (1.4 mM final concentration). Live videomicroscopy was carried out using an Axiovert 200 microscope (Zeiss, Jena, Germany) with a PL APO oil × 63 objective of numerical aperture of 1.40. Records were made with a Photometrics Evolve 512 camera (Roper Scientific, Trenton, NJ, USA) controlled by Metamorph software (Molecular Devices, Sunnyvale, CA, USA). Stacks were acquired at 37°C. Images were collected in stream set at 1 × 1 binning with an exposure time of 200 ms. Kymographs were generated and analyzed with a homemade ImageJ plugin, KymoToolbox, available upon request (contact: Fabrice.Cordelieres@curie.fr). Segmental velocities were defined as the speed a particle travels in one direction without a pause or a reversal in the direction of movement.

### cRNA probe preparation and hybridization

Hippocampal rat neurons (1.1 to 1.4 million) were treated with MβCD-cholesterol at DIV 4–6 and left in neurobasal medium for 4 h and 30 min. They were then harvested and centrifuged. The dry pellet was kept at -80°C. Total RNAs were extracted using Nucleospin RNA II kit (Macherey Nagel, Duren, Germany) in accordance with the manufacturer’s protocol. The quality and quantity of each RNA preparation were assessed on an Agilent 2100 Bioanalyzer with RNA 6000 NanoChips (Agilent Technologies, Santa Clara, CA, USA). One hundred ng of each RNA were amplified and labeled with Cy3 using the Low Imput Quick Amp labeling kit (Agilent Technologies, Santa Clara, CA, USA) according to the manufacturer’s instructions. After purification and quantification on a Nanodrop (ThermoFisher Scientific, Waltham, MA, USA), 2 μg of each Cy3-cRNA were hybridized overnight on Whole Rat Genome Microarray 4×44 K (Agilent Technologies, Santa Clara, CA, USA) according to the manufacturer’s instructions.

### Microarray data analysis

Microarray data were acquired on a ScanArray GX (Perkin Elmer, Waltham, MA, USA) with a resolution of 5 μm and analyzed with Mapix 5.0.0 software (Innopsys, Carbonne, France). For each sample, raw data consisted of the Median Feature Intensity – Median Background Feature (F-B) at 532 nm wavelength. These raw data were log2-transformed and quantile-normalized under the R freeware (http://www.r-project.org). The statistical analysis was performed on these normalized data under the R freeware. All the microarray data have been deposited on the GEO database under the accession number GSE46221.

Gene Ontology (GO) category enrichment analysis was realized using the web-based GOrilla application [55, 56] (http://cbl-gorilla.cs.technion.ac.il/). Statistical significance was set to a p value <0.001.

Hierarchical Classification was performed using MeV 4.7.4 software (http://www.tm4.org/).

### Quantitative PCR (qPCR)

Three hundred nanograms of each RNA were individually reverse-transcribed into cDNAs for 2 hours at 42°C using the Maxima First strand synthesis kit (Fermentas GmbH, Germany) according to the manufacturer’s instructions. qPCR assays were performed in a Lightcycler® 480 System (Roche), in the presence of 200nM of each primer, 100nM of specific hydrolysis probe (designed with Universal Probe Library, Roche Applied Science) and 1X Solaris qPCR Master mix (Fermentas GmbH, Germany). Gene expression was normalized using HPRT and pPib as reference genes.

### Statistical analysis

Most statistical calculations were performed using GraphPad Prism software (version 5.0). All the data are given as mean ± SEM.

κ-means analysis of fluorescently-labelled endosome size was performed using the statistic analysis toolbox of Matlab software (version R2012a). Endosome sizes under control conditions were distributed into three size classes defined by the κ-means algorithm using the squared Euclidian distances. This iterative partitioning minimizes the sum, over all clusters, of the within-cluster sums of point-to-cluster-centroid distances. Each endosome was assigned an index corresponding to one of the three clusters defined in the iterative loop (small, medium, large). The partition of endosome sizes after cholesterol treatment in these three classes was then determined.

The χ^2^ comparison test of the two distributions of endosome size was made with Matlab sofware (version R2012a) using the following formula:


with i (1 to 3) as the size categories (small, medium, large) and j (1 to 2) as the two groups (control and treated). n is the number of neurons falling into each class. The degree of freedom was (n_i_-1) × (n_j_-1) = 2.

## Electronic supplementary material

Additional file 1: **Significantly differentially expressed genes after cholesterol treatment of cultured hippocampal neurons (t test, p < 0.05).** (DOCX 123 KB)

Additional file 2: **Enrichment of Gene Ontologies corresponding to genes differentially expressed after membrane cholesterol loading (enrichment p value <0.001) {Eden, 2007 #287; Eden, 2009 #286}.** (DOCX 24 KB)

Additional file 3: **Genes differentially expressed after membrane cholesterol loading of cultured hippocampal neurons (t test, p < 0.05) and in postmortem AD brains from Braak stages 0 to VI (data of {Bossers, 2010 #269}; ANOVA, p < 0.05).** (DOCX 44 KB)

Additional file 4: **Gene Ontologies for the genes from cluster 1.** (DOCX 17 KB)

Additional file 5: **Gene Ontologies for the genes from cluster 4.** (DOCX 16 KB)

Additional file 6: **Membrane cholesterol loading causes neuronal EEA1-positive early endosomes enlargement but does not impact on their number, as assessed by confocal microscopy.** (A) and (B) Representative confocal images of cortical neurons (DIV 14) stained with an anti-EEA1 antibody in control conditions (A) or after cholesterol loading (B). (C) Mean number of endosomes per soma (ns stands for p > 0.05 in unpaired Student’s t-test). (D) Mean endosomal size (*** stands for p<0.001, Mann-Whitney test). (PPTX 187 KB)

## Abbreviations

AD: Alzheimer’s disease
APP: Amyloid Precursor Protein
Aβ: Amyloid-beta
MBCD: Methyl-beta-cyclodextrin
HMG-CoA: 3-hydroxy-3-methylglutaryl-coenzyme A
APOE: Apolipoprotein E
GO: Gene Ontology
PIP2: Phopshatidylinositol 4,5-bisphosphate
TEM: Transmission electron microscopy
ILV: Intraluminal small vesicles.

## References

[1] Duyckaerts C, Delatour B, Potier MC (2009). Classification and basic pathology of Alzheimer disease. Acta Neuropathol.

[2] Haass C, Kaether C, Thinakaran G, Sisodia S (2012). Trafficking and proteolytic processing of APP. Cold Spring Harb Perspect Med.

[3] Duyckaerts C, Potier MC, Delatour B (2008). Alzheimer disease models and human neuropathology: similarities and differences. Acta Neuropathol.

[4] Di Paolo G, Kim TW (2011). Linking Lipids to alzheimer’s disease: cholesterol and beyond. Nat Rev Neurosci.

[5] Cutler RG, Kelly J, Storie K, Pedersen WA, Tammara A, Hatanpaa K, Troncoso JC, Mattson MP (2004). Involvement of oxidative stress-induced abnormalities in ceramide and cholesterol metabolism in brain aging and Alzheimer's disease. Proc Natl Acad Sci U S A.

[6] Xiong H, Callaghan D, Jones A, Walker DG, Lue LF, Beach TG, Sue LI, Woulfe J, Xu H, Stanimirovic DB, Zhang W (2008). Cholesterol retention in Alzheimer's brain is responsible for high beta- and gamma-secretase activities and Abeta production. Neurobiol Dis.

[7] Lazar AN, Bich C, Panchal M, Desbenoit N, Petit VW, Touboul D, Dauphinot L, Marquer C, Laprevote O, Brunelle A, Duyckaerts C (2012). Time-of-flight secondary ion mass spectrometry (TOF-SIMS) imaging reveals cholesterol overload in the cerebral cortex of Alzheimer disease patients. Acta Neuropathol.

[8] Strittmatter WJ, Weisgraber KH, Huang DY, Dong LM, Salvesen GS, Pericak-Vance M, Schmechel D, Saunders AM, Goldgaber D, Roses AD (1993). Binding of human apolipoprotein E to synthetic amyloid beta peptide: isoform-specific effects and implications for late-onset Alzheimer disease. Proc Natl Acad Sci U S A.

[9] Bouillot C, Prochiantz A, Rougon G, Allinquant B (1996). Axonal amyloid precursor protein expressed by neurons in vitro is present in a membrane fraction with caveolae-like properties. J Biol Chem.

[10] Cordy JM, Hussain I, Dingwall C, Hooper NM, Turner AJ (2003). Exclusively targeting beta-secretase to lipid rafts by GPI-anchor addition up-regulates beta-site processing of the amyloid precursor protein. Proc Natl Acad Sci U S A.

[11] Simons M, Keller P, De Strooper B, Beyreuther K, Dotti CG, Simons K (1998). Cholesterol depletion inhibits the generation of beta-amyloid in hippocampal neurons. Proc Natl Acad Sci U S A.

[12] Marquer C, Devauges V, Cossec JC, Liot G, Lecart S, Saudou F, Duyckaerts C, Leveque-Fort S, Potier MC (2011). Local cholesterol increase triggers amyloid precursor protein-Bace1 clustering in lipid rafts and rapid endocytosis. FASEB J.

[13] Cossec JC, Simon A, Marquer C, Moldrich RX, Leterrier C, Rossier J, Duyckaerts C, Lenkei Z, Potier MC (2010). Clathrin-dependent APP endocytosis and Abeta secretion are highly sensitive to the level of plasma membrane cholesterol. Biochim Biophys Acta.

[14] Barrett PJ, Song Y, Van Horn WD, Hustedt EJ, Schafer JM, Hadziselimovic A, Beel AJ, Sanders CR (2012). The amyloid precursor protein has a flexible transmembrane domain and binds cholesterol. Science.

[15] Hoglund K, Blennow K (2007). Effect of HMG-CoA reductase inhibitors on beta-amyloid peptide levels: implications for Alzheimer's disease. CNS Drugs.

[16] Pac-Soo C, Lloyd DG, Vizcaychipi MP, Ma D (2011). Statins: the role in the treatment and prevention of Alzheimer's neurodegeneration. J Alzheimers Dis JAD.

[17] Cenedella RJ (2009). Cholesterol synthesis inhibitor U18666A and the role of sterol metabolism and trafficking in numerous pathophysiological processes. Lipids.

[18] Hui L, Chen X, Geiger JD (2012). Endolysosome involvement in LDL cholesterol-induced Alzheimer's disease-like pathology in primary cultured neurons. Life Sci.

[19] Lopez CA, de Vries AH, Marrink SJ (2011). Molecular mechanism of cyclodextrin mediated cholesterol extraction. PLoS Comput Biol.

[20] Bossers K, Wirz KT, Meerhoff GF, Essing AH, van Dongen JW, Houba P, Kruse CG, Verhaagen J, Swaab DF (2010). Concerted changes in transcripts in the prefrontal cortex precede neuropathology in Alzheimer's disease. Brain.

[21] Cataldo AM, Peterhoff CM, Troncoso JC, Gomez-Isla T, Hyman BT, Nixon RA (2000). Endocytic pathway abnormalities precede amyloid beta deposition in sporadic Alzheimer's disease and Down syndrome: differential effects of APOE genotype and presenilin mutations. Am J Pathol.

[22] Cataldo AM, Petanceska S, Terio NB, Peterhoff CM, Durham R, Mercken M, Mehta PD, Buxbaum J, Haroutunian V, Nixon RA (2004). Abeta localization in abnormal endosomes: association with earliest Abeta elevations in AD and Down syndrome. Neurobiol Aging.

[23] Dai J, Buijs RM, Kamphorst W, Swaab DF (2002). Impaired axonal transport of cortical neurons in Alzheimer's disease is associated with neuropathological changes. Brain Res.

[24] Smith KD, Kallhoff V, Zheng H, Pautler RG (2007). In vivo axonal transport rates decrease in a mouse model of Alzheimer's disease. NeuroImage.

[25] Wang N, Silver DL, Thiele C, Tall AR (2001). ATP-binding cassette transporter A1 (ABCA1) functions as a cholesterol efflux regulatory protein. J Biol Chem.

[26] Braak H, Braak E (1991). Neuropathological stageing of Alzheimer-related changes. Acta Neuropathol.

[27] Di Paolo G, De Camilli P (2006). Phosphoinositides in cell regulation and membrane dynamics. Nature.

[28] Chang-Ileto B, Frere SG, Chan RB, Voronov SV, Roux A, Di Paolo G (2011). Synaptojanin 1-mediated PI(4,5)P2 hydrolysis is modulated by membrane curvature and facilitates membrane fission. Dev Cell.

[29] Chun YS, Shin S, Kim Y, Cho H, Park MK, Kim TW, Voronov SV, Di Paolo G, Suh BC, Chung S (2010). Cholesterol modulates ion channels via down-regulation of phosphatidylinositol 4,5-bisphosphate. J Neurochem.

[30] Cossec JC, Lavaur J, Berman DE, Rivals I, Hoischen A, Stora S, Ripoll C, Mircher C, Grattau Y, Olivomarin JC, de Chaumont F, Lecourtois M, Antonarakis SE, Veltman JA, Delabar JM, Duyckaerts C, Di Paolo G, Potier MC (2012). Trisomy for synaptojanin1 in Down syndrome is functionally linked to the enlargement of early endosomes. Hum Mol Genet.

[31] Wilson JM, de Hoop M, Zorzi N, Toh BH, Dotti CG, Parton RG (2000). EEA1, a tethering protein of the early sorting endosome, shows a polarized distribution in hippocampal neurons, epithelial cells, and fibroblasts. Mol Biol Cell.

[32] Stokin GB, Lillo C, Falzone TL, Brusch RG, Rockenstein E, Mount SL, Raman R, Davies P, Masliah E, Williams DS, Goldstein LS (2005). Axonopathy and transport deficits early in the pathogenesis of Alzheimer's disease. Science.

[33] Kaether C, Skehel P, Dotti CG (2000). Axonal membrane proteins are transported in distinct carriers: a two-color video microscopy study in cultured hippocampal neurons. Mol Biol Cell.

[34] Goldsbury C, Mocanu MM, Thies E, Kaether C, Haass C, Keller P, Biernat J, Mandelkow E, Mandelkow EM (2006). Inhibition of APP trafficking by tau protein does not increase the generation of amyloid-beta peptides. Traffic.

[35] Rodrigues EM, Weissmiller AM, Goldstein LS (2012). Enhanced beta-secretase processing alters APP axonal transport and leads to axonal defects. Hum Mol Genet.

[36] Roux JC, Zala D, Panayotis N, Borges-Correia A, Saudou F, Villard L (2012). Modification of Mecp2 dosage alters axonal transport through the Huntingtin/Hap1 pathway. Neurobiol Dis.

[37] Araki Y, Kawano T, Taru H, Saito Y, Wada S, Miyamoto K, Kobayashi H, Ishikawa HO, Ohsugi Y, Yamamoto T, Matsuno K, Kinjo M, Suzuki T (2007). The novel cargo Alcadein induces vesicle association of kinesin-1 motor components and activates axonal transport. EMBO J.

[38] Colin E, Zala D, Liot G, Rangone H, Borrell-Pages M, Li XJ, Saudou F, Humbert S (2008). Huntingtin phosphorylation acts as a molecular switch for anterograde/retrograde transport in neurons. EMBO J.

[39] Chen H, Yang J, Low PS, Cheng JX (2008). Cholesterol level regulates endosome motility via Rab proteins. Biophys J.

[40] Rocha N, Kuijl C, van der Kant R, Janssen L, Houben D, Janssen H, Zwart W, Neefjes J (2009). Cholesterol sensor ORP1L contacts the ER protein VAP to control Rab7-RILP-p150 Glued and late endosome positioning. J Cell Biol.

[41] Lebrand C, Corti M, Goodson H, Cosson P, Cavalli V, Mayran N, Faure J, Gruenberg J (2002). Late endosome motility depends on lipids via the small GTPase Rab7. EMBO J.

[42] Kimura N, Okabayashi S, Ono F (2012). Dynein dysfunction disrupts intracellular vesicle trafficking bidirectionally and perturbs synaptic vesicle docking via endocytic disturbances a potential mechanism underlying age-dependent impairment of cognitive function. Am J Pathol.

[43] Szodorai A, Kuan YH, Hunzelmann S, Engel U, Sakane A, Sasaki T, Takai Y, Kirsch J, Muller U, Beyreuther K, Brady S, Morfini G, Kins S (2009). APP anterograde transport requires Rab3A GTPase activity for assembly of the transport vesicle. J Neurosci.

[44] Klein WL, Stine WB, Teplow DB (2004). Small assemblies of unmodified amyloid beta-protein are the proximate neurotoxin in Alzheimer's disease. Neurobiol Aging.

[45] Okochi M, Tagami S, Yanagida K, Takami M, Kodama TS, Mori K, Nakayama T, Ihara Y, Takeda M (2013). Gamma-secretase modulators and presenilin 1 mutants act differently on presenilin/gamma-secretase function to cleave abeta42 and abeta43. Cell Rep.

[46] Kukar TL, Ladd TB, Robertson P, Pintchovski SA, Moore B, Bann MA, Ren Z, Jansen-West K, Malphrus K, Eggert S, Maruyama H, Cottrell BA, Das P, Basi GS, Koo EH, Golde TE (2011). Lysine 624 of the amyloid precursor protein (APP) is a critical determinant of amyloid beta peptide length: support for a sequential model of gamma-secretase intramembrane proteolysis and regulation by the amyloid beta precursor protein (APP) juxtamembrane region. J Biol Chem.

[47] Saher G, Simons M (2010). Cholesterol and myelin biogenesis. Sub Cell Biochem.

[48] Jurevics H, Largent C, Hostettler J, Sammond DW, Matsushima GK, Kleindienst A, Toews AD, Morell P (2002). Alterations in metabolism and gene expression in brain regions during cuprizone-induced demyelination and remyelination. J Neurochem.

[49] Hudry E, Van Dam D, Kulik W, De Deyn PP, Stet FS, Ahouansou O, Benraiss A, Delacourte A, Bougneres P, Aubourg P, Cartier N (2010). Adeno-associated virus gene therapy with cholesterol 24-hydroxylase reduces the amyloid pathology before or after the onset of amyloid plaques in mouse models of Alzheimer's disease. Mol Ther.

[50] Ittner LM, Gotz J (2011). Amyloid-beta and tau–a toxic pas de deux in Alzheimer's disease. Nat Rev Neurosci.

[51] Ittner LM, Ke YD, Delerue F, Bi M, Gladbach A, van Eersel J, Wolfing H, Chieng BC, Christie MJ, Napier IA, Eckert A, Staufenbiel M, Hardeman E, Götz J (2010). Dendritic function of tau mediates amyloid-beta toxicity in Alzheimer's disease mouse models. Cell.

[52] Yao J, Ho D, Calingasan NY, Pipalia NH, Lin MT, Beal MF (2012). Neuroprotection by cyclodextrin in cell and mouse models of Alzheimer disease. J Exp Med.

[53] Barbero-Camps E, Fernandez A, Martinez L, Fernandez-Checa JC, Colell A (2013). APP/PS1 mice overexpressing SREBP-2 exhibit combined Abeta accumulation and tau pathology underlying Alzheimer's disease. Hum Mol Genet.

[54] de Chaumont F, Dallongeville S, Chenouard N, Herve N, Pop S, Provoost T, Meas-Yedid V, Pankajakshan P, Lecomte T, Le Montagner Y, Lagache T, Dufour A, Olivo-Marin JC (2012). Icy: an open bioimage informatics platform for extended reproducible research. Nat Methods.

[55] Eden E, Lipson D, Yogev S, Yakhini Z (2007). Discovering motifs in ranked lists of DNA sequences. PLoS Comput Biol.

[56] Eden E, Navon R, Steinfeld I, Lipson D, Yakhini Z (2009). GOrilla: a tool for discovery and visualization of enriched GO terms in ranked gene lists. BMC Bioinforma.

