# Supplementary material for: Increasing membrane cholesterol of neurons in culture recapitulates Alzheimer’s disease early phenotypes
Source: Mol Neurodegener. 2014 Dec 18;9:60. doi: 10.1186/1750-1326-9-60 (PMC4280040; doi:10.1186/1750-1326-9-60)
Supplement: Supplementary file 1 — Additional file 1: Significantly differentially expressed genes after cholesterol treatment of cultured hippocampal neurons (t test, p < 0.05). (DOCX 123 KB) [file 13024_2014_566_MOESM1_ESM.docx]

**Additional file 1.** Significantly differentially expressed genes after cholesterol treatment of cultured hippocampal neurons (t test, p < 0.05).

| **Gene acronym** | **Gene Name** | **Mean ratio cholesterol/ control** | **pvalue**  **(t-test)** |
| --- | --- | --- | --- |
| *A2m* | alpha 2 macroglobulin | 1.7 | 0.02196 |
| *Aacs* | acetoacetyl CoA synthetase | 0.31 | 0.02304 |
| *Abca1* | ATP binding cassette_ sub family A ABC1_ member 1 | 2.89 | 0.04487 |
| *Abca3* | ATP binding cassette_ sub family A ABC1_ member 3 | 0.77 | 0.03841 |
| *Abca7* | ATP binding cassette_ sub family A ABC1_ member 7 | 0.27 | 0 |
| *Abca8b* | ATP binding cassette_ sub family C CFTR/MRP_ member 2 | 1.3 | 0.00906 |
| *Abcc2* | ATP binding cassette_ sub family C CFTR/MRP_ member 5 | 2.99 | 0.00523 |
| *Abcc5* | ATP binding cassette_ sub family C CFTR/MRP_ member 8 | 0.79 | 0.03672 |
| *Abcc8* | ATP-binding cassette, sub-family C (CFTR/MRP), member 8 | 0.78 | 0.00381 |
| *Abcf1* | ATP binding cassette_ sub family F GCN20_ member 1 | 0.73 | 0.01299 |
| *Abcf2* | ATP binding cassette_ sub family F GCN20_ member 2 | 0.72 | 0.01985 |
| *Abhd8* | abhydrolase domain containing 8 | 0.62 | 0.00231 |
| *Abi2* | abl interactor 2 | 1.19 | 0.00123 |
| *Abl1* | c abl oncogene 1_ receptor tyrosine kinase | 0.5 | 0.00567 |
| *Abl2* | v abl Abelson murine leukemia viral oncogene homolog 2 arg_ Abelson related gene | 0.71 | 0.01714 |
| *Acaa1* | acetyl Coenzyme A acyltransferase 1 | 0.74 | 0.00731 |
| *Acaca* | acetyl coenzyme A carboxylase alpha | 0.63 | 0.00486 |
| *Acadl* | acyl Coenzyme A dehydrogenase_ long chain | 1.86 | 0.02444 |
| *Acat2* | acetyl Coenzyme A acetyltransferase 2 | 0.32 | 0.00235 |
| *Acn9* | ACN9 homolog S. cerevisiae | 1.54 | 0.01172 |
| *Aco1* | aconitase 1_ soluble | 0.81 | 0.00662 |
| *Acrbp* | acrosin binding protein | 0.67 | 0.00664 |
| *Acsbg1* | acyl CoA synthetase bubblegum family member 1 | 1.77 | 0.00334 |
| *Acsl5* | acyl CoA synthetase long chain family member 5 | 0.68 | 0.04573 |
| *Acss1* | acyl CoA synthetase short chain family member 1 | 2.18 | 0.0384 |
| *Actb* | actin_ beta | 1.01 | 0.01072 |
| *Actc1* | actin_ alpha_ cardiac muscle 1 | 0.66 | 0.0047 |
| *Actr1a* | ARP1 actin related protein 1 homolog A_ centractin alpha yeast | 0.86 | 0.0081 |
| *Actr6* | ARP6 actin related protein 6 homolog yeast | 1.3 | 0.03881 |
| *Acyp1* | acylphosphatase 1_ erythrocyte common type | 1.29 | 0.0174 |
| *Adam10* | ADAM metallopeptidase domain 10 | 0.37 | 0.03034 |
| *Adamts3* | ADAM metallopeptidase with thrombospondin type 1_ motif 3 | 1.56 | 0.0499 |
| *Adamts9* | a disintegrin like and metalloprotease reprolysin type with thrombospondin type 1 motif_ 9 | 1.74 | 0.01058 |
| *Adarb1* | adenosine deaminase_ RNA specific_ B1 | 1.56 | 0.01105 |
| *Adcy2* | adenylate cyclase 2 brain | 1.99 | 0.01003 |
| *Add2* | adducin 2 beta | 0.69 | 0.02067 |
| *Adipor1* | adiponectin receptor 1 | 0.84 | 0.03387 |
| *Adipor2* | adiponectin receptor 2 | 0.55 | 0.03107 |
| *Adm* | adrenomedullin | 0.32 | 0.03763 |
| *Adnp* | activity dependent neuroprotector homeobox | 0.77 | 0.01432 |
| *Adora1* | adenosine A1 receptor | 0.65 | 0.02226 |
| *Adss* | adenylosuccinate synthase | 1.17 | 0.04059 |
| *Aebp2* | AE binding protein 2 | 0.84 | 0.025 |
| *Aer61* | glycosyltransferase Aer61 | 2.49 | 0.00165 |
| *Afaf* | Acrosome formation associated factor | 0.49 | 0.01872 |
| *Aff2* | AF4/FMR2 family_ member 2 | 1.54 | 0.0063 |
| *Aff4* | AF4/FMR2 family_ member 4 | 1.23 | 0.04739 |
| *Aggf1* | angiogenic factor with G patch and FHA domains 1 | 1.3 | 0.02443 |
| *Agl* | amylo 1_6 glucosidase_ 4 alpha glucanotransferase | 0.82 | 0.00652 |
| *Agpat6* | 1 acylglycerol 3 phosphate O acyltransferase 6 lysophosphatidic acid acyltransferase_ zeta | 0.76 | 0.00864 |
| *Agt* | angiotensinogen serpin peptidase inhibitor_ clade A_ member 8 | 1.76 | 0.02852 |
| *Agtr1a* | angiotensin II receptor_ type 1a | 1.27 | 0.02391 |
| *Agtrl1* | apelin receptor | 2.02 | 0.02361 |
| *Ak3l1* | adenylate kinase 3 like 1 | 0.62 | 0.01436 |
| *Akap13* | A kinase PRKA anchor protein 13 | 0.7 | 0.0279 |
| *Akap5* | A kinase PRKA anchor protein 5 | 1.49 | 0.02569 |
| *Akap8l* | A kinase PRKA anchor protein 8 like | 0.81 | 0.00388 |
| *Akap9* | A kinase PRKA anchor protein yotiao 9 | 1.14 | 0.00884 |
| *Alcam* | activated leukocyte cell adhesion molecule | 1.62 | 0.02122 |
| *Aldh3a2* | aldehyde dehydrogenase 3 family_ member A2 | 0.84 | 0.01766 |
| *Alg1* | asparagine linked glycosylation 1_ beta 1_4 mannosyltransferase homolog S. cerevisiae | 0.53 | 0.01298 |
| *Alg3* | asparagine linked glycosylation 3_ alpha 1_3 mannosyltransferase homolog S. cerevisiae | 0.72 | 0.01183 |
| *Alg5* | asparagine linked glycosylation 5_ dolichyl phosphate beta glucosyltransferase homolog S. cerevisiae | 1.27 | 0.04652 |
| *Alg8* | asparagine linked glycosylation 8_ alpha 1_3 glucosyltransferase homolog S. cerevisiae | 0.83 | 0.02427 |
| *Alkbh2* | alkB_ alkylation repair homolog 2 E. coli | 1.84 | 0.0061 |
| *Alms1* | Alstrom syndrome 1 homolog human | 1.22 | 0.04345 |
| *Aloxe3* | arachidonate lipoxygenase 3 | 0.49 | 0.01476 |
| *Als2* | amyotrophic lateral sclerosis 2 juvenile homolog human | 1.48 | 0.00035 |
| *Amacr* | alpha methylacyl CoA racemase | 1.66 | 0.00292 |
| *Amd1* | adenosylmethionine decarboxylase 1 | 0.79 | 0.03195 |
| *Angel2* | angel homolog 2 Drosophila | 1.27 | 0.04628 |
| *Angpt1* | angiopoietin 1 | 2.31 | 0.00576 |
| *Ankrd10* | ankyrin repeat domain 10 | 0.71 | 0.03707 |
| *Ankrd11* | ankyrin repeat domain 11 | 0.7 | 0.0026 |
| *Ankrd24* | ankyrin repeat domain 24 | 1.33 | 0.04835 |
| *Ankrd42* | ankyrin repeat domain 42 | 1.4 | 0.03006 |
| *Ankrd50* | ankyrin repeat domain 50 | 0.76 | 0.01906 |
| *Anxa4* | annexin A4 | 1.83 | 0.00137 |
| *Ap2a2* | adaptor related protein complex 2_ alpha 2 subunit | 1.34 | 0.01015 |
| *Ap2b1* | adaptor related protein complex 2_ beta 1 subunit | 1.33 | 0.04805 |
| *Ap3b1* | adaptor related protein complex 3_ beta 1 subunit | 0.81 | 0.03316 |
| *Ap3m1* | adaptor related protein complex 3_ mu 1 subunit | 1.52 | 0.00927 |
| *Ap3s1* | adaptor related protein complex 3_ sigma 1 subunit | 1.24 | 0.02768 |
| *Apba3* | amyloid beta A4 precursor protein binding_ family A_ member 3 | 0.75 | 0.03836 |
| *Apbb1* | amyloid beta A4 precursor protein binding_ family B_ member 1 Fe65 | 0.98 | 0.04791 |
| *Apeg3* | antisense paternally expressed gene 3 | 1.31 | 0.02328 |
| *Apln* | apelin | 1.53 | 0.01024 |
| *Aprin* | PDS5, regulator of cohesion maintenance, homolog B (S. cerevisiae) | 0.83 | 0.01974 |
| *Apxl* | shroom family member 2 | 1.99 | 0.01126 |
| *Aqp2* | aquaporin 2 collecting duct | 1.45 | 0.01679 |
| *Aqp4* | aquaporin 4 | 2.78 | 0.00181 |
| *Ard1* | N(alpha)-acetyltransferase 10, NatA catalytic subunitNalpha acetyltransferase 10 | 0.91 | 0.03662 |
| *Arf4* | ADP ribosylation factor 4 | 1.45 | 0.0017 |
| *Arf6* | ADP ribosylation factor 6 | 0.48 | 0.03248 |
| *Arfgef1* | ADP ribosylation factor guanine nucleotide exchange factor 1brefeldin A inhibited | 0.81 | 0.01023 |
| *Arg1* | arginase_ liver | 1.9 | 0.00096 |
| *Arhgap1* | Rho GTPase activating protein 1 | 0.85 | 0.04638 |
| *Arhgap21* | Rho GTPase activating protein 21 | 0.71 | 0.00657 |
| *Arhgef1* | Rho guanine nucleotide exchange factor GEF 1 | 0.7 | 0.03216 |
| *Arhgef18* | rho/rac guanine nucleotide exchange factor GEF 18 | 0.6 | 0.01894 |
| *Arhgef19* | Rho guanine nucleotide exchange factor GEF 19 | 1.56 | 0.03957 |
| *Arhgef3* | Rho guanine nucleotide exchange factor GEF 3 | 2.66 | 0.0131 |
| *Arhgef9* | Cdc42 guanine nucleotide exchange factor GEF 9 | 1.48 | 0.02896 |
| *Arid2* | AT rich interactive domain 2 Arid rfx like | 0.82 | 0.03007 |
| *Arid5b* | AT rich interactive domain 5B Mrf1 like | 1.55 | 0.02006 |
| *Arl2* | ADP ribosylation factor like 2 | 1.45 | 0.04555 |
| *Arl6* | ADP ribosylation factor like 6 | 0.72 | 0.03382 |
| *Armc6* | armadillo repeat containing 6 | 0.87 | 0.04294 |
| *Armc8* | armadillo repeat containing 8 | 0.51 | 0.01761 |
| *Armcx2* | armadillo repeat containing_ X linked 2 | 0.76 | 0.03803 |
| *Armet* | arginine rich_ mutated in early stage tumors | 0.49 | 0.02981 |
| *Arpc3* | actin related protein 2/3 complex_ subunit 3 | 0.98 | 0.00542 |
| *Arpc5l* | actin related protein 2/3 complex_ subunit 5 like | 0.8 | 0.03756 |
| *Arrdc1* | arrestin domain containing 1 | 0.81 | 0.04468 |
| *Arsk* | arylsulfatase family_ member K | 1.51 | 0.00138 |
| *Art1* | ADP ribosyltransferase 1 | 1.28 | 0.00787 |
| *Arts1* | endoplasmic reticulum aminopeptidase 1 | 1.34 | 0.01239 |
| *Arv1* | ARV1 homolog S. cerevisiae | 0.83 | 0.00807 |
| *Asam* | adipocyte specific adhesion molecule | 1.29 | 0.01396 |
| *Asb2* | ankyrin repeat and SOCS box containing 2 | 2.26 | 0.0056 |
| *Asb6* | ankyrin repeat and SOCS box containing 6 | 0.76 | 0.01787 |
| *Ascc3* | activating signal cointegrator 1 complex subunit 3 | 0.67 | 0.01877 |
| *Asf1a* | ASF1 anti silencing function 1 homolog A S. cerevisiae | 0.79 | 0.01665 |
| *Asl* | argininosuccinate lyase | 0.79 | 0.03742 |
| *Asmtl* | acetylserotonin O methyltransferase like | 0.85 | 0.04634 |
| *Asph* | aspartate beta hydroxylase | 0.55 | 0.03918 |
| *Asrgl1* | asparaginase like 1 | 2.09 | 0.04257 |
| *Atad3a* | ATPase family_ AAA domain containing 3A | 0.8 | 0.02725 |
| *Atg16l1* | ATG16 autophagy related 16 like 1 S. cerevisiae | 0.78 | 0.0421 |
| *Atg4b* | ATG4 autophagy related 4 homolog B S. cerevisiae | 0.8 | 0.00329 |
| *Atp1a1* | ATPase_ Na+/K+ transporting_ alpha 1 polypeptide | 1.46 | 0.03292 |
| *Atp1a2* | ATPase_ Na+/K+ transporting_ alpha 2 polypeptide | 1.53 | 0.02823 |
| *Atp1b2* | ATPase_ Na+/K+ transporting_ beta 2 polypeptide | 1.24 | 0.03072 |
| *Atp1b3* | ATPase_ Na+/K+ transporting_ beta 3 polypeptide | 0.86 | 0.02411 |
| *Atp2a2* | ATPase_ Ca++ transporting_ cardiac muscle_ slow twitch 2 | 0.74 | 0.04448 |
| *Atp2c1* | ATPase_ Ca++ transporting_ type 2C_ member 1 | 1.3 | 0.0097 |
| *Atp5g2* | ATP synthase_ H+ transporting_ mitochondrial F0 complex_ subunit C2 subunit 9 | 0.96 | 0.02683 |
| *Atp5j* | ATP synthase_ H+ transporting_ mitochondrial F0 complex_ subunit F6 | 1.4 | 0.01416 |
| *Atp6ap1* | ATPase_ H+ transporting_ lysosomal accessory protein 1 | 0.84 | 0.03911 |
| *Atp6v1c2* | ATPase_ H+ transporting_ lysosomal V1 subunit C2 | 0.34 | 0.01001 |
| *Atp6v1e1* | ATPase_ H+ transporting_ lysosomal V1 subunit E1 | 1.4 | 0.0037 |
| *Atp8b2* | Atpase_ class I_ type 8B_ member 2 | 0.75 | 0.02256 |
| *Atxn1* | ataxin 1 | 1.74 | 0.01185 |
| *Aven* | apoptosis_ caspase activation inhibitor | 0.83 | 0.01364 |
| *Avp* | arginine vasopressin | 0.62 | 0.03254 |
| *Axin1* | axin 1 | 0.65 | 0.01075 |
| *Azin1* | antizyme inhibitor 1 | 1.56 | 0.00075 |
| *B3galt3* | UDP-GalNAc:betaGlcNAc beta 1,3-galactosaminyltransferase, polypeptide 1 | 0.84 | 0.04678 |
| *B4galt6* | UDP Gal:betaGlcNAc beta 1_4 galactosyltransferase_ polypeptide 6 | 1.23 | 0.00247 |
| *Bai2* | brain specific angiogenesis inhibitor 2 | 0.59 | 0.00651 |
| *Baiap2* | BAI1 associated protein 2 | 0.63 | 0.0239 |
| *Bat2* | HLA B associated transcript 2 | 0.66 | 0.01091 |
| *Bat4* | HLA B associated transcript 4 | 1.63 | 0.01222 |
| *Baz1a* | bromodomain adjacent to zinc finger domain_ 1A | 0.62 | 0.00822 |
| *Bcas1* | breast carcinoma amplified sequence 1 | 2.74 | 0.01001 |
| *Bcat1* | branched chain aminotransferase 1_ cytosolic | 0.78 | 0.00363 |
| *Bcl2l1* | Bcl2 like 1 | 0.75 | 0.00177 |
| *Bcl2l2* | Bcl2 like 2 | 1.18 | 0.04065 |
| *Bcmo1* | beta carotene 15_15' monooxygenase 1 | 2.04 | 5.00E-05 |
| *Bcs1l* | BCS1 like yeast | 0.72 | 0.01813 |
| *Bdnf* | brain derived neurotrophic factor | 1.35 | 0.01808 |
| *Bfsp1* | beaded filament structural protein 1 | 1.85 | 0.01168 |
| *Bhlhb3* | basic helix-loop-helix family, member e41 | 1.57 | 0.04141 |
| *Bicd2* | bicaudal D homolog 2 Drosophila | 0.88 | 0.0156 |
| *Birc6* | baculoviral IAP repeat containing 6 | 0.71 | 0.02509 |
| *Blvrb* | biliverdin reductase B flavin reductase NADPH | 1.3 | 0.00865 |
| *Bmpr1a* | bone morphogenetic protein receptor_ type IA | 1.44 | 0.02151 |
| *Bnip3l* | BCL2/adenovirus E1B interacting protein 3 like | 1.25 | 0.00367 |
| *Bop1* | block of proliferation 1 | 0.6 | 0.02594 |
| *Brca2* | breast cancer 2 | 1.29 | 0.01188 |
| *Brd2* | bromodomain containing 2 | 1.37 | 0.03395 |
| *Brd4* | bromodomain containing 4 | 0.73 | 0.01413 |
| *Brf1* | BRF1 homolog_ subunit of RNA polymerase III transcription initiation factor IIIB S. cerevisiae | 0.67 | 0.00537 |
| *Bri3* | brain protein I3 | 0.76 | 0.02607 |
| *Brinp3* | family with sequence similarity 5, member C | 2.73 | 0.00519 |
| *Brp44l* | brain protein 44 like | 1.15 | 0.01036 |
| *Bysl* | bystin like | 0.71 | 0.03277 |
| *Bzw1* | basic leucine zipper and W2 domains 1 | 0.77 | 0.01355 |
| *C1galt1c1* | C1GALT1 specific chaperone 1 | 1.11 | 0.03496 |
| *C1qb* | complement component 1_ q subcomponent_ B chain | 2.19 | 0.03978 |
| *C1qbp* | complement component 1_ q subcomponent binding protein | 0.83 | 0.01734 |
| *C3* | complement component 3 | 0.54 | 0.03835 |
| *C3orf6h* | coiled-coil domain containing 50 | 1.43 | 0.01314 |
| *Cab39* | calcium binding protein 39 | 0.84 | 0.0359 |
| *Cabc1* | chaperone_ ABC1 activity of bc1 complex homolog S. pombe | 2.27 | 0.0263 |
| *Cabp1* | calcium binding protein 1 | 0.47 | 0.0361 |
| *Cabp7* | calcium binding protein 7 | 0.38 | 0.01971 |
| *Cacna1g* | calcium channel_ voltage dependent_ T type_ alpha 1G subunit | 0.59 | 0.00445 |
| *Cacnb2* | calcium channel_ voltage dependent_ beta 2 subunit | 1.74 | 0.01141 |
| *Cacnb3* | calcium channel_ voltage dependent_ beta 3 subunit | 0.66 | 0.00041 |
| *Cad* | carbamoyl phosphate synthetase 2_ aspartate transcarbamylase_ and dihydroorotase | 0.69 | 0.03414 |
| *Calb1* | calbindin 1 | 1.36 | 0.03261 |
| *Calcr* | calcitonin receptor | 0.44 | 0.01074 |
| *Camk2a* | calcium/calmodulin dependent protein kinase II alpha | 1.49 | 0.039 |
| *Camk4* | calcium/calmodulin dependent protein kinase IV | 1.5 | 0.02915 |
| *Capzb* | capping protein actin filament muscle Z line_ beta | 0.69 | 0.00178 |
| *Car9* | carbonic anhydrase 9 | 1.26 | 0.01549 |
| *Cart* | CART prepropeptide | 1.52 | 0.03895 |
| *Cbfa2t1* | runt-related transcription factor 1; translocated to, 1 (cyclin D-related) | 0.46 | 0.00816 |
| *Cbfa2t2* | core binding factor_ runt domain_ alpha subunit 2; translocated to_ 2 | 0.8 | 0.03071 |
| *Cbl27* | trinucleotide repeat containing 6b | 1.36 | 0.00787 |
| *Cbll1* | Cas Br M murine ecotropic retroviral transforming sequence like 1 | 1.36 | 0.00705 |
| *Ccbl1* | cysteine conjugate beta lyase_ cytoplasmic | 0.68 | 0.00353 |
| *Ccdc12* | coiled coil domain containing 12 | 1.46 | 0.04291 |
| *Ccdc21* | coiled coil domain containing 21 | 0.66 | 0.00152 |
| *Ccdc5* | HAUS augmin-like complex, subunit 1 | 1.36 | 0.01259 |
| *Cck* | cholecystokinin | 1.36 | 0.04674 |
| *Ccl2* | chemokine C C motif ligand 2 | 0.17 | 5.00E-05 |
| *Ccne1* | cyclin E1 | 0.76 | 0.01534 |
| *Ccne2* | cyclin E2 | 1.65 | 0.02911 |
| *Ccng1* | cyclin G1 | 0.71 | 0.01868 |
| *Ccni* | cyclin I | 1.48 | 0.00502 |
| *Cct8* | chaperonin containing Tcp1_ subunit 8 theta | 1.39 | 0.02025 |
| *Cd248* | CD248 molecule_ endosialin | 0.4 | 0.04762 |
| *Cd9* | CD9 molecule | 0.78 | 0.04234 |
| *Cdadc1* | cytidine and dCMP deaminase domain containing 1 | 1.29 | 0.03154 |
| *Cdc14a* | CDC14 cell division cycle 14 homolog A S. cerevisiae | 1.46 | 0.01023 |
| *Cdc23* | CDC23 cell division cycle 23_ yeast_ homolog | 1.38 | 0.03571 |
| *Cdc27* | cell division cycle 27 homolog S. cerevisiae | 0.62 | 0.02014 |
| *Cdc34* | cell division cycle 34 homolog S. cerevisiae | 0.62 | 0.03581 |
| *Cdc42bpb* | CDC42 binding protein kinase beta DMPK like | 0.81 | 0.04643 |
| *Cdc42se1* | CDC42 small effector 1 | 0.73 | 0.00621 |
| *Cdh10* | cadherin 10 | 2.11 | 0.00422 |
| *Cdh7* | cadherin 7_ type 2 | 1.53 | 0.03327 |
| *Cdh8* | cadherin 8 | 1.7 | 0.01973 |
| *Cdig1l* |  | 0.83 | 0.04745 |
| *Cdk105* | CDK105 protein | 1.29 | 0.0013 |
| *Cdk5* | cyclin dependent kinase 5 | 0.7 | 0.04382 |
| *Cdk7* | cyclin dependent kinase 7 | 0.7 | 0.00653 |
| *Cdkn1a* | cyclin dependent kinase inhibitor 1A | 0.33 | 0.04604 |
| *Cdkn1b* | cyclin dependent kinase inhibitor 1B | 1.58 | 0.04832 |
| *Cdon* | Cdon homolog mouse | 1.49 | 0.04381 |
| *Cebpb* | CCAAT/enhancer binding protein C/EBP_ beta | 0.59 | 0.02258 |
| *Ceecam1* | cerebral endothelial cell adhesion molecule | 0.71 | 0.00073 |
| *Cel* | carboxyl ester lipase | 2.17 | 0.02763 |
| *Centb1* | ArfGAP with coiled-coil, ankyrin repeat and PH domains 1 | 0.51 | 0.02854 |
| *Centb5* | ArfGAP with coiled-coil, ankyrin repeat and PH domains 3 | 0.49 | 0.04948 |
| *Centg2* | centaurin_ gamma 2 | 0.66 | 0.02002 |
| *Centg3* | ArfGAP with GTPase domain, ankyrin repeat and PH domain 3 | 0.59 | 0.00957 |
| *Cep68* | centrosomal protein 68kDa | 0.79 | 0.02569 |
| *Chchd1* | coiled coil helix coiled coil helix domain containing 1 | 1.32 | 0.00391 |
| *Chchd4* | coiled coil helix coiled coil helix domain containing 4 | 0.74 | 0.04405 |
| *Chd1* | chromodomain helicase DNA binding protein 1 | 0.74 | 0.03784 |
| *Chd2* | chromodomain helicase DNA binding protein 2 | 0.79 | 0.00544 |
| *Chd4* | chromodomain helicase DNA binding protein 4 | 0.67 | 0.0062 |
| *Chd6* | chromodomain helicase DNA binding protein 6 | 1.17 | 0.03134 |
| *Chgb* | chromogranin B | 1.33 | 0.0095 |
| *Chn1* | chimerin chimaerin 1 | 1.19 | 0.02666 |
| *Chordc1* | cysteine and histidine rich domain CHORD containing 1 | 0.61 | 0.0039 |
| *Chp* | calcium binding protein p22 | 0.32 | 0.00041 |
| *Chrac1* | chromatin accessibility complex 1 | 1.86 | 0.01724 |
| *Chrna10* | cholinergic receptor_ nicotinic_ alpha 10 | 0.66 | 0.03629 |
| *Chst12* | carbohydrate sulfotransferase 12 | 1.19 | 0.03049 |
| *Chsy1* | chondroitin sulfate synthase 1 | 0.62 | 0.02428 |
| *Chuk* | conserved helix loop helix ubiquitous kinase | 0.45 | 0.00829 |
| *Ciapin1* | cytokine induced apoptosis inhibitor 1 | 0.36 | 0.00041 |
| *Cipar1* | prostate androgen-regulated mucin-like protein 1 | 2.05 | 0.00236 |
| *Cited1* | Cbp/p300 interacting transactivator with Glu/Asp rich carboxy terminal domain 1 | 1.65 | 0.02931 |
| *Ckap1* | tubulin folding cofactor B | 0.67 | 0.03413 |
| *Ckap4* | cytoskeleton associated protein 4 | 0.81 | 0.01173 |
| *Clcn6* | chloride channel 6 | 0.78 | 0.04381 |
| *Clpb* | ClpB caseinolytic peptidase B homolog E. coli | 0.57 | 0.00287 |
| *Clptm1* | cleft lip and palate associated transmembrane protein 1 | 0.78 | 0.0045 |
| *Clu* | clusterin | 1.98 | 0.04732 |
| *Cmkor1* | chemokine (C-X-C motif) receptor 7 | 0.61 | 0.00561 |
| *Cml1* | camello like 1 | 1.73 | 0.02839 |
| *Cnnm3* | cyclin M3 | 0.69 | 0.03496 |
| *Cnr1* | cannabinoid receptor 1 brain | 1.36 | 0.0026 |
| *Cntn3* | contactin 3 plasmacytoma associated | 1.96 | 0.0041 |
| *Cntrob* | centrobin, centrosomal BRCA2 interacting protein | 1.61 | 0.02804 |
| *Cog3* | component of oligomeric golgi complex 3 | 0.84 | 0.00698 |
| *Cog8* | component of oligomeric golgi complex 8 | 0.81 | 0.04453 |
| *Col11a1* | collagen_ type XI_ alpha 1 | 1.42 | 0.04641 |
| *Col11a2* | collagen_ type XI_ alpha 2 | 0.49 | 0.00577 |
| *Col16a1* | collagen_ type XVI_ alpha 1 | 0.69 | 0.00208 |
| *Col18a1* | collagen_ type XVIII_ alpha 1 | 0.51 | 0.01984 |
| *Col1a2* | collagen_ type I_ alpha 2 | 0.61 | 0.03722 |
| *Col5a1* | collagen_ type V_ alpha 1 | 0.57 | 0.0375 |
| *Col5a2* | collagen_ type V_ alpha 2 | 1.43 | 0.01611 |
| *Col9a2* | collagen_ type IX_ alpha 2 | 1.72 | 0.04973 |
| *Commd10* | COMM domain containing 10 | 1.33 | 2.00E-04 |
| *Commd4* | COMM domain containing 4 | 1.08 | 0.02279 |
| *Copa* | coatomer protein complex subunit alpha | 0.74 | 0.03616 |
| *Copb1* | coatomer protein complex_ subunit beta 1 | 0.88 | 0.00852 |
| *Cops7a* | COP9 constitutive photomorphogenic homolog subunit 7A Arabidopsis | 1.62 | 0.00667 |
| *Coq6* | coenzyme Q6 homolog yeast | 0.81 | 0.00615 |
| *Coro1a* | coronin_ actin binding protein 1A | 0.65 | 0.02142 |
| *Coro7* | coronin 7 | 0.76 | 0.01721 |
| *Cort* | cortistatin | 0.47 | 0.00566 |
| *Cotl1* | coactosin like 1 Dictyostelium | 0.85 | 0.02347 |
| *Cova1* | ecto-NOX disulfide-thiol exchanger 2 | 2.1 | 0.03476 |
| *Cpeb4* | cytoplasmic polyadenylation element binding protein 4 | 1.19 | 0.00457 |
| *CPG2* | synaptic nuclear envelope 1 | 1.98 | 0.00065 |
| *Cplx2* | complexin 2 | 1.37 | 0.03975 |
| *Cpne5* | copine V | 1.96 | 0.00276 |
| *Cpsf1* | cleavage and polyadenylation specific factor 1 | 0.85 | 0.03458 |
| *Cpt1b* | carnitine palmitoyltransferase 1b_ muscle | 0.66 | 0.02936 |
| *Crabp1* | cellular retinoic acid binding protein 1 | 1.88 | 0.04621 |
| *Crabp2* | cellular retinoic acid binding protein 2 | 0.5 | 0.00897 |
| *Crebl1* | activating transcription factor 6 beta | 0.51 | 0.03104 |
| *Crkl* | v crk sarcoma virus CT10 oncogene homolog avian like | 0.65 | 0.0104 |
| *Crlz1* | UTP3, small subunit (SSU) processome component, homolog (S. cerevisiae) | 0.82 | 0.04838 |
| *Crot* | carnitine O octanoyltransferase | 1.31 | 0.00682 |
| *Cryab* | crystallin_ alpha B | 1.98 | 0.01368 |
| *Crygd* | crystallin_ gamma D | 1.53 | 0.02456 |
| *Cryl1* | crystallin_ lambda 1 | 1.67 | 0.00449 |
| *Cse1l* | chromosome segregation 1 like S. cerevisiae | 0.81 | 0.01116 |
| *Csk* | c src tyrosine kinase | 0.73 | 0.00083 |
| *Csnk1d* | casein kinase 1_ delta | 0.8 | 0.03836 |
| *Csnk1g2* | casein kinase 1_ gamma 2 | 0.72 | 0.01806 |
| *Csnk2b* | casein kinase 2_ beta subunit | 0.79 | 0.00608 |
| *Csrp2* | cysteine and glycine rich protein 2 | 1.62 | 0.0288 |
| *Cstf3* | cleavage stimulation factor_ 3' pre RNA_ subunit 3 | 0.6 | 0.02875 |
| *Ctdp1* | CTD carboxy terminal domain_ RNA polymerase II_ polypeptide A phosphatase_ subunit 1 | 0.61 | 0.04984 |
| *Cte1* | acyl-CoA thioesterase 1 | 2.32 | 0.04634 |
| *Ctnna2* | catenin cadherin associated protein_ alpha 2 | 1.45 | 0.0259 |
| *Ctnnb1* | catenin cadherin associated protein_ beta 1 | 1.42 | 0.03571 |
| *Ctnnd1* | catenin cadherin associated protein_ delta 1 | 1.35 | 0.032 |
| *Ctrc* | chymotrypsin C caldecrin | 0.56 | 0.03117 |
| *Ctsb* | cathepsin B | 0.99 | 0.04465 |
| *Ctsd* | cathepsin D | 0.81 | 3.00E-04 |
| *Ctsk* | cathepsin K | 0.61 | 0.04261 |
| *Cul2* | cullin 2 | 0.67 | 0.03761 |
| *Cul3* | cullin 3 | 0.79 | 0.01457 |
| *Cul4b* | cullin 4B | 1.56 | 0.00945 |
| *Cutc* | cutC copper transporter homolog E.coli | 0.73 | 0.02179 |
| *Cx39* | gap junction protein, delta 4 | 1.74 | 0.01536 |
| *Cxadr* | coxsackie virus and adenovirus receptor | 1.26 | 0.03994 |
| *Cxcl12* | chemokine C X C motif ligand 12 stromal cell derived factor 1 | 1.55 | 0.02595 |
| *Cybasc3* | cytochrome b_ ascorbate dependent 3 | 1.73 | 0.03649 |
| *Cycs* | cytochrome c_ somatic | 0.77 | 0.01871 |
| *Cyln2* | CAP-GLY domain containing linker protein 2 | 0.86 | 0.00099 |
| *Cyp26b1* | cytochrome P450_ family 26_ subfamily b_ polypeptide 1 | 0.5 | 0.0075 |
| *Cyp51* | cytochrome P450_ subfamily 51 | 0.48 | 0.00698 |
| *D123* | cell division cycle 123 | 1.18 | 0.03638 |
| *Daam1* | dishevelled associated activator of morphogenesis 1 | 0.71 | 0.01694 |
| *Dad1* | defender against cell death 1 | 0.73 | 0.00937 |
| *Dag1* | dystroglycan 1 dystrophin associated glycoprotein 1 | 0.96 | 0.02621 |
| *Dapk2* | death associated kinase 2 | 0.66 | 0.02863 |
| *Dapk3* | death associated protein kinase 3 | 0.67 | 0.04104 |
| *Dars* | aspartyl tRNA synthetase | 1.35 | 0.0015 |
| *Daxx* | death domain associated protein | 0.79 | 0.02402 |
| *Dbn1* | drebrin 1 | 0.64 | 1.00E-05 |
| *Dchs1* | dachsous 1 Drosophila | 0.86 | 0.04366 |
| *Dclre1a* | DNA cross link repair 1A_ PSO2 homolog S. cerevisiae | 0.85 | 0.04096 |
| *Dctn1* | dynactin 1 | 0.61 | 0.0128 |
| *Dctn4* | dynactin 4 | 0.61 | 0.02816 |
| *Dd5* | ubiquitin protein ligase E3 component n-recognin 5 | 0.77 | 0.0116 |
| *Ddit4* | DNA damage inducible transcript 4 | 0.57 | 0.0055 |
| *Ddx21a* | DEAD (Asp-Glu-Ala-Asp) box polypeptide 21 | 0.55 | 0.03646 |
| *Ddx47* | DEAD Asp Glu Ala Asp box polypeptide 47 | 1.21 | 0.00959 |
| *Ddx56* | DEAD Asp Glu Ala Asp box polypeptide 56 | 0.82 | 0.01284 |
| *Deaf1* | deformed epidermal autoregulatory factor 1 Drosophila | 0.81 | 0.0083 |
| *Decr1* | 2_4 dienoyl CoA reductase 1_ mitochondrial | 1.65 | 0.00631 |
| *Dek* | DEK oncogene | 1.26 | 0.01877 |
| *Dffa* | DNA fragmentation factor_ alpha subunit | 0.72 | 0.04288 |
| *Dgcr6* | DiGeorge syndrome critical region gene 6 | 0.79 | 0.00997 |
| *Dhcr7* | 7 dehydrocholesterol reductase | 0.41 | 0.0278 |
| *Dhrs1* | dehydrogenase/reductase SDR family member 1 | 0.7 | 1.00E-04 |
| *Dhx15* | DEAH Asp Glu Ala His box polypeptide 15 | 0.81 | 0.04029 |
| *Dhx30* | DEAH Asp Glu Ala His box polypeptide 30 | 0.64 | 0.01889 |
| *Dhx35* | DEAH Asp Glu Ala His box polypeptide 35 | 0.53 | 0.00855 |
| *Dhx57* | DEAH Asp Glu Ala Asp/His box polypeptide 57 | 0.65 | 0.04707 |
| *Dio2* | deiodinase_ iodothyronine_ type II | 0.64 | 0.00895 |
| *Dio3* | deiodinase_ iodothyronine_ type III | 1.52 | 0.03153 |
| *Dirc2* | disrupted in renal carcinoma 2 human | 0.76 | 0.0025 |
| *Dkk3* | dickkopf homolog 3 Xenopus laevis | 0.78 | 0.00536 |
| *Dlst* | dihydrolipoamide S succinyltransferase E2 component of 2 oxo glutarate complex | 0.68 | 0.00087 |
| *Dmn* | synemin, intermediate filament protein | 1.89 | 0.01786 |
| *Dmpk* | dystrophia myotonica-protein kinase | 0.53 | 0.04577 |
| *Dmwd* | dystrophia myotonica_ WD repeat containing | 0.71 | 0.00988 |
| *Dmxl1* | Dmx-like 1 | 1.24 | 4.00E-05 |
| *Dnah7* | dynein_ axonemal_ heavy polypeptide 7 | 2.05 | 0.02781 |
| *Dnajb1* | DnaJ Hsp40 homolog_ subfamily B_ member 1 | 0.77 | 0.0352 |
| *Dnajc10* | DnaJ Hsp40 homolog_ subfamily C_ member 10 | 0.79 | 0.00077 |
| *Dnajc3* | DnaJ Hsp40 homolog_ subfamily C_ member 3 | 0.6 | 0.02708 |
| *Dnajc8* | DnaJ Hsp40 homolog_ subfamily C_ member 8 | 1.33 | 0.00481 |
| *Dnajc9* | DnaJ Hsp40 homolog_ subfamily C_ member 9 | 1.36 | 0.03404 |
| *Dncic2* | dynein cytoplasmic 1 intermediate chain 2 | 1.38 | 0.04333 |
| *Dnm1* | dynamin 1 | 0.76 | 0.04698 |
| *Dnmt1* | DNA cytosine 5 methyltransferase 1 | 0.65 | 0.00108 |
| *Doc2g* | double C2_ gamma | 0.27 | 0.04163 |
| *Dok3* | docking protein 3 | 0.49 | 0.00359 |
| *Dolpp1* | dolichyl pyrophosphate phosphatase 1 | 0.47 | 0.00135 |
| *Dpf2* | D4_ zinc and double PHD fingers family 2 | 0.66 | 2.00E-05 |
| *Dph5* | DPH5 homolog S. cerevisiae | 1.65 | 0.00417 |
| *Dpm2* | dolichyl phosphate mannosyltransferase polypeptide 2_ regulatory subunit | 1.2 | 0.01292 |
| *Dpp3* | dipeptidylpeptidase 3 | 0.81 | 0.02015 |
| *Dpp6* | dipeptidylpeptidase 6 | 1.29 | 0.03535 |
| *Dpy19l1* | dpy 19 like 1 C. elegans | 0.71 | 0.02947 |
| *Dr1* | down regulator of transcription 1 | 0.67 | 0.02714 |
| *Dscaml1* | Down syndrome cell adhesion molecule like 1 | 0.49 | 0.00926 |
| *Dscr2* | proteasome (prosome, macropain) assembly chaperone 1 | 0.84 | 0.04518 |
| *Dspp* | dentin sialophosphoprotein | 1.53 | 0.01229 |
| *Dtx2* | deltex homolog 2 Drosophila | 0.65 | 0.03007 |
| *Dus2l* | dihydrouridine synthase 2 like_ SMM1 homolog S. cerevisiae | 1.32 | 0.00293 |
| *Dusp11* | dual specificity phosphatase 11 RNA/RNP complex 1 interacting | 0.58 | 0.0195 |
| *Dusp18* | dual specificity phosphatase 18 | 0.84 | 0.01271 |
| *Dusp5* | dual specificity phosphatase 5 | 0.41 | 0.0438 |
| *Dvl1* | dishevelled_ dsh homolog 1 Drosophila | 0.5 | 0.00714 |
| *Dyrk2* | dual specificity tyrosine Y phosphorylation regulated kinase 2 | 0.61 | 0.0419 |
| *Dzip1* | DAZ interacting protein 1 | 0.71 | 0.00012 |
| *Edg1* | endothelin receptor type A | 2.23 | 0.03224 |
| *Ednra* | endothelin receptor type B | 0.65 | 0.01417 |
| *Ednrb* | eukaryotic elongation factor 2 kinase | 0.56 | 0.04629 |
| *Eef2k* | eukaryotic elongation factor-2 kinase | 0.78 | 0.00757 |
| *Ehmt2* | euchromatic histone lysine N methyltransferase 2 | 0.7 | 0.00027 |
| *Eif2ak4* | eukaryotic translation initiation factor 2 alpha kinase 4 | 0.74 | 0.02213 |
| *Eif3s10* | eukaryotic translation initiation factor 3_ subunit 10 theta | 1.29 | 0.00489 |
| *Eif3s6ip* | eukaryotic translation initiation factor 3_ subunit 6 interacting protein | 0.75 | 7.00E-04 |
| *Eif4a2* | eukaryotic translation initiation factor 4A2 | 1.15 | 0.00031 |
| *Eif4ebp2* | eukaryotic translation initiation factor 4E binding protein 2 | 1.39 | 0.01815 |
| *Eif4g1* | eukaryotic translation initiation factor 4, gamma 1 | 0.61 | 0.00419 |
| *Eif4g3* | eukaryotic translation initiation factor 4 gamma_ 3 | 1.99 | 0.00933 |
| *Elavl1* | ELAV embryonic lethal_ abnormal vision_ Drosophila like 1 Hu antigen R | 0.81 | 0.00723 |
| *Elf2* | E74 like factor 2 | 0.45 | 0.04987 |
| *Elmo1* | engulfment and cell motility 1 | 1.56 | 0.04332 |
| *Elmo2* | engulfment and cell motility 2 | 0.68 | 0.01946 |
| *Elmo3* | engulfment and cell motility 3 | 0.57 | 0.02853 |
| *Elmod1* | ELMO/CED 12 domain containing 1 | 1.4 | 0.01806 |
| *Elovl7* | ELOVL family member 7_ elongation of long chain fatty acids yeast | 1.56 | 0.02444 |
| *Enc1* | ectodermal neural cortex 1 | 1.42 | 0.01019 |
| *Endog* | endonuclease G | 0.75 | 0.00335 |
| *Eno1* | enolase 1_ alpha | 0.88 | 0.02128 |
| *Eno3* | enolase 3_ beta_ muscle | 0.7 | 0.00337 |
| *Enpp5* | ectonucleotide pyrophosphatase/phosphodiesterase 5 | 1.21 | 0.00335 |
| *Ep400* | E1A binding protein p400 | 1.29 | 0.02562 |
| *Epha5* | EphA5 | 1.82 | 0.00741 |
| *Ephb2* | Eph receptor B2 | 0.69 | 0.03942 |
| *Erbb2* | v erb b2 erythroblastic leukemia viral oncogene homolog 2_ neuro/glioblastoma derived oncogene homolog avian | 1.9 | 0.00181 |
| *Ercc5* | excision repair cross complementing rodent repair deficiency_ complementation group 5 | 1.47 | 0.03749 |
| *Erp29* | endoplasmic reticulum protein 29 | 0.76 | 0.02145 |
| *Esd* | esterase D/formylglutathione hydrolase | 1.31 | 0.0032 |
| *Etf1* | eukaryotic translation termination factor 1 | 0.81 | 0.01131 |
| *Etfa* | electron transfer flavoprotein_ alpha polypeptide | 0.8 | 0.04139 |
| *Etfb* | electron transfer flavoprotein_ beta polypeptide | 1.33 | 0.01993 |
| *Etnk1* | ethanolamine kinase 1 | 0.63 | 0.03707 |
| *Etv1* | ets variant 1 | 1.45 | 0.01613 |
| *Etv5* | ets variant 5 | 0.64 | 0.03003 |
| *Exoc4* | exocyst complex component 4 | 0.65 | 0.01243 |
| *Exoc6* | exocyst complex component 6 | 0.72 | 0.02645 |
| *Exosc9* | exosome component 9 | 0.77 | 0.00723 |
| *F3* | coagulation factor III thromboplastin_ tissue factor | 0.54 | 0.01817 |
| *Fam3c* | family with sequence similarity 3_ member C | 1.24 | 0.00403 |
| *Faslg* | Fas ligand TNF superfamily_ member 6 | 0.54 | 0.00268 |
| *Fasn* | fatty acid synthase | 0.47 | 0.00295 |
| *Fat3* | FAT tumor suppressor homolog 3 Drosophila | 1.37 | 0.00956 |
| *Fbl* | fibrillarin | 0.79 | 0.02315 |
| *Fbxl5* | F box and leucine rich repeat protein 5 | 0.58 | 0.03234 |
| *Fbxo21* | F box protein 21 | 0.88 | 0.03383 |
| *Fbxo23* | F box only protein 23 | 0.53 | 0.02291 |
| *Fbxo30* | F box protein 30 | 0.76 | 0.01618 |
| *Fbxo6b* | F-box protein 6 | 0.69 | 0.00579 |
| *Fbxw2* | F box and WD repeat domain containing 2 | 0.78 | 0.04732 |
| *Fbxw5* | F box and WD repeat domain containing 5 | 0.78 | 0.04711 |
| *Fcgr3* | Fc receptor, IgG, low affinity III | 3.34 | 0.00517 |
| *Fcho1* | FCH domain only 1 | 0.42 | 0.00736 |
| *Fdft1* | farnesyl diphosphate farnesyl transferase 1 | 0.38 | 0.03888 |
| *Fem1b* | feminization 1 homolog b C. elegans | 0.86 | 0.04757 |
| *Fgf13* | fibroblast growth factor 13 | 1.56 | 0.00128 |
| *Fgfr1op2* | FGFR1 oncogene partner 2 | 0.78 | 0.03268 |
| *Fgfrl1* | fibroblast growth factor receptor like 1 | 0.86 | 0.02909 |
| *Fhl2* | four and a half LIM domains 2 | 0.55 | 0.00523 |
| *Fjx1* | four jointed box 1 Drosophila | 0.59 | 0.02792 |
| *Fkbp1a* | FK506 binding protein 1a | 0.8 | 0.01209 |
| *Fkbp3* | FK506 binding protein 3 | 1.22 | 0.01539 |
| *Fkbp4* | FK506 binding protein 4 | 0.58 | 0.02697 |
| *Fkbp5* | FK506 binding protein 5 | 0.4 | 0.03693 |
| *Flcn* | folliculin | 1.15 | 0.03854 |
| *Fln29* | TRAF type zinc finger domain containing 1 | 0.77 | 0.00533 |
| *Flnc* | filamin C_ gamma | 1.18 | 0.02844 |
| *Flot1* | flotillin 1 | 0.83 | 0.01452 |
| *Fmod* | fibromodulin | 1.64 | 0.043 |
| *Fmr1* | fragile X mental retardation 1 | 1.4 | 0.01833 |
| *Fn1* | fibronectin 1 | 1.21 | 0.00099 |
| *Fosb* | FBJ osteosarcoma oncogene B | 2.14 | 0.00316 |
| *Foxc2* | forkhead box C2 | 2 | 0.00066 |
| *Foxd4* | forkhead box D4 | 1.59 | 0.00159 |
| *Foxk2* | forkhead box K2 | 0.56 | 0.03464 |
| *Frap1* | mechanistic target of rapamycin (serine/threonine kinase) | 0.85 | 0.04664 |
| *Frs3* | fibroblast growth factor receptor substrate 3 | 2.24 | 0.03606 |
| *Fstl5* | follistatin like 5 | 1.88 | 0.03759 |
| *Fubp3* | far upstream element FUSE binding protein 3 | 0.64 | 0.0323 |
| *Fuca* | fucosidase, alpha-L- 1, tissue | 1.3 | 0.02033 |
| *Fuk* | fucokinase | 0.46 | 0.01037 |
| *Fundc1* | FUN14 domain containing 1 | 1.45 | 0.04399 |
| *Fusip1* | FUS interacting protein serine arginine rich 1 | 0.73 | 0.02866 |
| *Fut9* | fucosyltransferase 9 alpha 1_3 fucosyltransferase | 1.62 | 0.03427 |
| *Fxc1* | fractured callus expressed transcript 1 | 1.84 | 0.0052 |
| *Fxr2h* | fragile X mental retardation, autosomal homolog 2 | 0.63 | 0.03196 |
| *Fxyd5* | FXYD domain containing ion transport regulator 5 | 0.34 | 0.01981 |
| *Fyn* | FYN oncogene related to SRC_ FGR_ YES | 1.58 | 0.02038 |
| *Fzd1* | frizzled homolog 1 Drosophila | 0.79 | 0.04983 |
| *Fzd9* | frizzled homolog 9 Drosophila | 0.5 | 0.02267 |
| *G0s2* | G0/G1switch 2 | 1.44 | 0.01944 |
| *G3bp* | Ras-GTPase-activating protein SH3-domain binding protein 1 | 0.75 | 0.00094 |
| *G6pdx* | glucose-6-phosphate dehydrogenase X-linked | 0.92 | 0.00434 |
| *Gabra1* | gamma aminobutyric acid GABA A receptor_ alpha 1 | 1.63 | 0.00886 |
| *Gabrg1* | gamma aminobutyric acid GABA A receptor_ gamma 1 | 1.77 | 0.01577 |
| *Gad2* | glutamate decarboxylase 2 | 1.27 | 0.04634 |
| *Gale* | UDP galactose 4 epimerase | 0.74 | 0.0307 |
| *Galnt11* | UDP N acetyl alpha D galactosamine:polypeptide N acetylgalactosaminyltransferase 11 GalNAc T11 | 0.77 | 0.00864 |
| *Galnt2* | UDP N acetyl alpha D galactosamine:polypeptide N acetylgalactosaminyltransferase 2 GalNAc T2 | 0.79 | 0.01395 |
| *Galt* | galactose 1 phosphate uridylyltransferase | 0.82 | 0.0458 |
| *Gapdh* | glyceraldehyde 3 phosphate dehydrogenase | 0.79 | 0.00035 |
| *Gas2l1* | growth arrest-specific 2 like 1 | 0.74 | 0.02119 |
| *Gbe1* | glucan 1_4 alpha _ branching enzyme 1 | 1.86 | 7.00E-05 |
| *Gcn1l1* | GCN1 general control of amino acid synthesis 1 like 1 yeast | 0.7 | 0.00061 |
| *Gcs1* | glucosidase 1 | 0.78 | 0.0346 |
| *Gdf1* | growth differentiation factor 1 | 0.61 | 0.04625 |
| *Gdf10* | growth differentiation factor 10 | 1.88 | 0.01601 |
| *Gdf6* | growth differentiation factor 6 | 1.56 | 0.01543 |
| *Gfap* | glial fibrillary acidic protein | 1.61 | 0.02819 |
| *Gfra2* | GDNF family receptor alpha 2 | 0.47 | 0.01046 |
| *Ggh* | gamma glutamyl hydrolase | 1.52 | 0.00806 |
| *Git1* | G protein coupled receptor kinase interacting ArfGAP 1 | 0.74 | 0.01044 |
| *Gja1* | gap junction protein_ alpha 1 | 1.98 | 0.02816 |
| *Gjc1* | gap junction protein, gamma 1 | 1.27 | 0.03173 |
| *Glra2* | glycine receptor_ alpha 2 | 1.46 | 0.00012 |
| *Glt8d1* | glycosyltransferase 8 domain containing 1 | 0.76 | 0.00205 |
| *Gltscr1* | glioma tumor suppressor candidate region gene 1 | 0.73 | 0.00683 |
| *Glud1* | glutamate dehydrogenase 1 | 1.31 | 0.02513 |
| *Gmfg* | glia maturation factor_ gamma | 1.64 | 0.00149 |
| *Gna12* | guanine nucleotide binding protein_ alpha 12 | 0.71 | 0.00695 |
| *Gnai3* | guanine nucleotide binding protein G protein_ alpha inhibiting 3 | 1.24 | 0.04968 |
| *Gnaq* | guanine nucleotide binding protein_ alpha q polypeptide | 0.73 | 0.04839 |
| *Gnas* | GNAS complex locus | 1.77 | 0.01984 |
| *Gne* | glucosamine UDP N acetyl 2 epimerase/N acetylmannosamine kinase | 0.64 | 0.02962 |
| *Gng2* | guanine nucleotide binding protein (G protein), gamma 2 | 1.36 | 0.01735 |
| *Gnrh1* | gonadotropin releasing hormone 1 luteinizing releasing hormone | 0.68 | 0.02029 |
| *Golga2* | golgi autoantigen_ golgin subfamily a_ 2 | 0.9 | 0.03827 |
| *Golph4* | golgi integral membrane protein 4 | 1.43 | 0.03072 |
| *Gorasp2* | golgi reassembly stacking protein 2 | 0.53 | 0.00749 |
| *Gosr1* | golgi SNAP receptor complex member 1 | 2.42 | 0.00059 |
| *Gosr2* | golgi SNAP receptor complex member 2 | 0.4 | 0.00109 |
| *Gpaa1* | glycosylphosphatidylinositol anchor attachment protein 1 homolog yeast | 0.56 | 0.01672 |
| *Gpatc1* | G patch domain containing 1 | 1.47 | 0.01081 |
| *Gpc2* | glypican 2 | 0.83 | 0.03628 |
| *Gpcr12* | G protein coupled receptor 12 | 1.43 | 0.00487 |
| *Gphn* | gephyrin | 1.67 | 0.0076 |
| *Gpm6a* | glycoprotein m6a | 1.43 | 0.00811 |
| *Gpm6b* | glycoprotein m6b | 2.11 | 0.00139 |
| *Gpr108* | G protein coupled receptor 108 | 0.68 | 0.0013 |
| *Gpr125* | G protein-coupled receptor 125 | 0.68 | 0.03578 |
| *Gpr135* | G protein coupled receptor 135 | 0.6 | 0.01742 |
| *Gpr22* | G protein coupled receptor 22 | 1.86 | 0.04757 |
| *Gpr68* | G protein coupled receptor 68 | 1.6 | 0.02935 |
| *Gprc5c* | G protein-coupled receptor, family C, group 5, member C | 1.49 | 0.02782 |
| *Gprk5* | G protein-coupled receptor kinase 5 | 0.56 | 0.01968 |
| *Gprk6* | G protein-coupled receptor kinase 6 | 0.7 | 0.02141 |
| *Gpsm1* | G protein signaling modulator 1 AGS3 like_ C. elegans | 0.79 | 0.00996 |
| *Gpx1* | glutathione peroxidase 1 | 1.32 | 0.02389 |
| *Gramd1a* | GRAM domain containing 1A | 0.63 | 0.01835 |
| *Gria1* | glutamate receptor_ ionotropic_ AMPA 1 | 0.74 | 8.00E-04 |
| *Gria2* | glutamate receptor_ ionotropic_ AMPA 2 | 1.35 | 0.04225 |
| *Grik5* | glutamate receptor_ ionotropic_ kainate 5 | 0.6 | 0.00588 |
| *Grin2c* | glutamate receptor_ ionotropic_ N methyl D aspartate 2C | 0.48 | 0.03434 |
| *Grin2d* | glutamate receptor_ ionotropic_ N methyl D aspartate 2D | 1.92 | 0.01162 |
| *Grm3* | glutamate receptor_ metabotropic 3 | 1.73 | 0.02582 |
| *Grp* | gastrin releasing peptide | 3.12 | 0.00605 |
| *Gs3* | DnaJ (Hsp40) homolog, subfamily C, member 21 | 0.76 | 0.04575 |
| *Gspt1* | G1 to S phase transition 1 | 0.85 | 0.00718 |
| *Gstt2* | glutathione S transferase_ theta 2 | 1.72 | 0.01023 |
| *Gtf2b* | general transcription factor IIB | 1.27 | 0.02455 |
| *Gtf2e2* | general transcription factor IIE_ polypeptide 2_ beta | 0.71 | 0.01871 |
| *Gtf2f2* | general transcription factor IIF_ polypeptide 2 | 0.71 | 0.01744 |
| *Gtf2ird1* | GTF2I repeat domain containing 1 | 0.68 | 0.00025 |
| *Gulp1* | GULP_ engulfment adaptor PTB domain containing 1 | 2.13 | 0.00608 |
| *H2afy* | H2A histone family_ member Y | 0.7 | 0.00249 |
| *Hagh* | hydroxyacyl glutathione hydrolase | 0.74 | 0.0011 |
| *Hao1* | hydroxyacid oxidase glycolate oxidase 1 | 2.17 | 0.00862 |
| *Hapln4* | hyaluronan and proteoglycan link protein 4 | 1.69 | 0.02266 |
| *Has2* | hyaluronan synthase 2 | 1.59 | 0.04082 |
| *Hbb* | hemoglobin_ beta | 0.29 | 0.00014 |
| *Hbp1* | HMG box transcription factor 1 | 1.83 | 0.02098 |
| *Hbs1l* | Hbs1 like S. cerevisiae | 1.16 | 0.04614 |
| *Hbxap* | remodeling and spacing factor 1 | 0.64 | 0.02976 |
| *Hccs* | holocytochrome c synthetase | 0.77 | 0.02449 |
| *Hcn4* | hyperpolarization activated cyclic nucleotide gated potassium channel 4 | 0.69 | 0.01417 |
| *Hdac8* | histone deacetylase 8 | 1.36 | 0.01175 |
| *Hddc3* | HD domain containing 3 | 1.47 | 0.04415 |
| *Hdgfrp2* | hepatoma derived growth factor_ related protein 2 | 0.69 | 0.00942 |
| *Herc1* | hect homologous to the E6 AP UBE3A carboxyl terminus domain and RCC1 CHC1 like domain RLD 1 | 0.7 | 0.04439 |
| *Hes6* | hairy and enhancer of split 6 Drosophila | 0.67 | 0.01298 |
| *Hexb* | hexosaminidase B | 1.31 | 0.02826 |
| *Hexim2* | hexamthylene bis acetamide inducible 2 | 0.58 | 0.01026 |
| *Hgs* | hepatocyte growth factor regulated tyrosine kinase substrate | 0.77 | 0.00809 |
| *Hif1a* | hypoxia inducible factor 1_ alpha subunit basic helix loop helix transcription factor | 0.79 | 0.04887 |
| *Hig1* | HIG1 domain family, member 1A | 0.68 | 0.01286 |
| *Hirip3* | HIRA interacting protein 3 | 1.43 | 0.0396 |
| *Hist1h2bh* | histone cluster 1_ H2bh | 1.45 | 0.0099 |
| *Hist1h2bl* | histone cluster 1_ H2bl | 1.33 | 0.04175 |
| *Hist1h2bm* | histone cluster 1_ H2bm | 1.46 | 0.00427 |
| *Hist1h2bn* | histone cluster 1, H2bn | 1.27 | 0.04113 |
| *Hist1h4b* | histone cluster 1_ H4b | 1.8 | 0.02088 |
| *Hk1* | hexokinase 1 | 0.67 | 0.01672 |
| *Hmbs* | hydroxymethylbilane synthase | 0.72 | 0.00874 |
| *Hmgcr* | 3 hydroxy 3 methylglutaryl Coenzyme A reductase | 0.37 | 0.01876 |
| *Hmgcs1* | 3 hydroxy 3 methylglutaryl Coenzyme A synthase 1 soluble | 0.58 | 0.01397 |
| *Hmgn2* | high mobility group nucleosomal binding domain 2 | 1.22 | 0.02027 |
| *Hmox2* | heme oxygenase decycling 2 | 0.88 | 0.04469 |
| *Hnrpa1* | heterogeneous nuclear ribonucleoprotein A1 | 1 | 0.034 |
| *Hnrpa3* | heterogeneous nuclear ribonucleoprotein A3 | 0.7 | 0.03587 |
| *Hnrpf* | heterogeneous nuclear ribonucleoprotein F | 1.32 | 0.01152 |
| *Hnrpk* | heterogeneous nuclear ribonucleoprotein K | 1.28 | 0.03129 |
| *Hnrpu* | heterogeneous nuclear ribonucleoprotein U | 0.8 | 0.00971 |
| *Homer1* | homer homolog 1 Drosophila | 2.94 | 0.00061 |
| *Hoxa5* | homeo box A5 | 1.78 | 0.01994 |
| *Hoxc6* | homeo box C6 | 1.2 | 0.02756 |
| *Hps1* | Hermansky Pudlak syndrome 1 homolog human | 1.87 | 0.03078 |
| *Hras* | Harvey rat sarcoma virus oncogene | 0.73 | 0.0263 |
| *Hrasls3* | phospholipase A2, group XVI | 3.44 | 0.00522 |
| *Hsd17b1* | hydroxysteroid 17 beta dehydrogenase 1 | 0.56 | 0.04408 |
| *Hspa14* | heat shock protein 14 | 0.7 | 0.00772 |
| *Hspa1a* | heat shock 70kD protein 1A | 0.46 | 0.03207 |
| *Hspa2* | heat shock protein 2 | 1.45 | 0.0292 |
| *Hspa5* | heat shock protein 5 | 0.38 | 0.01676 |
| *Hspb1* | heat shock protein 1 | 1.39 | 0.00088 |
| *Hspca* | heat shock protein 90, alpha (cytosolic), class A member 1 | 1.32 | 0.03526 |
| *Hspd1* | heat shock protein 1 chaperonin | 0.9 | 0.00366 |
| *Hspe1* | heat shock protein 1 chaperonin 10 | 0.89 | 0.00299 |
| *Htr7* | 5 hydroxytryptamine serotonin receptor 7 | 1.89 | 0.01007 |
| *Ibrdc3* | ring finger protein 19B | 0.52 | 0.02298 |
| *Ica1* | islet cell autoantigen 1 | 1.5 | 0.01256 |
| *Id3* | inhibitor of DNA binding 3 | 0.69 | 0.03405 |
| *Idh3g* | isocitrate dehydrogenase 3 NAD_ gamma | 0.89 | 0.00979 |
| *Idi1* | isopentenyl diphosphate delta isomerase 1 | 0.38 | 0.03937 |
| *Ier2* | immediate early response 2 | 0.77 | 0.00553 |
| *Ier5l* | immediate early response 5 like | 0.6 | 0.03693 |
| *Ifi44* | interferon induced protein 44 | 1.29 | 0.03061 |
| *Ifrd2* | interferon related developmental regulator 2 | 0.77 | 0.04806 |
| *Ift74* | intraflagellar transport 74 homolog Chlamydomonas | 1.27 | 0.03773 |
| *Igf1* | insulin like growth factor 1 | 1.92 | 0.01051 |
| *Igf2r* | insulin like growth factor 2 receptor | 0.77 | 0.02185 |
| *Igfbp2* | insulin like growth factor binding protein 2 | 0.54 | 0.00091 |
| *Igfbp3* | insulin like growth factor binding protein 3 | 0.84 | 0.04968 |
| *Igsf1* | immunoglobulin superfamily_ member 1 | 3.15 | 0.00478 |
| *Igsf10* | immunoglobulin superfamily_ member 10 | 1.57 | 0.02426 |
| *Igsf3* | immunoglobulin superfamily_ member 3 | 1.81 | 0.00253 |
| *Il17re* | interleukin 17 receptor E | 1.5 | 0.04236 |
| *Ilkap* | integrin linked kinase associated serine/threonine phosphatase 2C | 0.83 | 0.01796 |
| *Impdh2* | IMP inosine monophosphate dehydrogenase 2 | 0.81 | 0.04747 |
| *Inhbb* | inhibin beta B | 1.39 | 0.00311 |
| *Inpp4b* | inositol polyphosphate 4 phosphatase_ type II | 1.62 | 0.01946 |
| *Inpp5e* | inositol polyphosphate-5-phosphatase E | 0.75 | 0.01313 |
| *Insig1* | insulin induced gene 1 | 0.33 | 0.03321 |
| *Ipmk* | inositol polyphosphate multikinase | 0.55 | 0.00533 |
| *Ipo4* | importin 4 | 0.66 | 0.00342 |
| *Ipo9* | importin 9 | 0.78 | 0.02042 |
| *Iqsec3* | IQ motif and Sec7 domain 3 | 1.43 | 0.02812 |
| *Irf3* | interferon regulatory factor 3 | 0.73 | 0.01582 |
| *Irs1* | insulin receptor substrate 1 | 1.72 | 0.00192 |
| *Irs2* | insulin receptor substrate 2 | 0.61 | 0.03442 |
| *Isg20* | interferon stimulated exonuclease gene 20 | 0.37 | 0.00648 |
| *Itga3* | integrin_ alpha 3 | 0.64 | 0.02151 |
| *Itga6* | integrin_ alpha 6 | 1.51 | 0.04968 |
| *Itgb1* | integrin_ beta 1 | 0.86 | 0.03397 |
| *Itgb3bp* | integrin beta 3 binding protein beta3 endonexin | 1.63 | 0.00491 |
| *Itgb8* | integrin beta 8 | 1.75 | 0.01136 |
| *Itm2b* | integral membrane protein 2B | 1.26 | 0.03254 |
| *Ivns1abp* | influenza virus NS1A binding protein | 0.85 | 0.00894 |
| *Jag2* | jagged 2 | 0.45 | 0.03871 |
| *Jak2* | Janus kinase 2 | 0.81 | 0.00069 |
| *Jam3* | junctional adhesion molecule 3 | 0.41 | 0.04847 |
| *Jmjd1c* | jumonji domain containing 1C | 1.21 | 0.03452 |
| *Josd3* | TATA box binding protein (Tbp)-associated factor, RNA polymerase I, D | 0.61 | 0.03574 |
| *Jun* | Jun oncogene | 1.95 | 0.00368 |
| *Jund* | jun D proto oncogene | 1.5 | 0.03289 |
| *Kalrn* | kalirin_ RhoGEF kinase | 1.41 | 0.0164 |
| *Katna1* | katanin p60 ATPase containing subunit A1 | 0.76 | 0.03218 |
| *Katnal1* | katanin p60 subunit A like 1 | 1.46 | 0.02725 |
| *Kcna2* | potassium voltage gated channel_ shaker related subfamily_ member 2 | 1.51 | 0.02624 |
| *Kcna3* | potassium voltage gated channel_ shaker related subfamily_ member 3 | 1.86 | 0.00023 |
| *Kcna4* | potassium voltage gated channel_ shaker related subfamily_ member 4 | 1.99 | 0.00931 |
| *Kcnj3* | potassium inwardly rectifying channel_ subfamily J_ member 3 | 1.58 | 0.00042 |
| *Kcnj4* | potassium inwardly rectifying channel_ subfamily J_ member 4 | 1.87 | 0.02285 |
| *Kcnk2* | potassium channel_ subfamily K_ member 2 | 1.5 | 0.01144 |
| *Kcnn2* | potassium intermediate/small conductance calcium activated channel_ subfamily N_ member 2 | 0.66 | 0.04104 |
| *Kcnq3* | potassium voltage-gated channel, subfamily Q, member 3 | 2.57 | 0.00855 |
| *Kcnt1* | potassium channel_ subfamily T_ member 1 | 0.28 | 0.01449 |
| *Kctd3* | potassium channel tetramerisation domain containing 3 | 1.3 | 0.04387 |
| *Kctd6* | potassium channel tetramerisation domain containing 6 | 0.32 | 0.00179 |
| *Kdelc2* | KDEL Lys Asp Glu Leu containing 2 | 1.42 | 0.04184 |
| *Kdr* | kinase insert domain receptor | 1.72 | 0.01866 |
| *Khdrbs2* | KH domain containing_ RNA binding_ signal transduction associated 2 | 1.86 | 0.0037 |
| *Khdrbs3* | KH domain containing_ RNA binding_ signal transduction associated 3 | 1.39 | 0.00246 |
| *Kidins220* | kinase D interacting substrate 220 | 0.83 | 4.00E-05 |
| *Kif15* | kinesin family member 15 | 1.68 | 0.02356 |
| *Kif21a* | kinesin family member 21A | 0.86 | 0.03329 |
| *Kif5b* | kinesin family member 5B | 0.64 | 0.00755 |
| *Kif5c* | kinesin family member 5C | 1.53 | 0.03421 |
| *Kitl* | kit ligand | 1.4 | 0.02807 |
| *Klc2* | kinesin light chain 2 | 0.52 | 0.00128 |
| *Klf4* | Kruppel like factor 4 gut | 0.55 | 0.03915 |
| *Klf7* | Kruppel like factor 7 ubiquitous | 0.81 | 0.04351 |
| *Klhl2* | kelch-like 2, Mayven (Drosophila) | 0.64 | 0.00361 |
| *Klhl25* | kelch like 25 Drosophila | 0.68 | 0.0094 |
| *Kns2* | kinesin light chain 1 | 0.74 | 0.03322 |
| *Kpna2* | karyopherin alpha 2 | 0.71 | 0.01144 |
| *Krt1-18* | keratin 18 | 0.73 | 0.02494 |
| *Krtcap2* | keratinocyte associated protein 2 | 0.77 | 0.03995 |
| *Lactb2* | lactamase_ beta 2 | 1.43 | 0.01569 |
| *Lap3* | leucine aminopeptidase 3 | 1.28 | 0.04003 |
| *Larp1* | La ribonucleoprotein domain family_ member 1 | 0.64 | 0.03207 |
| *Las1l* | LAS1 like S. cerevisiae | 0.79 | 0.00557 |
| *Lbr* | lamin B receptor | 0.64 | 0.03795 |
| *Lcat* | lecithin cholesterol acyltransferase | 0.77 | 0.03264 |
| *Lenep* | lens epithelial protein | 1.24 | 0.0146 |
| *Leo1* | Leo1_ Paf1/RNA polymerase II complex component_ homolog S. cerevisiae | 1.22 | 0.04701 |
| *Lepre1* | leucine proline enriched proteoglycan leprecan 1 | 0.89 | 0.00438 |
| *Leprotl1* | leptin receptor overlapping transcript like 1 | 0.77 | 0.00705 |
| *Lgi1* | leucine rich_ glioma inactivated 1 | 1.65 | 0.008 |
| *Lig4* | ligase IV_ DNA_ ATP dependent | 0.73 | 0.02106 |
| *Limk2* | LIM domain kinase 2 | 0.81 | 0.04682 |
| *Lims1* | LIM and senescent cell antigen like domains 1 | 1.46 | 0.00853 |
| *Lin28* | lin 28 homolog C. elegans | 0.34 | 0.02058 |
| *Litaf* | lipopolysaccharide induced TNF factor | 1.28 | 0.0169 |
| *Lix1* | Lix1 homolog chicken | 1.23 | 0.04327 |
| *Lmo3* | LIM domain only 3 | 1.62 | 0.02152 |
| *Lnk* | SH2B adaptor protein 3 | 0.65 | 0.01825 |
| *Lnpep* | leucyl/cystinyl aminopeptidase | 0.75 | 0.03036 |
| *Loxl4* | lysyl oxidase like 4 | 1.54 | 0.02901 |
| *Lpl* | lipoprotein lipase | 1.59 | 0.03274 |
| *Lrdd* | leucine rich repeats and death domain containing | 0.69 | 0.03138 |
| *Lrfn3* | leucine rich repeat and fibronectin type III domain containing 3 | 0.56 | 9.00E-05 |
| *Lrig1* | leucine rich repeats and immunoglobulin like domains 1 | 0.56 | 0.00187 |
| *Lrp10* | low density lipoprotein receptor related protein 10 | 0.72 | 0.00222 |
| *Lrp11* | low density lipoprotein receptor related protein 11 | 1.17 | 0.0018 |
| *Lrp16* | MACRO domain containing 1 | 1.44 | 0.04815 |
| *Lrp2* | low density lipoprotein related protein 2 | 0.71 | 0.02577 |
| *Lrp3* | low density lipoprotein receptor related protein 3 | 0.8 | 0.03742 |
| *Lrpap1* | low density lipoprotein receptor related protein associated protein 1 | 1.35 | 0.00048 |
| *Lrrc16* | leucine rich repeat containing 16A | 0.74 | 0.02703 |
| *Lrrc48* | leucine rich repeat containing 48 | 1.56 | 0.01852 |
| *Lrrc4b* | leucine rich repeat containing 4B | 0.6 | 0.00543 |
| *Lrrc4c* | leucine rich repeat containing 4C | 1.74 | 0.00017 |
| *Lrrc59* | leucine rich repeat containing 59 | 0.74 | 0.00641 |
| *Lrrc8* | leucine rich repeat containing 8A | 0.69 | 0.04925 |
| *Lrrfip2* | leucine rich repeat in FLII interacting protein 2 | 0.84 | 0.01461 |
| *Lrrk2* | leucine rich repeat kinase 2 | 1.82 | 0.04699 |
| *Lrrn1* | leucine rich repeat neuronal 1 | 1.63 | 0.00299 |
| *Lrrn2* | leucine rich repeat neuronal 2 | 0.67 | 0.03909 |
| *Lsm8* | LSM8 homolog_ U6 small nuclear RNA associated S. cerevisiae | 1.58 | 0.00918 |
| *Lta4h* | leukotriene A4 hydrolase | 0.81 | 0.01414 |
| *Ltbp1* | latent transforming growth factor beta binding protein 1 | 0.35 | 0.00626 |
| *Ltbp3* | latent transforming growth factor beta binding protein 2 | 0.83 | 0.03736 |
| *Ly6h* | lymphocyte antigen 6 complex_ locus H | 0.75 | 0.04283 |
| *Lypd3* | Ly6/Plaur domain containing 3 | 1.25 | 0.02476 |
| *Lypla2* | lysophospholipase 2 | 0.81 | 0.00473 |
| *Lyplal1* | lysophospholipase like 1 | 1.59 | 0.01593 |
| *Madh7* | SMAD family member 7 | 1.2 | 0.00335 |
| *Maea* | macrophage erythroblast attacher | 0.67 | 0.04526 |
| *Mafg* | v maf musculoaponeurotic fibrosarcoma oncogene homolog G avian | 0.71 | 0.00703 |
| *Mak10* | MAK10 homolog_ amino acid N acetyltransferase subunit_ S. cerevisiae | 0.81 | 0.00049 |
| *Map1lc3a* | microtubule associated protein 1 light chain 3 alpha | 0.98 | 0.00563 |
| *Map4k3* | mitogen activated protein kinase kinase kinase kinase 3 | 0.72 | 0.04606 |
| *Mapk1* | mitogen activated protein kinase 1 | 0.76 | 0.01659 |
| *Mapk14* | mitogen activated protein kinase 14 | 0.73 | 0.04475 |
| *Mapk6* | mitogen activated protein kinase 6 | 0.77 | 0.0467 |
| *Mapk7* | mitogen-activated protein kinase 7 | 0.72 | 0.0061 |
| *Mapk8ip3* | mitogen activated protein kinase 8 interacting protein 3 | 0.69 | 0.01448 |
| *March1* | membrane associated ring finger C3HC4 1 | 1.82 | 0.01338 |
| *Mark3* | MAP/microtubule affinity regulating kinase 3 | 0.78 | 0.0261 |
| *MAST1* | microtubule associated serine/threonine kinase 1 | 0.66 | 0.0185 |
| *Mat2a* | methionine adenosyltransferase II_ alpha | 0.69 | 0.03273 |
| *Mbd6* | methyl CpG binding domain protein 6 | 0.74 | 0.0248 |
| *Mccc2* | methylcrotonoyl Coenzyme A carboxylase 2 beta | 1.38 | 0.03427 |
| *Mcee* | methylmalonyl CoA epimerase | 1.4 | 0.02141 |
| *Mcf2l* | MCF.2 cell line derived transforming sequence like | 0.68 | 0.01283 |
| *Mcl1* | myeloid cell leukemia sequence 1 | 0.7 | 0.01242 |
| *Mcm3ap* | minichromosome maintenance deficient 3 S. cerevisiae associated protein | 0.74 | 0.02481 |
| *Mcoln1* | mucolipin 1 | 0.76 | 0.02411 |
| *Mecr* | mitochondrial trans 2 enoyl CoA reductase | 0.58 | 0.03168 |
| *Med25* | mediator complex subunit 25 | 0.91 | 0.04532 |
| *Meis2* | Meis homeobox 2 | 1.39 | 0.03603 |
| *Men1* | multiple endocrine neoplasia 1 | 0.47 | 0.00673 |
| *Mepe* | matrix extracellular phosphoglycoprotein | 2.24 | 0.02507 |
| *Mesdc2* | mesoderm development candidate 2 | 1.54 | 0.04526 |
| *Metap1* | methionyl aminopeptidase 1 | 0.77 | 0.02408 |
| *Mettl7a* | methyltransferase like 7A | 1.3 | 0.00951 |
| *Mfap2* | microfibrillar associated protein 2 | 0.52 | 0.03089 |
| *Mfge8* | milk fat globule EGF factor 8 protein | 0.63 | 0.0159 |
| *Mfhas1* | malignant fibrous histiocytoma amplified sequence 1 | 0.66 | 0.01569 |
| *Mfn1* | mitofusin 1 | 0.72 | 0.0166 |
| *Mgat4a* | mannosyl alpha 1_3 glycoprotein beta 1_4 N acetylglucosaminyltransferase_ isozyme A | 1.61 | 0.0435 |
| *Mgmt* | O 6 methylguanine DNA methyltransferase | 0.77 | 0.02861 |
| *Mia1* | melanoma inhibitory activity 1 | 0.59 | 0.00106 |
| *Mk1* | Mk1 protein | 0.73 | 1.00E-05 |
| *Mll* | myeloid/lymphoid or mixed-lineage leukemia 1 | 0.78 | 0.00959 |
| *Mll5* | myeloid/lymphoid or mixed lineage leukemia 5 trithorax homolog_ Drosophila | 1.15 | 0.01491 |
| *Mllt6* | myeloid/lymphoid or mixed lineage leukemia trithorax homolog_ Drosophila; translocated to_ 6 | 0.82 | 0.04539 |
| *Mmp14* | matrix metallopeptidase 14 membrane inserted | 1.72 | 0.00332 |
| *Mmp16* | matrix metallopeptidase 16 | 1.79 | 0.00032 |
| *Mmp2* | matrix metallopeptidase 2 | 0.32 | 0.00385 |
| *Mmrn2* | multimerin 2 | 1.56 | 0.03418 |
| *Morc3* | microrchidia 3 | 0.57 | 0.02271 |
| *Morp1* | mannose P dolichol utilization defect 1 | 0.52 | 0.00035 |
| *Mpdu1* | multiple PDZ domain protein | 1.21 | 0.0456 |
| *Mpdz* | mannose phosphate isomerase mapped | 1.27 | 0.0056 |
| *Mpi* | mannose phosphate isomerase | 0.6 | 0.02451 |
| *Mpp3* | membrane protein, palmitoylated 3 (MAGUK p55 subfamily member 3) | 0.53 | 0.02208 |
| *Mpp4* | membrane protein_ palmitoylated 4 MAGUK p55 subfamily member 4 | 0.56 | 0.02112 |
| *Mre11a* | MRE11 meiotic recombination 11 homolog A S. cerevisiae | 1.35 | 0.01494 |
| *Mrlcb* | mitochondrial ribosomal protein L12 | 1.43 | 1.00E-05 |
| *Mrpl12* | mitochondrial ribosomal protein L12 | 0.82 | 0.02731 |
| *Mrpl48* | mitochondrial ribosomal protein L48 | 1.28 | 0.04893 |
| *Mrpl53* | mitochondrial ribosomal protein L53 | 0.87 | 0.0198 |
| *mrpl9* | mitochondrial ribosomal protein L9 | 1.41 | 0.01432 |
| *Mrps14* | mitochondrial ribosomal protein S14 | 1.39 | 0.02785 |
| *Mrps18c* | mitochondrial ribosomal protein S18C | 1.49 | 0.01248 |
| *Mrps27* | mitochondrial ribosomal protein S27 | 0.72 | 0.00804 |
| *Mrps30* | mitochondrial ribosomal protein S30 | 1.23 | 0.04661 |
| *Mrps33* | mitochondrial ribosomal protein S33 | 1.5 | 0.00495 |
| *Mrps36* | mitochondrial ribosomal protein S36 | 1.18 | 0.02825 |
| *Mrrf* | mitochondrial ribosome recycling factor | 1.31 | 0.017 |
| *Msl31* | male-specific lethal 3 homolog (Drosophila) | 1.51 | 0.00134 |
| *Msto1* | misato homolog 1 Drosophila | 0.8 | 0.03986 |
| *Msx1* | msh homeobox 1 | 2.12 | 0.00229 |
| *Mtap2* | microtubule-associated protein 2 | 1.52 | 0.04231 |
| *Mtap6* | microtubule-associated protein 6 | 0.56 | 0.04549 |
| *Mtdh* | metadherin | 0.92 | 0.03124 |
| *Mterfd3* | MTERF domain containing 3 | 1.56 | 0.01824 |
| *Mtf2* | metal response element binding transcription factor 2 | 0.7 | 0.00315 |
| *Mthfd1* | methylenetetrahydrofolate dehydrogenase NADP+ dependent 1_ methenyltetrahydrofolate cyclohydrolase_ formyltetrahydrofolate synthetase | 0.79 | 0.03608 |
| *Mtmr3* | myotubularin related protein 3 | 0.89 | 0.04864 |
| *Mtmr4* | myotubularin related protein 4 | 0.66 | 0.0259 |
| *Mtmr9* | myotubularin related protein 9 | 0.79 | 0.03869 |
| *Mtvr2* | mammary tumor virus receptor 2 | 0.8 | 0.00419 |
| *Mum1* | melanoma associated antigen mutated 1 | 0.68 | 0.04812 |
| *Mutyh* | mutY homolog E. coli | 0.65 | 0.01076 |
| *Mvd* | mevalonate diphospho decarboxylase | 0.28 | 0.01896 |
| *Mvk* | mevalonate kinase | 0.49 | 0.00199 |
| *Mxd4* | Max dimerization protein 4 | 1.53 | 0.03516 |
| *Mxi1* | MAX interactor 1 | 1.45 | 0.02149 |
| *Mycl1* | v myc myelocytomatosis viral oncogene homolog 1_ lung carcinoma derived avian | 1.34 | 0.02099 |
| *Myl9* | myosin_ light chain 9_ regulatory | 1.39 | 0.04351 |
| *Myo9b* | myosin IXb | 0.69 | 0.03686 |
| *Myst3* | MYST histone acetyltransferase monocytic leukemia 3 | 0.6 | 0.03216 |
| *Nap1l3* | nucleosome assembly protein 1 like 3 | 1.58 | 0.0019 |
| *Nap1l4* | nucleosome assembly protein 1 like 4 | 1.26 | 0.04638 |
| *Naprt1* | nicotinate phosphoribosyltransferase domain containing 1 | 1.35 | 0.03288 |
| *Nat8* | N acetyltransferase 8 | 1.5 | 0.03726 |
| *Ncald* | neurocalcin delta | 1.83 | 0.01747 |
| *Ncam1* | neural cell adhesion molecule 1 | 1.26 | 0.02636 |
| *Ncbp1* | nuclear cap binding protein subunit 1_ 80kDa | 0.81 | 0.01773 |
| *Nck1* | NCK adaptor protein 1 | 0.72 | 0.04941 |
| *Nckap1* | NCK associated protein 1 | 0.76 | 0.00698 |
| *Ncln* | nicalin homolog zebrafish | 0.7 | 0.0154 |
| *Ncoa1* | nuclear receptor coactivator 1 | 1.24 | 0.01104 |
| *Ncoa3* | nuclear receptor coactivator 3 | 0.7 | 0.01772 |
| *Ncoa6* | nuclear receptor coactivator 6 | 0.73 | 0.02664 |
| *Ncor1* | nuclear receptor co repressor 1 | 0.78 | 0.01886 |
| *Ncor2* | nuclear receptor co repressor 2 | 0.61 | 0.00743 |
| *Ndel1* | nuclear distribution gene E like homolog 1 A. nidulans | 0.72 | 0.04115 |
| *Ndfip1* | Nedd4 family interacting protein 1 | 0.88 | 7.00E-04 |
| *Ndufb2* | NADH dehydrogenase ubiquinone 1 beta subcomplex_ 2 | 1.33 | 0.02603 |
| *Ndufb6* | NADH dehydrogenase ubiquinone 1 beta subcomplex_ 6 | 0.76 | 4.00E-05 |
| *Ndufb9* | NADH dehydrogenase ubiquinone 1 beta subcomplex_ 9 | 0.84 | 0.0057 |
| *Ndufc2* | NADH dehydrogenase ubiquinone 1_ subcomplex unknown_ 2 | 1.2 | 0.03101 |
| *Ndufs2* | NADH dehydrogenase ubiquinone Fe S protein 2 | 1.35 | 0.02461 |
| *Necap2* | NECAP endocytosis associated 2 | 0.79 | 0.00654 |
| *Nedd4l* | neural precursor cell expressed_ developmentally down regulated 4 like | 0.9 | 0.04151 |
| *Nedd9* | neural precursor cell expressed_ developmentally down regulated 9 | 1.32 | 0.02761 |
| *Nefl* | neurofilament_ light polypeptide | 1.85 | 0.02011 |
| *Negr1* | neuronal growth regulator 1 | 1.59 | 0.03248 |
| *Nell2* | NEL like 2 chicken | 1.01 | 0.00375 |
| *Neurl2* | neuralized like 2 Drosophila | 1.73 | 0.04868 |
| *Neurod2* | neurogenic differentiation 2 | 0.56 | 0.00353 |
| *Nfat5* | nuclear factor of activated T cells 5 | 0.77 | 0.03209 |
| *Nfkb1* | nuclear factor of kappa light polypeptide gene enhancer in B cells 1 | 1.28 | 0.03527 |
| *Nfkbia* | nuclear factor of kappa light polypeptide gene enhancer in B cells inhibitor_ alpha | 0.71 | 0.01773 |
| *Nfrkb* | nuclear factor related to kappa B binding protein | 0.72 | 0.00357 |
| *Nid67* | putative small membrane protein NID67 | 0.84 | 0.03494 |
| *Nipa2* | non imprinted in Prader Willi/Angelman syndrome 2 homolog human | 0.64 | 0.00649 |
| *Nit1* | nitrilase 1 | 1.31 | 0.03572 |
| *Nkg7* | natural killer cell group 7 sequence | 0.44 | 0.04412 |
| *Nme3* | non metastatic cells 3_ protein expressed in | 1.35 | 0.02847 |
| *Nnat* | neuronatin | 1.05 | 0.04788 |
| *Noc4l* | nucleolar complex associated 4 homolog S. cerevisiae | 0.58 | 0.00747 |
| *Nol5a* | nucleolar protein 5A | 0.72 | 0.01295 |
| *Nolc1* | nucleolar and coiled body phosphoprotein 1 | 0.62 | 0.00118 |
| *Nono* | non POU domain containing_ octamer binding | 1.23 | 0.02297 |
| *Nov* | nephroblastoma overexpressed gene | 1.8 | 0.04922 |
| *Np* | nucleoside phosphorylase | 0.68 | 0.00392 |
| *Npas1* | neuronal PAS domain protein 1 | 1.45 | 0.00044 |
| *Npdc1* | neural proliferation_ differentiation and control_ 1 | 0.97 | 0.00194 |
| *Npff* | neuropeptide FF amide peptide precursor | 0.82 | 0.04309 |
| *Nppc* | natriuretic peptide precursor C | 1.73 | 0.00294 |
| *Npr2* | natriuretic peptide receptor B/guanylate cyclase B atrionatriuretic peptide receptor B | 0.73 | 0.00492 |
| *Nptx1* | neuronal pentraxin 1 | 1.76 | 0.01941 |
| *Npy* | neuropeptide Y | 0.51 | 0.04775 |
| *Nr2f6* | nuclear receptor subfamily 2_ group F_ member 6 | 0.72 | 0.04333 |
| *Nr3c1* | nuclear receptor subfamily 3_ group C_ member 1 | 1.99 | 0.00029 |
| *Nrcam* | neuronal cell adhesion molecule | 0.36 | 0.00335 |
| *Nrxn3* | neurexin 3 | 1.78 | 0.00888 |
| *Nsd1* | nuclear receptor binding SET domain protein 1 | 0.65 | 0.01355 |
| *Nsfl1c* | NSFL1 p97 cofactor p47 | 0.85 | 0.0481 |
| *Nt5c3* | 5' nucleotidase_ cytosolic III | 1.49 | 0.01077 |
| *Ntrk3* | neurotrophic tyrosine kinase_ receptor_ type 3 | 0.76 | 0.00121 |
| *Ntsr2* | neurotensin receptor 2 | 1.7 | 0.01945 |
| *Nub1* | negative regulator of ubiquitin like proteins 1 | 1.31 | 0.0411 |
| *Nubp1* | nucleotide binding protein 1 | 0.8 | 0.02408 |
| *Nucb1* | nucleobindin 1 | 0.71 | 0.03222 |
| *Nudt4* | nudix nucleoside diphosphate linked moiety X type motif 4 | 1.51 | 0.00531 |
| *Nudt7* | nudix nucleoside diphosphate linked moiety X type motif 7 | 0.23 | 0.01679 |
| *Nup205* | nucleoporin 205 | 0.74 | 0.00484 |
| *Nup93* | nucleoporin 93 | 0.76 | 0.01024 |
| *Obfc2b* | oligonucleotide/oligosaccharide binding fold containing 2B | 0.7 | 0.00128 |
| *Olfm1* | olfactomedin 1 | 0.99 | 0.02842 |
| *Olfml2b* | olfactomedin like 2B | 0.77 | 0.02463 |
| *Olr1338* | olfactory receptor 1338 | 4.36 | 0.0393 |
| *Olr1639* | olfactory receptor 1639 | 1.79 | 0.01517 |
| *Olr384* | olfactory receptor 384 | 1.37 | 0.01994 |
| *Olr499* | olfactory receptor 499 | 4.42 | 0.03742 |
| *Olr57* | olfactory receptor 57 | 2.45 | 0.00671 |
| *Oprd1* | opioid receptor_ delta 1 | 1.5 | 0.0076 |
| *Oprl1* | opiate receptor like 1 | 0.52 | 0.01727 |
| *Oprs1* | opioid receptor_ sigma 1 | 0.61 | 0.01599 |
| *ORF19* | WAS protein family homolog | 1.58 | 0.03282 |
| *Osbpl1a* | oxysterol binding protein like 1A | 1.64 | 0.00524 |
| *Osbpl9* | oxysterol binding protein like 9 | 1.35 | 0.03516 |
| *Osgepl1* | O sialoglycoprotein endopeptidase like 1 | 1.28 | 0.0136 |
| *Otud4* | OTU domain containing 4 | 0.77 | 0.01844 |
| *P2rx2* | purinergic receptor P2X_ ligand gated ion channel_ 2 | 1.69 | 0.0215 |
| *Pabpc1* | polyA binding protein_ cytoplasmic 1 | 0.98 | 0.01465 |
| *Pabpc4* | polyA binding protein_ cytoplasmic 4 | 0.79 | 0.04456 |
| *Pabpn1* | polyA binding protein_ nuclear 1 | 0.8 | 0.00325 |
| *Padi2* | peptidyl arginine deiminase_ type II | 2 | 0.04846 |
| *Paf1* | Paf1_ RNA polymerase II associated factor_ homolog S. cerevisiae | 1.29 | 0.0093 |
| *Palm* | paralemmin | 0.83 | 0.00605 |
| *Pank2* | pantothenate kinase 2 Hallervorden Spatz syndrome | 0.57 | 0.04474 |
| *Papd5* | PAP associated domain containing 5 | 0.68 | 0.00301 |
| *Parp1* | poly ADP ribose polymerase 1 | 0.8 | 0.04519 |
| *Pc* | pyruvate carboxylase | 0.9 | 0.03288 |
| *Pcaf* | p300/CBP associated factor | 1.42 | 0.02774 |
| *Pcbp3* | polyrC binding protein 3 | 0.71 | 0.001 |
| *Pcdh17* | protocadherin 17 | 1.4 | 0.01472 |
| *Pcdh9* | protocadherin 9 | 1.42 | 0.03118 |
| *Pcf11* | PCF11_ cleavage and polyadenylation factor subunit_ homolog S. cerevisiae | 1.15 | 0.04242 |
| *Pck2* | phosphoenolpyruvate carboxykinase 2 mitochondrial | 1.47 | 0.00948 |
| *Pcmt1* | protein L isoaspartate D aspartate O methyltransferase 1 | 1.73 | 0.00367 |
| *Pcnxl3* | pecanex like 3 Drosophila | 0.77 | 0.00302 |
| *Pcsk7* | proprotein convertase subtilisin/kexin type 7 | 0.62 | 0.01084 |
| *Pcyt1b* | phosphate cytidylyltransferase 1_ choline_ beta | 1.3 | 0.04603 |
| *Pdcd11* | programmed cell death 11 | 0.68 | 7.00E-04 |
| *Pdcd6ip* | programmed cell death 6 interacting protein | 0.67 | 0.01186 |
| *Pdcl3* | phosducin like 3 | 1.8 | 0.01588 |
| *Pdgfa* | platelet derived growth factor alpha polypeptide | 0.51 | 0.01259 |
| *Pdgfra* | platelet derived growth factor receptor_ alpha polypeptide | 3.09 | 0.00979 |
| *Pdha1* | pyruvate dehydrogenase lipoamide alpha 1 | 1.18 | 0.02641 |
| *Pdhb* | pyruvate dehydrogenase lipoamide beta | 0.86 | 0.03478 |
| *Pdk3* | pyruvate dehydrogenase kinase_ isozyme 3 | 0.71 | 0.01192 |
| *Pdpk1* | 3 phosphoinositide dependent protein kinase 1 | 0.79 | 0.00192 |
| *Pdxk* | pyridoxal pyridoxine_ vitamin B6 kinase | 0.66 | 0.00991 |
| *Pdzk6* | inturned planar cell polarity effector homolog (Drosophila) | 1.16 | 0.0212 |
| *Pebp1* | phosphatidylethanolamine binding protein 1 | 1.02 | 0.03249 |
| *Pecr* | peroxisomal trans 2 enoyl CoA reductase | 1.55 | 0.0329 |
| *Pemt* | phosphatidylethanolamine N methyltransferase | 1.71 | 0.02362 |
| *Pex11b* | peroxisomal biogenesis factor 11 beta | 2.72 | 0.00119 |
| *Pex11c* | peroxisomal biogenesis factor 11 gamma | 2.05 | 0.0114 |
| *Pex13* | peroxisomal biogenesis factor 13 | 0.8 | 0.02543 |
| *Pex6* | peroxisomal biogenesis factor 6 | 0.67 | 0.00459 |
| *Pfkfb1* | 6 phosphofructo 2 kinase/fructose 2_6 biphosphatase 1 | 0.68 | 0.00681 |
| *Pgm3* | phosphoglucomutase 3 | 1.2 | 0.02582 |
| *Pgrmc1* | progesterone receptor membrane component 1 | 1.42 | 0.00337 |
| *Phf17* | PHD finger protein 17 | 0.10 | 0.04389 |
| *Phf5a* | PHD finger protein 5A | 0.75 | 0.01412 |
| *Phlda1* | pleckstrin homology like domain_ family A_ member 1 | 0.62 | 0.00615 |
| *Phlpp* | PH domain and leucine rich repeat protein phosphatase | 1.4 | 0.01817 |
| *Phr1* | pleckstrin homology domain containing, family B (evectins) member 1 | 1.12 | 0.03826 |
| *Phyhd1* | phytanoyl CoA dioxygenase domain containing 1 | 0.51 | 0.03301 |
| *Pigp* | phosphatidylinositol glycan anchor biosynthesis_ class P | 1.42 | 0.00387 |
| *Pigx* | phosphatidylinositol glycan anchor biosynthesis_ class X | 0.74 | 0.00646 |
| *Pik3cd* | phosphatidylinositol 3 kinase catalytic delta polypeptide | 0.69 | 0.04488 |
| *Pik3r1* | phosphoinositide 3 kinase_ regulatory subunit 1 alpha | 1.49 | 0.00279 |
| *Pik3r2* | phosphoinositide 3 kinase_ regulatory subunit 2 beta | 0.8 | 0.04231 |
| *Pin1* | peptidylprolyl cis/trans isomerase_ NIMA interacting 1 | 0.78 | 0.00812 |
| *Pitpnm2* | phosphatidylinositol transfer protein_ membrane associated 2 | 0.75 | 0.01585 |
| *Pkd1* | polycystic kidney disease 1 homolog | 0.62 | 0.00563 |
| *Pkn1* | protein kinase N1 | 0.76 | 0.03125 |
| *Plagl2* | pleiomorphic adenoma gene like 2 | 0.77 | 0.01776 |
| *Plcb1* | phospholipase C_ beta 1 phosphoinositide specific | 1.45 | 0.0471 |
| *Plcg1* | phospholipase C_ gamma 1 | 0.76 | 0.04366 |
| *Plcl1* | phospholipase C like 1 | 1.6 | 0.00273 |
| *Plekha6* | pleckstrin homology domain containing_ family A member 6 | 0.77 | 0.01997 |
| *Plekhb1* | pleckstrin homology domain containing_ family B evectins member 1 | 2.22 | 0.01879 |
| *Plekhc1* | fermitin family homolog 2 (Drosophila) | 0.8 | 0.04667 |
| *Plekhk1* | rhotekin 2 | 3.39 | 0.02464 |
| *Plekhm1* | pleckstrin homology domain containing_ family M with RUN domain member 1 | 2.43 | 0.00054 |
| *Plk4* | polo like kinase 4 Drosophila | 1.27 | 0.0325 |
| *Plod1* | procollagen lysine 1_ 2 oxoglutarate 5 dioxygenase 1 | 0.73 | 0.03975 |
| *Pls3* | plastin 3 T isoform | 1.27 | 0.00134 |
| *Pltp* | phospholipid transfer protein | 1.84 | 0.02009 |
| *Plxna3* | plexin A3 | 0.65 | 0.03914 |
| *Pmch* | pro melanin concentrating hormone | 1.79 | 0.00722 |
| *Pnoc* | prepronociceptin | 5.22 | 0.00299 |
| *Podxl* | podocalyxin like | 1.45 | 0.01147 |
| *Podxl2* | podocalyxin like 2 | 0.82 | 0.04388 |
| *Polb* | polymerase DNA directed_ beta | 0.72 | 0.00141 |
| *Pole3* | polymerase DNA directed_ epsilon 3 p17 subunit | 1.77 | 0.01202 |
| *Polg* | polymerase DNA directed_ gamma | 0.59 | 0.02824 |
| *Polh* | polymerase DNA directed_ eta | 0.56 | 0.00298 |
| *Polr2d* | polymerase RNA II DNA directed polypeptide D | 0.68 | 0.03312 |
| *Polr3e* | polymerase RNA III DNA directed polypeptide E | 0.71 | 0.00969 |
| *Polrmt* | polymerase RNA mitochondrial DNA directed | 0.61 | 0.00884 |
| *Pols* | polymerase DNA directed sigma | 0.73 | 0.00298 |
| *Pomgnt1* | protein O linked mannose beta1_2 N acetylglucosaminyltransferase | 0.65 | 0.04025 |
| *Pomt1* | protein O mannosyltransferase 1 | 0.73 | 0.0024 |
| *Pop7* | processing of precursor 7_ ribonuclease P/MRP subunit S. cerevisiae | 1.66 | 0.00083 |
| *Pou3f3* | POU class 3 homeobox 3 | 1.41 | 0.00071 |
| *Ppan* | peter pan homolog Drosophila | 0.73 | 0.01565 |
| *Ppapdc2* | phosphatidic acid phosphatase type 2 domain containing 2 | 1.39 | 0.04249 |
| *Ppfia2* | protein tyrosine phosphatase_ receptor type_ f polypeptide PTPRF_ interacting protein liprin_ alpha 2 | 1.44 | 0.0494 |
| *Ppfia3* | protein tyrosine phosphatase_ receptor type_ f polypeptide PTPRF_ interacting protein liprin_ alpha 3 | 0.62 | 0.04948 |
| *Ppfia4* | protein tyrosine phosphatase_ receptor type_ f polypeptide PTPRF_ interacting protein liprin_ alpha 4 | 0.67 | 0.04714 |
| *Ppfibp2* | PTPRF interacting protein_ binding protein 2 liprin beta 2 | 1.7 | 0.04059 |
| *Ppif* | peptidylprolyl isomerase F cyclophilin F | 1.24 | 0.00357 |
| *Ppm1b* | protein phosphatase 1B_ magnesium dependent_ beta isoform | 1.39 | 0.00241 |
| *Ppp1r9a* | protein phosphatase 1_ regulatory inhibitor subunit 9A | 1.66 | 0.02646 |
| *Ppp1r9b* | protein phosphatase 1_ regulatory subunit 9B | 0.75 | 0.01575 |
| *Ppp2r2b* | protein phosphatase 2 formerly 2A_ regulatory subunit B PR 52_ beta isoform | 1.38 | 0.04303 |
| *Ppp2r5b* | protein phosphatase 2_ regulatory subunit B'_ beta isoform | 0.56 | 0.02419 |
| *Ppp2r5d* | protein phosphatase 2_ regulatory subunit B'_ delta isoform | 0.71 | 0.01128 |
| *Ppp4r1* | protein phosphatase 4_ regulatory subunit 1 | 0.87 | 0.03985 |
| *Ppp5c* | protein phosphatase 5_ catalytic subunit | 0.65 | 0.01317 |
| *Ppt2* | palmitoyl protein thioesterase 2 | 1.35 | 0.0458 |
| *Prg1* | serglycin | 2.11 | 0.00515 |
| *Prkaa2* | protein kinase_ AMP activated_ alpha 2 catalytic subunit | 0.71 | 0.00846 |
| *Prkab1* | protein kinase_ AMP activated_ beta 1 non catalytic subunit | 1.41 | 0.03216 |
| *Prkacb* | protein kinase_ cAMP dependent_ catalytic_ beta | 1.59 | 0.00499 |
| *Prkcb1* | protein kinase C, beta | 1.49 | 0.0108 |
| *Prkcdbp* | protein kinase C_ delta binding protein | 1.41 | 0.03158 |
| *Prkce* | protein kinase C_ epsilon | 1.49 | 0.0435 |
| *Prkdc* | protein kinase_ DNA activated_ catalytic polypeptide | 0.79 | 0.01568 |
| *Prl* | prolactin | 1.97 | 0.00056 |
| *Prmt5* | protein arginine methyltransferase 5 | 0.74 | 0.01206 |
| *Prnp* | prion protein | 1.21 | 0.04838 |
| *Prosc* | proline synthetase co transcribed homolog bacterial | 1.31 | 0.04302 |
| *Prpf4b* | PRP4 pre mRNA processing factor 4 homolog B yeast | 1.56 | 0.0169 |
| *Prrx1* | paired related homeobox 1 | 0.7 | 0.00992 |
| *Pscd2* | cytohesin 2 | 0.7 | 0.00565 |
| *Psma3* | proteasome prosome_ macropain subunit_ alpha type 3 | 0.91 | 0.04231 |
| *Psma4* | proteasome prosome_ macropain subunit_ alpha type 4 | 0.89 | 0.00296 |
| *Psma6* | proteasome prosome_ macropain subunit_ alpha type 6 | 0.81 | 0.01306 |
| *Psmb10* | proteasome prosome_ macropain subunit_ beta type 10 | 1.43 | 0.02702 |
| *Psmb2* | proteasome prosome_ macropain subunit_ beta type 2 | 0.71 | 0.00013 |
| *Psmb3* | proteasome prosome_ macropain subunit_ beta type 3 | 0.78 | 0.03061 |
| *Psmc2* | proteasome prosome_ macropain 26S subunit_ ATPase 2 | 0.77 | 0.01886 |
| *Psmc4* | proteasome prosome_ macropain 26S subunit_ ATPase_ 4 | 0.77 | 0.00805 |
| *Psmc5* | proteasome prosome_ macropain 26S subunit_ ATPase_ 5 | 0.92 | 0.00126 |
| *Psmd1* | proteasome prosome_ macropain 26S subunit_ non ATPase_ 1 | 0.81 | 1.00E-05 |
| *Psmd4* | proteasome prosome_ macropain 26S subunit_ non ATPase_ 4 | 0.75 | 0.0086 |
| *Psmd7* | proteasome prosome_ macropain 26S subunit_ non ATPase_ 7 | 0.79 | 0.00241 |
| *Ptdsr* | jumonji domain containing 6 | 0.64 | 0.03383 |
| *Pthlh* | parathyroid hormone like hormone | 2.67 | 0.00462 |
| *Ptk2* | PTK2 protein tyrosine kinase 2 | 0.7 | 0.03129 |
| *Ptk2b* | PTK2B protein tyrosine kinase 2 beta | 0.47 | 0.01176 |
| *Ptk9* | twinfilin, actin-binding protein, homolog 1 (Drosophila) | 0.67 | 0.01449 |
| *Ptk9l* | twinfilin, actin-binding protein, homolog 2 (Drosophila) | 0.71 | 0.01968 |
| *Ptov1* | prostate tumor overexpressed 1 | 0.87 | 0.00467 |
| *Ptp4a1* | protein tyrosine phosphatase 4a1 | 0.71 | 0.02888 |
| *Ptpn1* | protein tyrosine phosphatase_ non receptor type 1 | 0.54 | 0.00539 |
| *Ptpn11* | protein tyrosine phosphatase_ non receptor type 11 | 0.67 | 0.03647 |
| *Ptpn2* | protein tyrosine phosphatase_ non receptor type 2 | 0.76 | 0.00392 |
| *Ptpn21* | protein tyrosine phosphatase_ non receptor type 21 | 1.55 | 0.04871 |
| *Ptpn4* | protein tyrosine phosphatase_ non receptor type 4 | 0.55 | 0.04153 |
| *Ptpn7* | protein tyrosine phosphatase_ non receptor type 7 | 1.77 | 0.01126 |
| *Ptprb* | protein tyrosine phosphatase_ receptor type_ B | 0.75 | 0.04778 |
| *Ptprk* | protein tyrosine phosphatase_ receptor type_ K_ extracellular region | 1.3 | 0.03132 |
| *Ptprr* | protein tyrosine phosphatase_ receptor type_ R | 1.47 | 0.01332 |
| *Pycard* | PYD and CARD domain containing | 0.4 | 0.01398 |
| *Pycr2* | pyrroline 5 carboxylate reductase family_ member 2 | 0.7 | 0.01339 |
| *Qprt* | quinolinate phosphoribosyltransferase | 1.58 | 0.04049 |
| *Rab10* | RAB10_ member RAS oncogene family | 0.81 | 0.01467 |
| *Rab11a* | RAB11a_ member RAS oncogene family | 0.85 | 0.00217 |
| *Rab23* | RAB23_ member RAS oncogene family | 0.51 | 0.02804 |
| *Rab27b* | RAB27B_ member RAS oncogene family | 0.36 | 0.01128 |
| *Rab2l* | ral guanine nucleotide dissociation stimulator-like 2 | 0.63 | 0.0048 |
| *Rab31* | RAB31_ member RAS oncogene family | 1.21 | 0.03219 |
| *Rab33a* | RAB33A_ member of RAS oncogene family | 0.72 | 0.04371 |
| *Rab3gap2* | RAB3 GTPase activating protein subunit 2 | 0.86 | 0.0148 |
| *Rab4b* | RAB4B_ member RAS oncogene family | 0.76 | 0.01194 |
| *Rab7* | RAB7, member RAS oncogene family | 1.15 | 0.03113 |
| *Rab9* | RAB9, member RAS oncogene family | 1.18 | 0.0423 |
| *Rabep1* | rabaptin_ RAB GTPase binding effector protein 1 | 0.74 | 0.03273 |
| *Rac1* | ras related C3 botulinum toxin substrate 1 | 0.84 | 0.04889 |
| *Rad17* | RAD17 homolog S. pombe | 0.48 | 0.00083 |
| *Rad18* | RAD18 homolog S. cerevisiae | 0.58 | 0.02294 |
| *Rad54l2* | Rad54 like 2 S. cerevisiae | 0.84 | 0.01674 |
| *Rai1* | retinoic acid induced 1 | 0.78 | 0.00699 |
| *Ralgds* | ral guanine nucleotide dissociation stimulator | 0.66 | 0.0102 |
| *Ralgps2* | Ral GEF with PH domain and SH3 binding motif 2 | 0.68 | 0.03432 |
| *Raly* | RNA binding protein_ autoantigenic hnRNP associated with lethal yellow homolog mouse | 0.86 | 0.04812 |
| *Ramp2* | receptor G protein coupled activity modifying protein 2 | 0.78 | 0.01968 |
| *Rarb* | retinoic acid receptor_ beta | 0.69 | 0.00768 |
| *Rasd2* | RASD family_ member 2 | 1.47 | 0.00785 |
| *Rasgef1a* | RasGEF domain family_ member 1A | 0.38 | 0.01948 |
| *Rasip1* | Ras interacting protein 1 | 0.68 | 0.03595 |
| *Rbbp6* | retinoblastoma binding protein 6 | 0.59 | 0.03175 |
| *Rbl2* | retinoblastoma like 2 | 1.26 | 0.03586 |
| *Rbm10* | RNA binding motif protein 10 | 0.82 | 0.03269 |
| *Rbm13* | MAK16 homolog (S. cerevisiae) | 0.48 | 0.00116 |
| *Rbm25* | RNA binding motif protein 25 | 0.77 | 0.00125 |
| *Rbm27* | RNA binding motif protein 27 | 0.75 | 0.01284 |
| *Rbm5* | RNA binding motif protein 5 | 0.84 | 0.04534 |
| *Rbp1* | retinol binding protein 1_ cellular | 1.41 | 0.03232 |
| *Rbp4* | retinol binding protein 4_ plasma | 2.54 | 0.00197 |
| *Rchy1* | ring finger and CHY zinc finger domain containing 1 | 1.4 | 0.00403 |
| *Rcn1* | reticulocalbin 1_ EF hand calcium binding domain | 1.51 | 0.02821 |
| *Rcor1* | REST corepressor 1 | 0.76 | 0.01041 |
| *Rda279* | SET domain containing 4 | 0.87 | 0.00173 |
| *Recql* | RecQ protein like DNA helicase Q1 like | 0.71 | 0.00783 |
| *Rent1* | UPF1 regulator of nonsense transcripts homolog (yeast) | 0.6 | 0.0186 |
| *Rev1l* | REV1 homolog (S. cerevisiae) | 0.66 | 0.00598 |
| *Rg9mtd3* | RNA guanine 9 methyltransferase domain containing 3 | 1.45 | 0.02031 |
| *Rgs19* | regulator of G protein signaling 19 | 0.68 | 0.0308 |
| *Rgs2* | regulator of G protein signaling 2 | 3.4 | 0.01426 |
| *Rgs4* | regulator of G protein signaling 4 | 1.87 | 0.00547 |
| *Rhebl1* | Ras homolog enriched in brain like 1 | 0.77 | 0.03346 |
| *Rhobtb2* | Rho related BTB domain containing 2 | 0.76 | 0.04471 |
| *Rhoq* | ras homolog gene family_ member Q | 0.61 | 0.04682 |
| *Rhpn1* | rhophilin_ Rho GTPase binding protein 1 | 0.69 | 0.00162 |
| *Rims2* | regulating synaptic membrane exocytosis 2 | 0.52 | 0.02743 |
| *Rit2* | Ras like without CAAX 2 | 1.41 | 0.02459 |
| *Rkhd1* | ring finger C3HC4 type and KH domain containing 1 | 1.45 | 0.00528 |
| *Rnaset2* | ribonuclease T2 | 1.33 | 0.04867 |
| *Rnf111* | ring finger protein 111 | 0.78 | 0.01778 |
| *Rnf126* | ring finger protein 126 | 0.7 | 0.04875 |
| *Rnf138* | ring finger protein 138 | 0.79 | 0.04091 |
| *Rnf14* | ring finger protein 14 | 0.96 | 0.00165 |
| *Rnf19* | ring finger protein 19A | 0.74 | 0.03987 |
| *Rnf25* | ring finger protein 25 | 0.82 | 0.03358 |
| *Rnf7* | ring finger protein 7 | 1.23 | 0.04616 |
| *Rnf8* | ring finger protein 8 | 0.83 | 0.02143 |
| *Rnmt* | RNA guanine 7 methyltransferase | 0.82 | 0.02115 |
| *Rock2* | Rho associated coiled coil containing protein kinase 2 | 0.53 | 0.00808 |
| *Rorb* | RAR related orphan receptor B | 0.64 | 0.02185 |
| *Rpa3* | replication protein A3 | 1.35 | 0.02403 |
| *Rpl10* | ribosomal protein L10 | 1.01 | 0.02858 |
| *Rpl15* | ribosomal protein L15 | 1.45 | 0.03212 |
| *Rpl35* | ribosomal protein L35 | 1.06 | 0.04383 |
| *Rpl35a* | ribosomal protein L35a | 1.2 | 0.04955 |
| *Rpl37* | ribosomal protein L37 | 1.56 | 1.00E-04 |
| *Rpl39* | ribosomal protein L39 | 1.04 | 0.03594 |
| *Rpn2* | ribophorin II | 0.7 | 0.00377 |
| *Rpo1-1* | polymerase (RNA) I polypeptide C | 0.81 | 0.00057 |
| *Rps15* | ribosomal protein S15 | 2.18 | 0.03439 |
| *Rps26* | ribosomal protein S26 | 1.25 | 0.03892 |
| *Rps5* | ribosomal protein S5 | 3.06 | 0.00963 |
| *Rpusd4* | RNA pseudouridylate synthase domain containing 4 | 1.46 | 0.04776 |
| *Rrbp1* | ribosome binding protein 1 | 0.78 | 0.03745 |
| *Rsbn1l* | round spermatid basic protein 1 like | 0.6 | 0.02025 |
| *Rsrc1* | arginine/serine rich coiled coil 1 | 1.57 | 0.01017 |
| *Rtn4r* | reticulon 4 receptor | 0.77 | 0.03916 |
| *Rtn4rl2* | reticulon 4 receptor like 2 | 2.14 | 0.00134 |
| *Rutbc1* | small G protein signaling modulator 2 | 0.5 | 0.02134 |
| *Rwdd3* | RWD domain containing 3 | 1.54 | 0.01172 |
| *Rxrg* | retinoid X receptor gamma | 2.59 | 0.00556 |
| *S100a1* | S100 calcium binding protein A1 | 1.82 | 0.00654 |
| *S100a10* | S100 calcium binding protein A10 | 1.53 | 0.03854 |
| *S100a16* | S100 calcium binding protein A16 | 2.3 | 0.04577 |
| *S100a4* | S100 calcium binding protein A4 | 1.71 | 0.03528 |
| *S63167* |  | 2.34 | 0.00489 |
| *S63519* |  | 1.49 | 0.01981 |
| *Sall1* | sal like 1 Drosophila | 0.61 | 0.00419 |
| *Sall2* | sal like 2 Drosophila | 2.93 | 0.01454 |
| *Sall3* | sal like 3 Drosophila | 1.48 | 0.00019 |
| *Sar1b* | SAR1 homolog B S. cerevisiae | 1.36 | 0.00233 |
| *Sbf1* | SET binding factor 1 | 0.63 | 0.00402 |
| *Sc4mol* | sterol C4 methyl oxidase like | 0.56 | 0.01371 |
| *Sc5d* | sterol-C5-desaturase (fungal ERG3, delta-5-desaturase) homolog (S. cerevisae) | 0.41 | 0.00863 |
| *Scamp1* | secretory carrier membrane protein 1 | 1.25 | 0.01731 |
| *Scand1* | SCAN domain containing 1 | 1.29 | 0.0264 |
| *Scap* | SREBF chaperone | 0.74 | 0.00641 |
| *Scarf2* | scavenger receptor class F_ member 2 | 1.97 | 0.01207 |
| *Scg3* | secretogranin III | 1.26 | 0.00787 |
| *Scn8a* | sodium channel_ voltage gated_ type VIII_ alpha | 0.57 | 0.03828 |
| *Scnm1* | sodium channel modifier 1 | 1.6 | 0.04138 |
| *Scnn1a* | sodium channel_ nonvoltage gated_ type I_ alpha | 1.69 | 0.03712 |
| *Scrg1* | stimulator of chondrogenesis 1 | 2.09 | 0.00361 |
| *Scube1* | signal peptide_ CUB domain_ EGF like 1 | 0.56 | 0.04936 |
| *Scye1* | aminoacyl tRNA synthetase complex-interacting multifunctional protein 1 | 1.6 | 0.0131 |
| *Scyl2* | SCY1 like 2 S. cerevisiae | 0.65 | 0.00859 |
| *Sdc2* | syndecan 2 | 0.73 | 0.04346 |
| *Sdccag3* | serologically defined colon cancer antigen 3 | 0.64 | 0.00489 |
| *Sdccag8* | serologically defined colon cancer antigen 8 | 1.55 | 0.0175 |
| *Sdhc* | succinate dehydrogenase complex_ subunit C_ integral membrane protein | 1.5 | 0.00568 |
| *Sec11l3* | SEC11 homolog C (S. cerevisiae) | 0.68 | 0.02384 |
| *Sec23ip* | SEC23 interacting protein | 0.63 | 0.00169 |
| *Sec24b* | SEC24 family_ member B S. cerevisiae | 0.74 | 0.01383 |
| *Sec61b* | Sec61 beta subunit | 1.27 | 0.00661 |
| *Sec61g* | SEC61_ gamma subunit | 1.27 | 0.04254 |
| *Sema3b* | sema domain, immunoglobulin domain (Ig), short basic domain, secreted, (semaphorin) 3B | 1.93 | 0.04667 |
| *Sema3e* | sema domain_ immunoglobulin domain Ig_ short basic domain_ secreted_ semaphorin 3E | 1.45 | 0.03059 |
| *Sema6a* | sema domain_ transmembrane domain TM_ and cytoplasmic domain_ semaphorin 6A | 1.22 | 0.04629 |
| *Sema6c* | sema domain_ transmembrane domain TM_ and cytoplasmic domain_ semaphorin 6C | 0.77 | 0.02443 |
| *Sema6d* | sema domain_ transmembrane domain TM_ and cytoplasmic domain_ semaphorin 6D | 1.21 | 0.01439 |
| *Senp5* | Sumo1/sentrin/SMT3 specific peptidase 5 | 0.77 | 0.04962 |
| *Senp6* | Sumo1/sentrin/SMT3 specific peptidase 6 | 0.66 | 0.00038 |
| *Serpinb5* | serine or cysteine peptidase inhibitor_ clade B_ member 5 | 0.77 | 0.02139 |
| *Set* | SET nuclear oncogene | 1.36 | 0.02 |
| *Sf3b2* | splicing factor 3b_ subunit 2 | 1.32 | 0.01382 |
| *Sfrs15* | splicing factor_ arginine/serine rich 15 | 0.71 | 0.03543 |
| *Sfrs2* | splicing factor_ arginine/serine rich 2 | 0.66 | 0.0442 |
| *Sfrs5* | splicing factor_ arginine/serine rich 5 | 1.41 | 9.00E-05 |
| *Sfrs9* | splicing factor_ arginine/serine rich 9 | 0.75 | 0.01433 |
| *Sfxn4* | sideroflexin 4 | 0.87 | 0.01819 |
| *Sgol2* | shugoshin like 2 S. pombe | 1.93 | 0.03395 |
| *Sgpp1* | sphingosine 1 phosphate phosphatase 1 | 0.74 | 0.00589 |
| *Sgta* | small glutamine rich tetratricopeptide repeat TPR containing_ alpha | 0.84 | 0.00516 |
| *Sh2bpsm1* | SH2 B PH domain containing signaling mediator 1 | 0.66 | 0.00169 |
| *Sh3bgrl* | SH3 domain binding glutamic acid rich protein like | 1.99 | 0.00268 |
| *Sh3bp5* | SH3 domain binding protein 5 BTK associated | 1.13 | 0.04279 |
| *Sh3glb1* | SH3 domain GRB2 like endophilin B1 | 1.47 | 0.00648 |
| *Shank1* | SH3 and multiple ankyrin repeat domains 1 | 0.78 | 0.04836 |
| *Shank3* | SH3 and multiple ankyrin repeat domains 3 | 0.71 | 0.03246 |
| *Shkbp1* | Sh3kbp1 binding protein 1 | 0.53 | 0.00534 |
| *Shprh* | SNF2 histone linker PHD RING helicase | 0.65 | 0.00023 |
| *Sirt3* | sirtuin silent mating type information regulation 2 homolog 3 S. cerevisiae | 1.37 | 0.00114 |
| *Sirt5* | sirtuin silent mating type information regulation 2 homolog 5 S. cerevisiae | 1.38 | 0.02722 |
| *Sirt7* | sirtuin 7 silent mating type information regulation 2_ homolog 7 S. cerevisiae | 0.57 | 0.00432 |
| *Skp1a* | S-phase kinase-associated protein 1A | 1.01 | 0.0416 |
| *Slb* | intraflagellar transport 172 | 0.88 | 0.02197 |
| *Slc11a2* | solute carrier family 11 proton coupled divalent metal ion transporters_ member 2 | 0.63 | 0.00472 |
| *Slc12a2* | solute carrier family 12 sodium/potassium/chloride transporters_ member 2 | 0.47 | 0.00028 |
| *Slc12a7* | solute carrier family 12 potassium/chloride transporters_ member 7 | 1.24 | 0.04714 |
| *Slc13a3* | solute carrier family 13 sodium dependent dicarboxylate transporter_ member 3 | 2.56 | 0.00434 |
| *Slc16a6* | solute carrier family 16_ member 6 monocarboxylic acid transporter 7 | 1.25 | 0.04464 |
| *Slc17a6* | solute carrier family 17 sodium dependent inorganic phosphate cotransporter_ member 6 | 0.78 | 0.01626 |
| *Slc18a3* | solute carrier family 18 vesicular acetylcholine_ member 3 | 0.49 | 0.02494 |
| *Slc1a1* | solute carrier family 1 neuronal/epithelial high affinity glutamate transporter_ system Xag_ member 1 | 0.66 | 0.0127 |
| *Slc1a2* | solute carrier family 1 glial high affinity glutamate transporter_ member 2 | 1.72 | 0.01013 |
| *Slc1a3* | solute carrier family 1 glial high affinity glutamate transporter_ member 3 | 0.85 | 0.02541 |
| *Slc1a5* | solute carrier family 1 neutral amino acid transporter_ member 5 | 0.23 | 0.02263 |
| *Slc20a1* | solute carrier family 20 phosphate transporter_ member 1 | 0.41 | 0.00649 |
| *Slc25a1* | solute carrier family 25 mitochondrial carrier_ citrate transporter_ member 1 | 0.57 | 0.0161 |
| *Slc25a4* | solute carrier family 25 mitochondrial carrier; adenine nucleotide translocator_ member 4 | 1.14 | 0.00937 |
| *Slc25a5* | solute carrier family 25 mitochondrial carrier; adenine nucleotide translocator_ member 5 | 0.73 | 0.01251 |
| *Slc29a3* | solute carrier family 29 nucleoside transporters_ member 3 | 0.65 | 0.00496 |
| *Slc2a1* | solute carrier family 2 facilitated glucose transporter_ member 1 | 0.68 | 0.04363 |
| *Slc2a4* | solute carrier family 2 facilitated glucose transporter_ member 4 | 1.74 | 0.0299 |
| *Slc2a6* | solute carrier family 2 facilitated glucose transporter_ member 6 | 0.47 | 0.04777 |
| *Slc2a8* | solute carrier family 2_ facilitated glucose transporter member 8 | 0.7 | 0.02048 |
| *Slc30a1* | solute carrier family 30 zinc transporter_ member 1 | 0.62 | 0.04454 |
| *Slc30a6* | solute carrier family 30 zinc transporter_ member 6 | 0.74 | 0.01393 |
| *Slc35b1* | solute carrier family 35_ member B1 | 0.52 | 0.00164 |
| *Slc35e4* | solute carrier family 35_ member E4 | 0.6 | 0.02119 |
| *Slc38a2* | solute carrier family 38_ member 2 | 0.67 | 0.01029 |
| *Slc39a14* | solute carrier family 39 zinc transporter_ member 14 | 0.54 | 0.02342 |
| *Slc4a3* | solute carrier family 4 anion exchanger_ member 3 | 0.73 | 0.0347 |
| *Slc4a4* | solute carrier family 4 anion exchanger_ member 4 | 2.62 | 0.00222 |
| *Slc5a7* | solute carrier family 5 choline transporter_ member 7 | 2.05 | 0.01573 |
| *Slc7a1* | solute carrier family 7 cationic amino acid transporter_ y+ system_ member 1 | 1.25 | 0.04485 |
| *Slc7a10* | solute carrier family 7_ neutral amino acid transporter_ y+ system member 10 | 1.52 | 0.03756 |
| *Slc7a13* | solute carrier family 7_ cationic amino acid transporter_ y+ system member 13 | 0.31 | 0.02813 |
| *Slc7a4* | solute carrier family 7 cationic amino acid transporter_ y+ system_ member 4 | 0.42 | 0.00045 |
| *Slc7a8* | solute carrier family 7 cationic amino acid transporter_ y+ system_ member 8 | 1.43 | 0.02861 |
| *Slc9a5* | solute carrier family 9 sodium/hydrogen exchanger_ member 5 | 0.38 | 0.01376 |
| *Slco3a1* | solute carrier organic anion transporter family_ member 3a1 | 0.62 | 0.04428 |
| *Slit1* | slit homolog 1 Drosophila | 0.59 | 0.04599 |
| *Slit2* | slit homolog 2 Drosophila | 1.36 | 0.0216 |
| *Slitrk5* | SLIT and NTRK like family_ member 5 | 1.22 | 0.04854 |
| *Slmap* | sarcolemma associated protein | 0.68 | 0.012 |
| *Smad2* | SMAD family member 2 | 0.62 | 0.01402 |
| *Smarca3* | helicase-like transcription factor | 1.52 | 0.00075 |
| *Smarca4* | SWI/SNF related_ matrix associated_ actin dependent regulator of chromatin_ subfamily a_ member 4 | 0.82 | 0.00099 |
| *Smarcal1* | Swi/SNF related matrix associated_ actin dependent regulator of chromatin_ subfamily a like 1 | 0.74 | 0.04514 |
| *Smc5l1* | structural maintenance of chromosomes 5 | 0.61 | 0.04854 |
| *Snai2* | snail homolog 2 Drosophila | 1.52 | 0.04526 |
| *Snap29* | synaptosomal associated protein 29 | 0.51 | 0.02085 |
| *Snca* | synuclein_ alpha non A4 component of amyloid precursor | 0.67 | 0.03349 |
| *Sned1* | sushi_ nidogen and EGF like domains 1 | 1.94 | 0.01545 |
| *Snf1lk* | SNF1 like kinase | 0.59 | 0.02454 |
| *Snrp1c* | U1 small nuclear ribonucleoprotein C | 1.5 | 0.00575 |
| *Snrp70* | small nuclear ribonucleoprotein 70 (U1) | 0.71 | 0.01112 |
| *Snrpd1* | small nuclear ribonucleoprotein D1 | 1.11 | 0.04848 |
| *Snx10* | sorting nexin 10 | 1.42 | 0.01645 |
| *Snx24* | sorting nexin 24 | 1.35 | 0.00611 |
| *Snx25* | sorting nexin 25 | 0.55 | 0.01374 |
| *Snx7* | sorting nexin 7 | 1.64 | 0.00434 |
| *Sod3* | superoxide dismutase 3_ extracellular | 0.38 | 0.0423 |
| *Son* | Son DNA binding protein | 0.83 | 0.0194 |
| *Sorcs1* | sortilin related VPS10 domain containing receptor 1 | 0.58 | 0.01659 |
| *Sorcs3* | sortilin related VPS10 domain containing receptor 3 | 2.18 | 0.00095 |
| *Sox10* | SRY sex determining region Y box 10 | 2.81 | 0.01143 |
| *Sox5* | SRY box containing gene 5 | 0.75 | 0.04588 |
| *Spast* | spastin | 0.82 | 0.0378 |
| *Spata5* | spermatogenesis associated 5 | 0.4 | 0.01612 |
| *Spg20* | spastic paraplegia 20 Troyer syndrome homolog human | 1.78 | 0.00616 |
| *Spg21* | spastic paraplegia 21 homolog human | 0.77 | 0.04867 |
| *Spire1* | spire homolog 1 Drosophila | 0.74 | 0.01936 |
| *Spnb4* | spectrin beta 4 | 0.47 | 0.01621 |
| *Spock1* | sparc/osteonectin_ cwcv and kazal like domains proteoglycan testican 1 | 0.69 | 0.00913 |
| *Spry1* | sprouty homolog 1_ antagonist of FGF signaling Drosophila | 0.59 | 0.0088 |
| *Spsb4* | splA/ryanodine receptor domain and SOCS box containing 4 | 1.5 | 0.01034 |
| *Sqle* | squalene epoxidase | 0.39 | 0.018 |
| *Sqstm1* | sequestosome 1 | 0.69 | 0.04348 |
| *Src* | v src sarcoma Schmidt Ruppin A 2 viral oncogene homolog avian | 0.79 | 0.00983 |
| *Srfbp1* | serum response factor binding protein 1 | 0.6 | 0.00622 |
| *Srm* | spermidine synthase | 0.78 | 0.02676 |
| *Srprb* | signal recognition particle receptor_ B subunit | 0.88 | 0.03786 |
| *Srrm2* | serine/arginine repetitive matrix 2 | 0.78 | 0.0108 |
| *Ssr4* | signal sequence receptor_ delta | 0.87 | 0.01013 |
| *Ssx2ip* | synovial sarcoma_ X breakpoint 2 interacting protein | 0.63 | 0.03197 |
| *St3gal5* | ST3 beta galactoside alpha 2_3 sialyltransferase 5 | 0.8 | 0.00307 |
| *ST7* | suppression of tumorigenicity 7 | 0.65 | 0.00847 |
| *Stag2* | stromal antigen 2 | 1.45 | 0.00043 |
| *Stat3* | signal transducer and activator of transcription 3 | 0.72 | 0.02887 |
| *Stk38* | serine/threonine kinase 38 | 0.56 | 8.00E-05 |
| *Stk39* | serine/threonine kinase 39_ STE20/SPS1 homolog yeast | 0.7 | 0.01741 |
| *Ston2* | stonin 2 | 1.58 | 0.03156 |
| *Stra6* | stimulated by retinoic acid gene 6 | 0.32 | 0.01622 |
| *Strn3* | striatin_ calmodulin binding protein 3 | 0.74 | 0.02557 |
| *Stx12* | syntaxin 12 | 1.33 | 0.01552 |
| *Stx1a* | syntaxin 1A brain | 0.73 | 0.02463 |
| *Stxbp5l* | syntaxin binding protein 5 like | 0.58 | 0.0323 |
| *Stxbp6* | syntaxin binding protein 6 (amisyn) | 2.32 | 0.00099 |
| *Surb7* | mediator complex subunit 21 | 1.39 | 0.01764 |
| *Surf4* | surfeit 4 | 1.31 | 0.023 |
| *Syncrip* | synaptotagmin binding_ cytoplasmic RNA interacting protein | 0.74 | 0.04194 |
| *Syngr1* | synaptogyrin 1 | 1.31 | 0.02313 |
| *Syt3* | synaptotagmin III | 0.55 | 0.01415 |
| *Syvn1* | synovial apoptosis inhibitor 1_ synoviolin | 0.59 | 0.00363 |
| *Tac1* | tachykinin 1 | 3.54 | 0.03497 |
| *Tac4* | tachykinin 4 | 0.38 | 0.02538 |
| *Taf1* | TAF1 RNA polymerase II_ TATA box binding protein TBP associated factor | 1.93 | 0.03675 |
| *Taf9* | TAF9 RNA polymerase II_ TATA box binding protein TBP associated factor | 0.53 | 0.01947 |
| *Taldo1* | transaldolase 1 | 1.24 | 0.00183 |
| *Tank* | TRAF family member associated NFKB activator | 0.71 | 0.01412 |
| *Tap2* | transporter 2_ ATP binding cassette_ sub family B MDR/TAP | 0.61 | 0.0114 |
| *Tasp1* | taspase_ threonine aspartase 1 | 1.18 | 0.02141 |
| *Tax1bp3* | Tax1 human T cell leukemia virus type I binding protein 3 | 1.62 | 0.00804 |
| *Tbc1d10b* | TBC1 domain family, member 10b | 0.79 | 0.02893 |
| *Tbc1d19* | TBC1 domain family_ member 19 | 0.73 | 0.02865 |
| *Tbc1d20* | TBC1 domain family_ member 20 | 1.92 | 0.03745 |
| *Tbcc* | tubulin folding cofactor C | 0.72 | 0.00368 |
| *Tbce* | tubulin folding cofactor E | 0.82 | 0.02004 |
| *Tbl3* | transducin beta like 3 | 0.76 | 0.02128 |
| *Tbrg4* | transforming growth factor beta regulator 4 | 0.76 | 0.01557 |
| *Tbx4* | T box 4 | 0.68 | 0.00529 |
| *Tctex1* | dynein light chain Tctex-type 1B | 1.17 | 0.02659 |
| *Tdg* | thymine DNA glycosylase | 0.72 | 0.00602 |
| *Tdrd3* | tudor domain containing 3 | 1.37 | 0.01687 |
| *Tdrd7* | tudor domain containing 7 | 0.71 | 0.0368 |
| *Terf2ip* | telomeric repeat binding factor 2_ interacting protein | 0.68 | 0.00282 |
| *Tesk1* | testis specific kinase 1 | 0.64 | 5.00E-05 |
| *Tex264* | testis expressed 264 | 0.76 | 0.01248 |
| *Tfg* | Trk fused gene | 0.78 | 0.02755 |
| *Tfrc* | transferrin receptor | 0.73 | 0.02099 |
| *Thap11* | THAP domain containing 11 | 0.87 | 0.03507 |
| *Thbs4* | thrombospondin 4 | 0.11 | 0 |
| *Thex1* | three prime histone mRNA exonuclease 1 | 0.58 | 0.03368 |
| *Thop1* | thimet oligopeptidase 1 | 0.73 | 0.00828 |
| *Thrap1* | mediator complex subunit 13 | 0.5 | 0.02148 |
| *Thrap2* | mediator complex subunit 13-like | 0.71 | 0.03788 |
| *Thrap5* | mediator complex subunit 16 | 0.89 | 0.04204 |
| *Thumpd1* | THUMP domain containing 1 | 1.45 | 0.0189 |
| *Tiam1* | T cell lymphoma invasion and metastasis 1 | 0.69 | 0.03848 |
| *Timm23* | translocase of inner mitochondrial membrane 23 homolog yeast | 0.81 | 0.00917 |
| *Timm8b* | translocase of inner mitochondrial membrane 8 homolog b yeast | 1.27 | 0.01189 |
| *Tiparp* | TCDD inducible polyADP ribose polymerase | 0.71 | 0.02302 |
| *Tiprl* | TIP41_ TOR signaling pathway regulator like S. cerevisiae | 0.82 | 0.02347 |
| *Tjp1* | tight junction protein 1 | 0.72 | 0.01452 |
| *Tk1* | thymidine kinase 1_ soluble | 0.65 | 0.03255 |
| *Tk2* | thymidine kinase 2_ mitochondrial | 1.32 | 0.00918 |
| *Tkt* | transketolase | 0.99 | 0.01086 |
| *Tlcd1* | TLC domain containing 1 | 0.5 | 0.01422 |
| *Tle2* | transducin like enhancer of split 2 Esp1 homolog_ Drosophila | 0.57 | 0.00018 |
| *Tle3* | transducin like enhancer of split 3 Esp1 homolog_ Drosophila | 0.71 | 0.01959 |
| *Tm4sf12* | tetraspanin 12 | 0.59 | 0.03166 |
| *Tm7sf2* | transmembrane 7 superfamily member 2 | 0.56 | 0.04019 |
| *Tmbim1* | transmembrane BAX inhibitor motif containing 1 | 0.18 | 0.02109 |
| *Tmed7* | transmembrane emp24 protein transport domain containing 7 | 1.46 | 0.00081 |
| *Tmeff2* | transmembrane protein with EGF like and two follistatin like domains 2 | 1.78 | 0.02697 |
| *Tmem15* | dolichol kinase | 0.87 | 0.01903 |
| *Tmem30a* | transmembrane protein 30A | 0.6 | 0.00612 |
| *Tmem38a* | transmembrane protein 38a | 0.5 | 0.04705 |
| *Tmem58* | shisa homolog 4 (Xenopus laevis) | 1.42 | 0.02074 |
| *Tmem60* | transmembrane protein 60 | 1.19 | 0.03268 |
| *Tmem63b* | transmembrane protein 63b | 0.66 | 0.04437 |
| *Tmem63c* | transmembrane protein 63c | 0.59 | 0.00922 |
| *Tmem8* | transmembrane protein 8 five membrane spanning domains | 0.56 | 0.00439 |
| *Tmem93* | transmembrane protein 93 | 1.25 | 0.0185 |
| *Tmem97* | transmembrane protein 97 | 0.67 | 0.01249 |
| *Tnni3* | troponin I type 3 cardiac | 0.71 | 0.03868 |
| *Tomm40* | translocase of outer mitochondrial membrane 40 homolog yeast | 0.56 | 0.01408 |
| *Tp53* | tumor protein p53 | 0.72 | 0.00891 |
| *Tparl* | transmembrane protein 165 | 0.76 | 0.0287 |
| *Tpbg* | trophoblast glycoprotein | 0.73 | 0.03579 |
| *Tpm3* | tropomyosin 3_ gamma | 1.6 | 0.00264 |
| *Tpo1* | developmentally regulated protein TPO1 | 1.55 | 0.01659 |
| *Tprkb* | Tp53rk binding protein | 1.5 | 0.00707 |
| *Tra1* | tumor rejection antigen gp96 | 0.51 | 0.02257 |
| *Traf3* | Tnf receptor associated factor 3 | 0.7 | 0.04928 |
| *Trak2* | trafficking protein_ kinesin binding 2 | 0.64 | 0.00315 |
| *Trex1* | three prime repair exonuclease 1 | 0.68 | 0.00889 |
| *Trim37* | tripartite motif containing 37 | 0.77 | 0.02223 |
| *Trim45* | tripartite motif containing 45 | 1.76 | 0.02197 |
| *Trio* | triple functional domain PTPRF interacting | 0.84 | 0.04733 |
| *Trmt12* | tRNA methyltransferase 12 homolog S. cerevisiae | 1.24 | 0.01286 |
| *Trove2* | TROVE domain family_ member 2 | 0.47 | 0.00103 |
| *Trp53inp1* | transformation related protein 53 inducible nuclear protein 1 | 0.53 | 0.02839 |
| *Trpc1* | transient receptor potential cation channel_ subfamily C_ member 1 | 1.3 | 0.03453 |
| *Trpc4ap* | transient receptor potential cation channel_ subfamily C_ member 4 associated protein | 0.74 | 0.03475 |
| *Trps1* | trichorhinophalangeal syndrome I homolog human | 1.44 | 0.0085 |
| *Tsen2* | tRNA splicing endonuclease 2 homolog S. cerevisiae | 0.78 | 0.00768 |
| *Tspan1* | tetraspanin 1 | 2.36 | 0.00197 |
| *Tspan18* | tetraspanin 18 | 0.64 | 0.03649 |
| *Ttbk1* | tau tubulin kinase 1 | 1.38 | 0.00867 |
| *Ttc3* | tetratricopeptide repeat domain 3 | 1.27 | 0.03856 |
| *Ttc6* | tetratricopeptide repeat domain 6 | 2.06 | 0.04696 |
| *Ttc8* | tetratricopeptide repeat domain 8 | 1.46 | 0.03295 |
| *Ttc9c* | tetratricopeptide repeat domain 9C | 0.81 | 0.02892 |
| *Tubb2b* | tubulin_ beta 2b | 1.01 | 0.01338 |
| *Tubb2c* | tubulin_ beta 2c | 0.76 | 0 |
| *Tubb4* | tubulin_ beta 4 | 0.8 | 0.042 |
| *Txndc11* | thioredoxin domain containing 11 | 0.6 | 0.03279 |
| *Txnip* | thioredoxin interacting protein | 3.71 | 0.0057 |
| *Txnrd1* | thioredoxin reductase 1 | 0.7 | 0.0462 |
| *U02094* |  | 0.55 | 0.02744 |
| *U68562* |  | 1.34 | 0.01523 |
| *Uap1l1* | UDP N acteylglucosamine pyrophosphorylase 1 like 1 | 2.12 | 0.0074 |
| *Ubap1* | ubiquitin associated protein 1 | 0.91 | 0.0083 |
| *Ubap2* | ubiquitin associated protein 2 | 0.75 | 0.03256 |
| *Ubb* | ubiquitin B | 1.08 | 0.04247 |
| *Ubc* | ubiquitin C | 1.06 | 0.03426 |
| *Ube1dc1* | ubiquitin-like modifier activating enzyme 5 | 0.75 | 0.01342 |
| *Ube2a* | ubiquitin conjugating enzyme E2A_ RAD6 homolog S. cerevisiae | 1.27 | 0.01481 |
| *Ube2d2* | ubiquitin conjugating enzyme E2D 2 | 0.8 | 0.0408 |
| *Ube2g1* | ubiquitin conjugating enzyme E2G 1 UBC7 homolog_ C. elegans | 0.46 | 0.04853 |
| *Ube2i* | ubiquitin conjugating enzyme E2I | 0.81 | 0.02066 |
| *Ube2q* | ubiquitin-conjugating enzyme E2Q (putative) 1 | 0.74 | 0.00191 |
| *Ube2v2* | ubiquitin conjugating enzyme E2 variant 2 | 1.17 | 0.00556 |
| *Ube2z* | ubiquitin conjugating enzyme E2Z | 0.55 | 0.00306 |
| *Ube3a* | ubiquitin protein ligase E3A | 1.24 | 0.00373 |
| *Ube4a* | ubiquitination factor E4A | 1.22 | 0.04793 |
| *Ubl4a* | ubiquitin-like 4 | 1.29 | 0.00413 |
| *Ublcp1* | ubiquitin like domain containing CTD phosphatase 1 | 0.53 | 0.04837 |
| *Ubtf* | upstream binding transcription factor_ RNA polymerase I | 0.5 | 0.00411 |
| *Ubxd6* | UBX domain containing 6 | 1.61 | 0.03477 |
| *Uck1* | uridine cytidine kinase 1 | 0.79 | 0.00766 |
| *Ugdh* | UDP glucose dehydrogenase | 0.73 | 0.01528 |
| *Uhmk1* | U2AF homology motif UHM kinase 1 | 0.58 | 0.02694 |
| *Unc45a* | unc 45 homolog A C. elegans | 0.63 | 0.03266 |
| *Unc5c* | unc 5 homolog C C. elegans | 1.8 | 0.04062 |
| *Unc5d* | unc 5 homolog D C. elegans | 1.77 | 0.01337 |
| *Usp10* | ubiquitin specific peptidase 10 | 0.6 | 0.02755 |
| *Usp14* | ubiquitin specific peptidase 14 | 0.73 | 0.03231 |
| *Usp19* | ubiquitin specific peptidase 19 | 0.66 | 0.00364 |
| *Usp25* | ubiquitin specific peptidase 25 | 1.48 | 0.00755 |
| *Usp29* | ubiquitin specific peptidase 29 | 1.61 | 0.00096 |
| *Usp47* | ubiquitin specific peptidase 47 | 0.7 | 0.00064 |
| *Usp48* | ubiquitin specific protease 48 | 0.77 | 0.0046 |
| *Usp9x* | ubiquitin specific peptidase 9_ X linked | 0.79 | 0.04593 |
| *Utp14a* | UTP14_ U3 small nucleolar ribonucleoprotein_ homolog A yeast | 0.64 | 0.01825 |
| *Vac14* | Vac14 homolog S. cerevisiae | 0.28 | 0.00195 |
| *Vamp3* | vesicle associated membrane protein 3 | 0.89 | 0.01096 |
| *Vamp4* | vesicle associated membrane protein 4 | 1.27 | 0.01262 |
| *Vav2* | vav 2 guanine nucleotide exchange factor | 0.8 | 0.01986 |
| *Vcam1* | vascular cell adhesion molecule 1 | 2.16 | 0.0173 |
| *Vil2* | ezrin | 1.27 | 0.04341 |
| *Vkorc1* | vitamin K epoxide reductase complex_ subunit 1 | 1.62 | 0.00669 |
| *Vmp* | neurensin 1 | 1.44 | 0.02711 |
| *Vps36* | vacuolar protein sorting 36 homolog S. cerevisiae | 0.74 | 0.03102 |
| *Vps52* | vacuolar protein sorting 52 homolog S. cerevisiae | 0.65 | 0.00815 |
| *Vsnl1* | visinin like 1 | 1.64 | 0.00876 |
| *Wasl* | Wiskott Aldrich syndrome like | 0.73 | 0.02597 |
| *Wbp11* | WW domain binding protein 11 | 1.3 | 0.03037 |
| *Wbp5* | WW domain binding protein 5 | 0.83 | 0.03971 |
| *Wdfy3* | WD repeat and FYVE domain containing 3 | 1.22 | 0.03698 |
| *Wdr13* | WD repeat domain 13 | 1.31 | 0.03707 |
| *Wdr21* | WD repeat domain 21 | 0.72 | 0.02893 |
| *Wdr24* | WD repeat domain 24 | 0.65 | 0.00134 |
| *Wdr31* | WD repeat domain 31 | 1.56 | 0.02855 |
| *Wdr33* | WD repeat domain 33 | 0.66 | 0.00868 |
| *Wdr34* | WD repeat domain 34 | 0.84 | 0.04223 |
| *Wdr37* | WD repeat domain 37 | 0.78 | 0.01927 |
| *Wdr50* | UTP18, small subunit (SSU) processome component, homolog (yeast) | 0.55 | 0.00098 |
| *Wdr61* | WD repeat domain 61 | 0.82 | 0.00688 |
| *Wdtc1* | WD and tetratricopeptide repeats 1 | 1.22 | 0.0418 |
| *Wee1* | wee 1 homolog S. pombe | 0.77 | 0.02795 |
| *Wfdc2* | WAP four disulfide core domain 2 | 1.66 | 0.01062 |
| *Wfikkn1* | WAP_ FS_ Ig_ KU_ and NTR containing protein 1 | 0.38 | 0.00652 |
| *Whsc1l1* | Wolf Hirschhorn syndrome candidate 1 like 1 human | 0.71 | 0.03829 |
| *Wif1* | Wnt inhibitory factor 1 | 1.44 | 0.03174 |
| *Wnt11* | wingless type MMTV integration site family_ member 11 | 1.62 | 0.02633 |
| *Wnt7a* | wingless type MMTV integration site family_ member 7A | 0.71 | 0.02421 |
| *Wnt7b* | wingless type MMTV integration site family_ member 7B | 0.64 | 0.03628 |
| *Wtip* | Wilms tumor 1 interacting protein | 0.64 | 0.00422 |
| *Xpo1* | exportin 1_ CRM1 homolog yeast | 0.67 | 0.00223 |
| *Xrcc4* | X ray repair complementing defective repair in Chinese hamster cells 4 | 1.63 | 0.03303 |
| *Y17323* |  | 1.09 | 0.04543 |
| *Yipf3* | Yip1 domain family_ member 3 | 0.81 | 0.04 |
| *Ythdf2* | YTH domain family_ member 2 | 1.54 | 0.01428 |
| *Ywhaz* | tyrosine 3 monooxygenase/tryptophan 5 monooxygenase activation protein_ zeta polypeptide | 0.81 | 0.04589 |
| *Yy1* | YY1 transcription factor | 0.74 | 0.00079 |
| *Zbtb1* | zinc finger and BTB domain containing 1 | 0.45 | 0.01733 |
| *Zbtb39* | zinc finger and BTB domain containing 39 | 3.28 | 0.00772 |
| *Zbtb7a* | zinc finger and BTB domain containing 7a | 0.68 | 0.01955 |
| *Zbtb8os* | zinc finger and BTB domain containing 8 opposite strand | 1.54 | 0.04534 |
| *Zcchc11* | zinc finger_ CCHC domain containing 11 | 1.61 | 0.00259 |
| *Zcchc14* | zinc finger_ CCHC domain containing 14 | 0.45 | 0.03886 |
| *Zcchc7* | zinc finger_ CCHC domain containing 7 | 1.15 | 0.02337 |
| *Zcwcc1* | microrchidia 2A | 0.74 | 0.00334 |
| *Zdhhc18* | zinc finger_ DHHC domain containing 18 | 0.72 | 0.01934 |
| *Zdhhc2* | zinc finger_ DHHC type containing 2 | 1.24 | 0.00813 |
| *Zdhhc21* | zinc finger_ DHHC type containing 21 | 0.53 | 0.02624 |
| *Zdhhc23* | zinc finger_ DHHC type containing 23 | 0.53 | 0.02649 |
| *Zdhhc3* | zinc finger_ DHHC type containing 3 | 0.81 | 0.033 |
| *Zdhhc8* | zinc finger_ DHHC type containing 8 | 0.48 | 0.02492 |
| *Zfand2a* | zinc finger_ AN1 type domain 2A | 0.78 | 0.00106 |
| *Zfand2b* | zinc finger_ AN1 type domain 2B | 0.66 | 0.0472 |
| *Zfp179* | ring finger protein 112 | 0.53 | 0.0012 |
| *Zfp265* | zinc finger, RAN-binding domain containing 2 | 0.79 | 0.02866 |
| *Zfp276* | zinc finger protein (C2H2 type) 276 | 0.47 | 0.00426 |
| *Zfp295* | zinc finger protein 295 | 1.46 | 0.00456 |
| *Zfp354a* | zinc finger protein 354A | 1.32 | 0.00748 |
| *Zfp354c* | zinc finger protein 354C | 0.78 | 0.03525 |
| *Zfp365* | zinc finger protein 365 | 1.89 | 0.02353 |
| *Zfp395* | zinc finger protein 395 | 0.4 | 0.00124 |
| *Zfp444* | zinc finger protein 444 | 1.22 | 0.04185 |
| *Zfp499* | zinc finger and BTB domain containing 45 | 1.5 | 0.00049 |
| *Zfp513* | zinc finger protein 513 | 0.76 | 0.00092 |
| *Zfp54* | zinc finger protein 54 | 0.46 | 0.00817 |
| *Zfp574* | zinc finger protein 574 | 0.17 | 0.00301 |
| *Zfp598* | zinc finger protein 598 | 0.67 | 0.01475 |
| *Zfp692* | zinc finger protein 692 | 0.87 | 0.02211 |
| *Zfp96* | zinc finger and SCAN domain containing 12 | 1.32 | 0.04191 |
| *Zfpn1a5* | IKAROS family zinc finger 5 | 1.42 | 0.02177 |
| *Zfx* | zinc finger protein X linked | 0.44 | 0.03817 |
| *Zhx1* | zinc fingers and homeoboxes 1 | 1.32 | 0.0195 |
| *Zmynd10* | zinc finger_ MYND type containing 10 | 0.77 | 0.01659 |
| *Znf142* | zinc finger protein 142 clone pHZ 49 | 0.76 | 0.01181 |
| *Znf174* | zinc finger protein 174 | 1.91 | 0.00958 |
| *Znf324* | zinc finger protein 324 | 0.28 | 0.00997 |
| *Znf532* | zinc finger protein 532 | 1.37 | 0.01548 |
| *Znf579* | zinc finger protein 579 | 0.72 | 0.00947 |
| *Znf618* | zinc finger protein 618 | 0.66 | 0.00717 |
| *Zswim6* | zinc finger_ SWIM type containing 6 | 0.68 | 0.01684 |
| *Zzef1* | zinc finger, ZZ-type with EF hand domain 1 | 0.69 | 0.00138 |
