# Supplementary material for: Increasing membrane cholesterol of neurons in culture recapitulates Alzheimer’s disease early phenotypes
Source: Mol Neurodegener. 2014 Dec 18;9:60. doi: 10.1186/1750-1326-9-60 (PMC4280040; doi:10.1186/1750-1326-9-60)
Supplement: Supplementary file 2 — Additional file 2: Enrichment of Gene Ontologies corresponding to genes differentially expressed after membrane cholesterol loading (enrichment p value <0.001) {Eden, 2007 #287; Eden, 2009 #286}. (DOCX 24 KB) [file 13024_2014_566_MOESM2_ESM.docx]

**Additional file 2.** Enrichment of Gene Ontologies corresponding to genes differentially expressed after membrane cholesterol loading (enrichment p value <0.001) {Eden, 2007 #287; Eden, 2009 #286}.

| **GO IDTerm p-value Number of genes** | | | | |
| --- | --- | --- | --- | --- |
| \| ***Cellular component*** \| \|  \|  \| \| --- \| --- \| --- \| --- \| \| GO:0005886 \| plasma membrane \| 2.61E-06 \| 276 \| \| GO:0044459 \| plasma membrane part \| 4.74E-05 \| 155 \| \| GO:0016020 \| membrane \| 7.13E-05 \| 472 \| \| GO:0016323 \| basolateral plasma membrane \| 1.55E-04 \| 28 \| \| GO:0031982 \| vesicle \| 1.99E-04 \| 114 \| \| GO:0045121 \| membrane raft \| 2.20E-04 \| 41 \| \| GO:0005829 \| cytosol \| 2.44E-04 \| 145 \| \| GO:0030175 \| filopodium \| 3.15E-04 \| 15 \| \| GO:0009986 \| cell surface \| 3.54E-04 \| 56 \| \| GO:0005737 \| cytoplasm \| 3.83E-04 \| 471 \| \| GO:0043005 \| neuron projection \| 4.81E-04 \| 114 \| \| GO:0042995 \| cell projection \| 4.92E-04 \| 155 \| \| GO:0031410 \| cytoplasmic vesicle \| 6.26E-04 \| 100 \| \|  \|  \|  \|  \| \| ***Biological Process*** \| \|  \|  \| \| GO:0065008 \| regulation of biological quality \| 2.07E-10 \| 249 \| \| GO:0009719 \| response to endogenous stimulus \| 9.89E-09 \| 167 \| \| GO:0050896 \| response to stimulus \| 1.22E-08 \| 559 \| \| GO:0010033 \| response to organic substance \| 1.84E-08 \| 235 \| \| GO:0009628 \| response to abiotic stimulus \| 3.06E-08 \| 138 \| \| GO:0033993 \| response to lipid \| 3.36E-08 \| 117 \| \| GO:0009605 \| response to external stimulus \| 3.72E-08 \| 134 \| \| GO:0042221 \| response to chemical stimulus \| 4.58E-08 \| 314 \| \| GO:0032502 \| developmental process \| 5.64E-08 \| 415 \| \| GO:0048878 \| chemical homeostasis \| 1.29E-07 \| 103 \| \| GO:0014070 \| response to organic cyclic compound \| 1.45E-07 \| 125 \| \| GO:0009987 \| cellular process \| 1.70E-07 \| 1033 \| \| GO:0042592 \| homeostatic process \| 4.86E-07 \| 130 \| \| GO:0051239 \| regulation of multicellular organismal process \| 5.18E-07 \| 230 \| \| GO:0044699 \| single-organism process \| 6.56E-07 \| 274 \| \| GO:0009725 \| response to hormone stimulus \| 6.56E-07 \| 121 \| \| GO:0010243 \| response to organic nitrogen \| 8.37E-07 \| 110 \| \| GO:0044707 \| single-multicellular organism process \| 1.26E-06 \| 217 \| \| GO:0048856 \| anatomical structure development \| 2.15E-06 \| 278 \| \| GO:0032501 \| multicellular organismal process \| 3.08E-06 \| 218 \| \| GO:0006873 \| cellular ion homeostasis \| 4.78E-06 \| 72 \| \| GO:0051716 \| cellular response to stimulus \| 4.92E-06 \| 403 \| \| GO:0055082 \| cellular chemical homeostasis \| 5.11E-06 \| 77 \| \| GO:0070887 \| cellular response to chemical stimulus \| 5.35E-06 \| 148 \| \| GO:0050801 \| ion homeostasis \| 5.87E-06 \| 77 \| \| GO:0016126 \| sterol biosynthetic process \| 7.86E-06 \| 14 \| \| GO:0048519 \| negative regulation of biological process \| 1.44E-05 \| 339 \| \| GO:0065007 \| biological regulation \| 1.58E-05 \| 766 \| \| GO:0048869 \| cellular developmental process \| 1.93E-05 \| 221 \| \| GO:0009991 \| response to extracellular stimulus \| 2.35E-05 \| 74 \| \| GO:0043066 \| negative regulation of apoptotic process \| 2.63E-05 \| 91 \| \| GO:0007166 \| cell surface receptor signaling pathway \| 2.64E-05 \| 175 \| \| GO:0007160 \| cell-matrix adhesion \| 2.71E-05 \| 13 \| \| GO:0006695 \| cholesterol biosynthetic process \| 2.72E-05 \| 12 \| \| GO:0043069 \| negative regulation of programmed cell death \| 3.29E-05 \| 91 \| \| GO:0009653 \| anatomical structure morphogenesis \| 3.29E-05 \| 159 \| \| GO:0032879 \| regulation of localization \| 3.73E-05 \| 170 \| \| GO:0048812 \| neuron projection morphogenesis \| 4.06E-05 \| 44 \| \| GO:0060548 \| negative regulation of cell death \| 4.09E-05 \| 96 \| \| GO:0019725 \| cellular homeostasis \| 4.44E-05 \| 84 \| \| GO:0008150 \| biological_process \| 4.59E-05 \| 1245 \| \| GO:0050793 \| regulation of developmental process \| 5.27E-05 \| 182 \| \| GO:0007165 \| signal transduction \| 6.68E-05 \| 296 \| \| GO:0050789 \| regulation of biological process \| 7.03E-05 \| 728 \| \| GO:0031667 \| response to nutrient levels \| 7.50E-05 \| 69 \| \| GO:0051128 \| regulation of cellular component organization \| 7.55E-05 \| 168 \| \| GO:0048545 \| response to steroid hormone stimulus \| 7.63E-05 \| 70 \| \| GO:0045822 \| negative regulation of heart contraction \| 8.18E-05 \| 9 \| \| GO:0044057 \| regulation of system process \| 8.45E-05 \| 79 \| \| GO:0046165 \| alcohol biosynthetic process \| 9.42E-05 \| 21 \| \| GO:0031644 \| regulation of neurological system process \| 9.86E-05 \| 52 \| \| GO:0051050 \| positive regulation of transport \| 1.02E-04 \| 74 \| \| GO:0016043 \| cellular component organization \| 1.18E-04 \| 348 \| \| GO:0010035 \| response to inorganic substance \| 1.18E-04 \| 81 \| \| GO:0043009 \| chordate embryonic development \| 1.23E-04 \| 45 \| \| GO:0048646 \| anatomical structure formation involved in morphogenesis \| 1.29E-04 \| 155 \| \| GO:0048523 \| negative regulation of cellular process \| 1.35E-04 \| 311 \| \| GO:0051049 \| regulation of transport \| 1.37E-04 \| 129 \| \| GO:0043087 \| regulation of GTPase activity \| 1.45E-04 \| 35 \| \| GO:0090066 \| regulation of anatomical structure size \| 1.45E-04 \| 35 \| \| GO:0007154 \| cell communication \| 1.58E-04 \| 71 \| \| GO:0032496 \| response to lipopolysaccharide \| 1.64E-04 \| 38 \| \| GO:0044087 \| regulation of cellular component biogenesis \| 1.80E-04 \| 57 \| \| GO:0003008 \| system process \| 1.91E-04 \| 99 \| \| GO:0071840 \| cellular component organization or biogenesis \| 1.93E-04 \| 353 \| \| GO:1900542 \| regulation of purine nucleotide metabolic process \| 2.06E-04 \| 49 \| \| GO:0033124 \| regulation of GTP catabolic process \| 2.11E-04 \| 35 \| \| GO:0009792 \| embryo development ending in birth or egg hatching \| 2.29E-04 \| 45 \| \| GO:0006810 \| transport \| 2.45E-04 \| 273 \| \| GO:0051234 \| establishment of localization \| 2.49E-04 \| 279 \| \| GO:0009607 \| response to biotic stimulus \| 2.75E-04 \| 59 \| \| GO:2000147 \| positive regulation of cell motility \| 2.76E-04 \| 38 \| \| GO:0040007 \| growth \| 2.84E-04 \| 50 \| \| GO:0040017 \| positive regulation of locomotion \| 2.97E-04 \| 39 \| \| GO:0030335 \| positive regulation of cell migration \| 3.02E-04 \| 37 \| \| GO:0030811 \| regulation of nucleotide catabolic process \| 3.02E-04 \| 37 \| \| GO:0033121 \| regulation of purine nucleotide catabolic process \| 3.02E-04 \| 37 \| \| GO:0009894 \| regulation of catabolic process \| 3.07E-04 \| 75 \| \| GO:0023051 \| regulation of signaling \| 3.23E-04 \| 215 \| \| GO:0001701 \| in utero embryonic development \| 3.24E-04 \| 43 \| \| GO:0009118 \| regulation of nucleoside metabolic process \| 3.58E-04 \| 37 \| \| GO:0006140 \| regulation of nucleotide metabolic process \| 3.61E-04 \| 49 \| \| GO:0071842 \| cellular component organization at cellular level \| 3.74E-04 \| 288 \| \| GO:0055085 \| transmembrane transport \| 3.96E-04 \| 59 \| \| GO:0002237 \| response to molecule of bacterial origin \| 4.10E-04 \| 39 \| \| GO:0010646 \| regulation of cell communication \| 4.16E-04 \| 214 \| \| GO:0042493 \| response to drug \| 4.19E-04 \| 74 \| \| GO:0071310 \| cellular response to organic substance \| 4.24E-04 \| 108 \| \| GO:0050794 \| regulation of cellular process \| 4.43E-04 \| 681 \| \| GO:0051272 \| positive regulation of cellular component movement \| 4.51E-04 \| 38 \| \| GO:0018193 \| peptidyl-amino acid modification \| 4.64E-04 \| 64 \| \| GO:0071495 \| cellular response to endogenous stimulus \| 4.67E-04 \| 66 \| \| GO:0003018 \| vascular process in circulatory system \| 4.75E-04 \| 16 \| \| GO:0019216 \| regulation of lipid metabolic process \| 4.96E-04 \| 37 \| \| GO:0042310 \| vasoconstriction \| 5.94E-04 \| 7 \| \| GO:0031668 \| cellular response to extracellular stimulus \| 5.99E-04 \| 29 \| \| GO:0030003 \| cellular cation homeostasis \| 6.05E-04 \| 44 \| \| GO:0071841 \| cellular component organization or biogenesis at cellular level \| 6.15E-04 \| 293 \| \| GO:0008045 \| motor neuron axon guidance \| 6.18E-04 \| 9 \| \| GO:0006812 \| cation transport \| 6.44E-04 \| 66 \| \| GO:0050848 \| regulation of calcium-mediated signaling \| 6.44E-04 \| 8 \| \| GO:0009612 \| response to mechanical stimulus \| 6.67E-04 \| 32 \| \| GO:0003013 \| circulatory system process \| 6.77E-04 \| 18 \| \| GO:0048584 \| positive regulation of response to stimulus \| 6.81E-04 \| 115 \| \| GO:0051493 \| regulation of cytoskeleton organization \| 6.94E-04 \| 44 \| \| GO:0044237 \| cellular metabolic process \| 7.24E-04 \| 601 \| \| GO:0008217 \| regulation of blood pressure \| 7.39E-04 \| 23 \| \| GO:0042940 \| D-amino acid transport \| 7.46E-04 \| 4 \| \| GO:0070884 \| regulation of calcineurin-NFAT signaling cascade \| 7.46E-04 \| 4 \| \| GO:0010959 \| regulation of metal ion transport \| 7.75E-04 \| 34 \| \| GO:0009790 \| embryo development \| 7.79E-04 \| 57 \| \| GO:0055080 \| cation homeostasis \| 7.91E-04 \| 49 \| \| GO:0051241 \| negative regulation of multicellular organismal process \| 7.95E-04 \| 44 \| \| GO:0016125 \| sterol metabolic process \| 8.71E-04 \| 19 \| \| GO:0006970 \| response to osmotic stress \| 8.79E-04 \| 16 \| \| GO:0060359 \| response to ammonium ion \| 8.87E-04 \| 18 \| \| GO:0044710 \| single-organism metabolic process \| 9.09E-04 \| 641 \| \| GO:0051969 \| regulation of transmission of nerve impulse \| 9.56E-04 \| 46 \| \| GO:2000026 \| regulation of multicellular organismal development \| 9.78E-04 \| 142 \| \| GO:0007584 \| response to nutrient \| 9.95E-04 \| 48 \| \|  \|  \|  \|  \| \| ***Molecular Function*** \| \|  \|  \| \| GO:0005102 \| receptor binding \| 6.42E-10 \| 159 \| \| GO:0005515 \| protein binding \| 3.51E-08 \| 602 \| \| GO:0005488 \| binding \| 2.31E-06 \| 986 \| \| GO:0019904 \| protein domain specific binding \| 3.30E-05 \| 99 \| \| GO:0003674 \| molecular_function \| 3.33E-05 \| 1261 \| \| GO:0051427 \| hormone receptor binding \| 4.43E-05 \| 28 \| \| GO:0015296 \| anion:cation symporter activity \| 8.18E-05 \| 9 \| \| GO:0032403 \| protein complex binding \| 1.45E-04 \| 75 \| \| GO:0043548 \| phosphatidylinositol 3-kinase binding \| 1.80E-04 \| 10 \| \| GO:0001883 \| purine nucleoside binding \| 2.37E-04 \| 202 \| \| GO:0035639 \| purine ribonucleoside triphosphate binding \| 2.51E-04 \| 200 \| \| GO:0001882 \| nucleoside binding \| 2.75E-04 \| 203 \| \| GO:0032550 \| purine ribonucleoside binding \| 2.91E-04 \| 201 \| \| GO:0005158 \| insulin receptor binding \| 2.91E-04 \| 11 \| \| GO:0001664 \| G-protein coupled receptor binding \| 3.24E-04 \| 32 \| \| GO:0032549 \| ribonucleoside binding \| 3.46E-04 \| 201 \| \| GO:0005524 \| ATP binding \| 4.22E-04 \| 164 \| \| GO:0043167 \| ion binding \| 4.31E-04 \| 489 \| \| GO:0017076 \| purine nucleotide binding \| 5.43E-04 \| 203 \| \| GO:0032555 \| purine ribonucleotide binding \| 5.59E-04 \| 202 \| \| GO:0051428 \| peptide hormone receptor binding \| 5.94E-04 \| 7 \| \| GO:0015172 \| acidic amino acid transmembrane transporter activity \| 6.37E-04 \| 5 \| \| GO:0030554 \| adenyl nucleotide binding \| 7.63E-04 \| 167 \| \| GO:0032553 \| ribonucleotide binding \| 7.94E-04 \| 203 \| \| GO:0043168 \| anion binding \| 8.05E-04 \| 255 \| \| GO:0032559 \| adenyl ribonucleotide binding \| 8.33E-04 \| 166 \| | | | | |
|  |  |  |  |  |
|  |  |  |  |  |
