# Supplementary material for: Increasing membrane cholesterol of neurons in culture recapitulates Alzheimer’s disease early phenotypes
Source: Mol Neurodegener. 2014 Dec 18;9:60. doi: 10.1186/1750-1326-9-60 (PMC4280040; doi:10.1186/1750-1326-9-60)
Supplement: Supplementary file 3 — Additional file 3: Genes differentially expressed after membrane cholesterol loading of cultured hippocampal neurons (t test, p < 0.05) and in postmortem AD brains from Braak stages 0 to VI (data of {Bossers, 2010 #269}; ANOVA, p < 0.05). (DOCX 44 KB) [file 13024_2014_566_MOESM3_ESM.docx]

**Additional file 3.** Genes differentially expressed after membrane cholesterol loading of cultured hippocampal neurons (t test, p < 0.05) and in postmortem AD brains from Braak stages 0 to VI (data of {Bossers, 2010 #269}; ANOVA, p < 0.05).

| **Gene acronym** | **Gene Name** | **Mean ratio cholesterol/ control** | **p value**  **cholesterol**  **(t-test)** | **p value**  **Braak stages**  **(ANOVA)** |
| --- | --- | --- | --- | --- |
| *Abcc8* | ATP-binding cassette, sub-family C (CFTR/MRP), member 8 | 0.78 | 0.00381 | 0.00110314 |
| *Abcf1* | ATP binding cassette sub family F GCN20 member 1 | 0.73 | 0.01299 | 0.03888368 |
| *Abl1* | c abl oncogene 1 receptor tyrosine kinase | 0.5 | 0.00567 | 0.0056101 |
| *Acat2* | acetyl Coenzyme A acetyltransferase 2 | 0.32 | 0.00235 | 0.03093967 |
| *Acn9* | ACN9 homolog S. cerevisiae | 1.54 | 0.01172 | 0.01517744 |
| *Actr1a* | ARP1 actin related protein 1 homolog A centractin alpha yeast | 0.86 | 0.0081 | 2.18E-05 |
| *Actr6* | ARP6 actin related protein 6 homolog yeast | 1.3 | 0.03881 | 0.01286523 |
| *Adipor2* | adiponectin receptor 2 | 0.55 | 0.03107 | 0.00041738 |
| *Adnp* | activity dependent neuroprotector homeobox | 0.77 | 0.01432 | 0.01830875 |
| *Adss* | adenylosuccinate synthase | 1.17 | 0.04059 | 0.00515141 |
| *Akap8l* | A kinase PRKA anchor protein 8 like | 0.81 | 0.00388 | 0.00400047 |
| *Angpt1* | angiopoietin 1 | 2.31 | 0.00576 | 0.0002471 |
| *Ankrd10* | ankyrin repeat domain 10 | 0.71 | 0.03707 | 0.00261385 |
| *Ankrd42* | ankyrin repeat domain 42 | 1.4 | 0.03006 | 0.036304 |
| *Apba3* | amyloid beta A4 precursor protein binding family A member 3 | 0.75 | 0.03836 | 0.03040495 |
| *Apln* | apelin | 1.53 | 0.01024 | 0.00396885 |
| *Arhgap21* | Rho GTPase activating protein 21 | 0.71 | 0.00657 | 5.62E-06 |
| *Arhgef1* | Rho guanine nucleotide exchange factor GEF 1 | 0.7 | 0.03216 | 0.01305198 |
| *Armcx2* | armadillo repeat containing X linked 2 | 0.76 | 0.03803 | 0.04647234 |
| *Arpc5l* | actin related protein 2/3 complex subunit 5 like | 0.8 | 0.03756 | 0.02125881 |
| *Asb2* | ankyrin repeat and SOCS box containing 2 | 2.26 | 0.0056 | 0.04635193 |
| *Asb6* | ankyrin repeat and SOCS box containing 6 | 0.76 | 0.01787 | 0.00323701 |
| *Asmtl* | acetylserotonin O methyltransferase like | 0.85 | 0.04634 | 0.00028182 |
| *Atp1b2* | ATPase Na+/K+ transporting beta 2 polypeptide | 1.24 | 0.03072 | 0.02396702 |
| *Atp1b3* | ATPase Na+/K+ transporting beta 3 polypeptide | 0.86 | 0.02411 | 0.01147254 |
| *Atp5j* | ATP synthase H+ transporting mitochondrial F0 complex subunit F6 | 1.4 | 0.01416 | 0.00568609 |
| *Atp6v1e1* | ATPase H+ transporting lysosomal V1 subunit E1 | 1.4 | 0.0037 | 0.03359928 |
| *Axin1* | axin 1 | 0.65 | 0.01075 | 0.00180858 |
| *Baz1a* | bromodomain adjacent to zinc finger domain 1A | 0.62 | 0.00822 | 0.02938316 |
| *Bcas1* | breast carcinoma amplified sequence 1 | 2.74 | 0.01001 | 0.03373129 |
| *Bcl2l2* | Bcl2 like 2 | 1.18 | 0.04065 | 0.0305405 |
| *Bdnf* | brain derived neurotrophic factor | 1.35 | 0.01808 | 0.00095503 |
| *Bfsp1* | beaded filament structural protein 1 | 1.85 | 0.01168 | 0.00961815 |
| *Bhlhb3* | basic helix-loop-helix family, member e41 | 1.57 | 0.04141 | 0.01668623 |
| *Birc6* | baculoviral IAP repeat containing 6 | 0.71 | 0.02509 | 0.01763466 |
| *Bmpr1a* | bone morphogenetic protein receptor type IA | 1.44 | 0.02151 | 0.00119762 |
| *Brca2* | breast cancer 2 | 1.29 | 0.01188 | 0.0427594 |
| *Brd2* | bromodomain containing 2 | 1.37 | 0.03395 | 0.04050477 |
| *Brd4* | bromodomain containing 4 | 0.73 | 0.01413 | 0.03382659 |
| *Bri3* | brain protein I3 | 0.76 | 0.02607 | 0.04276788 |
| *Brp44l* | brain protein 44 like | 1.15 | 0.01036 | 0.03314057 |
| *Camk4* | calcium/calmodulin dependent protein kinase IV | 1.5 | 0.02915 | 0.01061362 |
| *Ccdc21* | coiled coil domain containing 21 | 0.66 | 0.00152 | 0.00682379 |
| *Ccng1* | cyclin G1 | 0.71 | 0.01868 | 0.028168 |
| *Cd248* | CD248 molecule endosialin | 0.4 | 0.04762 | 0.03610105 |
| *Cdc27* | cell division cycle 27 homolog S. cerevisiae | 0.62 | 0.02014 | 0.04902672 |
| *Chd4* | chromodomain helicase DNA binding protein 4 | 0.67 | 0.0062 | 0.00091248 |
| *Chst12* | carbohydrate sulfotransferase 12 | 1.19 | 0.03049 | 0.00353827 |
| *Cited1* | Cbp/p300 interacting transactivator with Glu/Asp rich carboxy terminal domain 1 | 1.65 | 0.02931 | 0.00262709 |
| *Cmkor1* | chemokine (C-X-C motif) receptor 7 | 0.61 | 0.00561 | 0.00835594 |
| *Cog3* | component of oligomeric golgi complex 3 | 0.84 | 0.00698 | 0.01859241 |
| *Cog8* | component of oligomeric golgi complex 8 | 0.81 | 0.04453 | 0.01230883 |
| *Col5a2* | collagen type V alpha 2 | 1.43 | 0.01611 | 0.01118207 |
| *Commd4* | COMM domain containing 4 | 1.08 | 0.02279 | 0.04816206 |
| *Cops7a* | COP9 constitutive photomorphogenic homolog subunit 7A Arabidopsis | 1.62 | 0.00667 | 0.00030318 |
| *Coro1a* | coronin actin binding protein 1A | 0.65 | 0.02142 | 0.02418437 |
| *Cpt1b* | carnitine palmitoyltransferase 1b muscle | 0.66 | 0.02936 | 0.0009352 |
| *Cryab* | crystallin alpha B | 1.98 | 0.01368 | 0.00621974 |
| *Crygd* | crystallin gamma D | 1.53 | 0.02456 | 0.04622023 |
| *Ctdp1* | CTD carboxy terminal domain RNA polymerase II polypeptide A phosphatase subunit 1 | 0.61 | 0.04984 | 0.02554586 |
| *Ctnnb1* | catenin cadherin associated protein beta 1 | 1.42 | 0.03571 | 0.02026532 |
| *Ctrc* | chymotrypsin C caldecrin | 0.56 | 0.03117 | 0.01466687 |
| *Cyln2* | CAP-GLY domain containing linker protein 2 | 0.86 | 0.00099 | 1.54E-05 |
| *Cyp26b1* | cytochrome P450 family 26 subfamily b polypeptide 1 | 0.5 | 0.0075 | 0.03178595 |
| *Dbn1* | drebrin 1 | 0.64 | 1.00E-05 | 0.02694116 |
| *Dchs1* | dachsous 1 Drosophila | 0.86 | 0.04366 | 0.01804233 |
| *Ddit4* | DNA damage inducible transcript 4 | 0.57 | 0.0055 | 0.00106826 |
| *Deaf1* | deformed epidermal autoregulatory factor 1 Drosophila | 0.81 | 0.0083 | 0.04922863 |
| *Dek* | DEK oncogene | 1.26 | 0.01877 | 0.0005977 |
| *Dhcr7* | 7 dehydrocholesterol reductase | 0.41 | 0.0278 | 0.00079383 |
| *Dhx15* | DEAH Asp Glu Ala His box polypeptide 15 | 0.81 | 0.04029 | 0.01532405 |
| *Dio2* | deiodinase iodothyronine type II | 0.64 | 0.00895 | 0.01124597 |
| *Dio3* | deiodinase iodothyronine type III | 1.52 | 0.03153 | 0.03550564 |
| *Dmn* | synemin, intermediate filament protein | 1.89 | 0.01786 | 9.14E-05 |
| *Dpp6* | dipeptidylpeptidase 6 | 1.29 | 0.03535 | 0.02191163 |
| *Dscr2* | proteasome (prosome, macropain) assembly chaperone 1 | 0.84 | 0.04518 | 0.00783504 |
| *Elavl1* | ELAV embryonic lethal abnormal vision Drosophila like 1 Hu antigen R | 0.81 | 0.00723 | 0.03198062 |
| *Elf2* | E74 like factor 2 | 0.45 | 0.04987 | 0.02168558 |
| *Elovl7* | ELOVL family member 7 elongation of long chain fatty acids yeast | 1.56 | 0.02444 | 0.01477546 |
| *Erbb2* | v erb b2 erythroblastic leukemia viral oncogene homolog 2 neuro/glioblastoma derived oncogene homolog avian | 1.9 | 0.00181 | 0.03351226 |
| *Ercc5* | excision repair cross complementing rodent repair deficiency complementation group 5 | 1.47 | 0.03749 | 0.02199065 |
| *Etv5* | ets variant 5 | 0.64 | 0.03003 | 0.00284747 |
| *Fbl* | fibrillarin | 0.79 | 0.02315 | 0.00110313 |
| *Fbxo30* | F box protein 30 | 0.76 | 0.01618 | 0.01890615 |
| *Fcho1* | FCH domain only 1 | 0.42 | 0.00736 | 6.26E-05 |
| *Fkbp3* | FK506 binding protein 3 | 1.22 | 0.01539 | 0.0148199 |
| *Fkbp5* | FK506 binding protein 5 | 0.4 | 0.03693 | 0.01401092 |
| *Flcn* | folliculin | 1.15 | 0.03854 | 0.01892023 |
| *Fmod* | fibromodulin | 1.64 | 0.043 | 0.04608275 |
| *Fuk* | fucokinase | 0.46 | 0.01037 | 0.0098327 |
| *Fundc1* | FUN14 domain containing 1 | 1.45 | 0.04399 | 0.01765833 |
| *Fyn* | FYN oncogene related to SRC FGR YES | 1.58 | 0.02038 | 0.04507902 |
| *Fzd1* | frizzled homolog 1 Drosophila | 0.79 | 0.04983 | 0.01573867 |
| *Fzd9* | frizzled homolog 9 Drosophila | 0.5 | 0.02267 | 0.02815844 |
| *Gale* | UDP galactose 4 epimerase | 0.74 | 0.0307 | 0.03046234 |
| *Galnt11* | UDP N acetyl alpha D galactosamine:polypeptide N acetylgalactosaminyltransferase 11 GalNAc T11 | 0.77 | 0.00864 | 0.00881678 |
| *Gfap* | glial fibrillary acidic protein | 1.61 | 0.02819 | 0.00514687 |
| *Gfra2* | GDNF family receptor alpha 2 | 0.47 | 0.01046 | 0.00247438 |
| *Git1* | G protein coupled receptor kinase interacting ArfGAP 1 | 0.74 | 0.01044 | 0.00272876 |
| *Gja1* | gap junction protein alpha 1 | 1.98 | 0.02816 | 0.02335019 |
| *Glud1* | glutamate dehydrogenase 1 | 1.31 | 0.02513 | 0.02476042 |
| *Gnrh1* | gonadotropin releasing hormone 1 luteinizing releasing hormone | 0.68 | 0.02029 | 0.02081021 |
| *Gphn* | gephyrin | 1.67 | 0.0076 | 0.01454234 |
| *Gtf2ird1* | GTF2I repeat domain containing 1 | 0.68 | 0.00025 | 0.03673573 |
| *Gulp1* | GULP engulfment adaptor PTB domain containing 1 | 2.13 | 0.00608 | 0.00668819 |
| *Hagh* | hydroxyacyl glutathione hydrolase | 0.74 | 0.0011 | 0.04994917 |
| *Hbp1* | HMG box transcription factor 1 | 1.83 | 0.02098 | 0.00025782 |
| *Hdac8* | histone deacetylase 8 | 1.36 | 0.01175 | 0.01382367 |
| *Hexim2* | hexamthylene bis acetamide inducible 2 | 0.58 | 0.01026 | 0.03855206 |
| *Hig1* | HIG1 domain family, member 1A | 0.68 | 0.01286 | 0.02286169 |
| *Hirip3* | HIRA interacting protein 3 | 1.43 | 0.0396 | 0.01385707 |
| *Hist1h2bh* | histone cluster 1 H2bh | 1.45 | 0.0099 | 0.00895681 |
| *Hist1h2bm* | histone cluster 1 H2bm | 1.46 | 0.00427 | 0.00175663 |
| *Hist1h2bn* | histone cluster 1, H2bn | 1.27 | 0.04113 | 0.01092352 |
| *Hmgcs1* | 3 hydroxy 3 methylglutaryl Coenzyme A synthase 1 soluble | 0.58 | 0.01397 | 0.00749711 |
| *Hmgn2* | high mobility group nucleosomal binding domain 2 | 1.22 | 0.02027 | 0.01641988 |
| *Hmox2* | heme oxygenase decycling 2 | 0.88 | 0.04469 | 0.02291545 |
| *Hnrpf* | heterogeneous nuclear ribonucleoprotein F | 1.32 | 0.01152 | 0.01091859 |
| *Hnrpu* | heterogeneous nuclear ribonucleoprotein U | 0.8 | 0.00971 | 0.00077777 |
| *Hsd17b1* | hydroxysteroid 17 beta dehydrogenase 1 | 0.56 | 0.04408 | 0.00228825 |
| *Hspa2* | heat shock protein 2 | 1.45 | 0.0292 | 2.61E-05 |
| *Hspb1* | heat shock protein 1 | 1.39 | 0.00088 | 0.02403038 |
| *Htr7* | 5 hydroxytryptamine serotonin receptor 7 | 1.89 | 0.01007 | 0.00795268 |
| *Ica1* | islet cell autoantigen 1 | 1.5 | 0.01256 | 0.00117682 |
| *Idi1* | isopentenyl diphosphate delta isomerase 1 | 0.38 | 0.03937 | 0.02341657 |
| *Inhbb* | inhibin beta B | 1.39 | 0.00311 | 0.00612998 |
| *Irf3* | interferon regulatory factor 3 | 0.73 | 0.01582 | 0.04636623 |
| *Itgb1* | integrin beta 1 | 0.86 | 0.03397 | 0.04401439 |
| *Itgb8* | integrin beta 8 | 1.75 | 0.01136 | 0.00021751 |
| *Ivns1abp* | influenza virus NS1A binding protein | 0.85 | 0.00894 | 0.03914714 |
| *Jund* | jun D proto oncogene | 1.5 | 0.03289 | 0.04410425 |
| *Katna1* | katanin p60 ATPase containing subunit A1 | 0.76 | 0.03218 | 0.0353097 |
| *Kcna2* | potassium voltage gated channel shaker related subfamily member 2 | 1.51 | 0.02624 | 0.01766931 |
| *Kcnq3* | potassium voltage-gated channel, subfamily Q, member 3 | 2.57 | 0.00855 | 0.00068079 |
| *Kctd3* | potassium channel tetramerisation domain containing 3 | 1.3 | 0.04387 | 0.0012963 |
| *Klhl2* | kelch-like 2, Mayven (Drosophila) | 0.64 | 0.00361 | 0.00653127 |
| *Lbr* | lamin B receptor | 0.64 | 0.03795 | 0.0010834 |
| *Lcat* | lecithin cholesterol acyltransferase | 0.77 | 0.03264 | 0.0042318 |
| *Litaf* | lipopolysaccharide induced TNF factor | 1.28 | 0.0169 | 0.04866795 |
| *Lrdd* | leucine rich repeats and death domain containing | 0.69 | 0.03138 | 0.00014718 |
| *Lrfn3* | leucine rich repeat and fibronectin type III domain containing 3 | 0.56 | 9.00E-05 | 0.04191082 |
| *Lrp10* | low density lipoprotein receptor related protein 10 | 0.72 | 0.00222 | 0.01712364 |
| *Lrp2* | low density lipoprotein related protein 2 | 0.71 | 0.02577 | 0.02835297 |
| *Map1lc3a* | microtubule associated protein 1 light chain 3 alpha | 0.98 | 0.00563 | 0.00409445 |
| *Mapk7* | mitogen-activated protein kinase 7 | 0.72 | 0.0061 | 0.00812936 |
| *Mat2a* | methionine adenosyltransferase II alpha | 0.69 | 0.03273 | 0.00254299 |
| *Mbd6* | methyl CpG binding domain protein 6 | 0.74 | 0.0248 | 0.00671912 |
| *Mcm3ap* | minichromosome maintenance deficient 3 S. cerevisiae associated protein | 0.74 | 0.02481 | 0.04262275 |
| *Mcoln1* | mucolipin 1 | 0.76 | 0.02411 | 0.02618472 |
| *Mecr* | mitochondrial trans 2 enoyl CoA reductase | 0.58 | 0.03168 | 4.01E-06 |
| *Metap1* | methionyl aminopeptidase 1 | 0.77 | 0.02408 | 0.01166608 |
| *Morc3* | microrchidia 3 | 0.57 | 0.02271 | 0.01975828 |
| *Mpdu1* | multiple PDZ domain protein | 1.21 | 0.0456 | 0.03231067 |
| *Mpi* | mannose phosphate isomerase | 0.6 | 0.02451 | 0.00055793 |
| *Mrpl12* | mitochondrial ribosomal protein L12 | 0.82 | 0.02731 | 0.04865684 |
| *Mrpl48* | mitochondrial ribosomal protein L48 | 1.28 | 0.04893 | 0.02399869 |
| *Mrpl9* | mitochondrial ribosomal protein L9 | 1.41 | 0.01432 | 0.03430218 |
| *Mrps30* | mitochondrial ribosomal protein S30 | 1.23 | 0.04661 | 0.04809312 |
| *Mrps33* | mitochondrial ribosomal protein S33 | 1.5 | 0.00495 | 0.00601755 |
| *Mrps36* | mitochondrial ribosomal protein S36 | 1.18 | 0.02825 | 0.00837722 |
| *Msx1* | msh homeobox 1 | 2.12 | 0.00229 | 0.04030957 |
| *Mthfd1* | methylenetetrahydrofolate dehydrogenase NADP+ dependent 1 methenyltetrahydrofolate cyclohydrolase formyltetrahydrofolate synthetase | 0.79 | 0.03608 | 0.00247533 |
| *Mutyh* | mutY homolog E. coli | 0.65 | 0.01076 | 0.01763193 |
| *Mxd4* | Max dimerization protein 4 | 1.53 | 0.03516 | 0.03058963 |
| *Mxi1* | MAX interactor 1 | 1.45 | 0.02149 | 1.72E-05 |
| *Myo9b* | myosin IXb | 0.69 | 0.03686 | 0.00619702 |
| *Myst3* | MYST histone acetyltransferase monocytic leukemia 3 | 0.6 | 0.03216 | 0.00067228 |
| *Ncald* | neurocalcin delta | 1.83 | 0.01747 | 0.0152871 |
| *Ncoa1* | nuclear receptor coactivator 1 | 1.24 | 0.01104 | 0.00485941 |
| *Ndufb2* | NADH dehydrogenase ubiquinone 1 beta subcomplex 2 | 1.33 | 0.02603 | 0.01597196 |
| *Ndufb9* | NADH dehydrogenase ubiquinone 1 beta subcomplex 9 | 0.84 | 0.0057 | 0.00313251 |
| *Ndufc2* | NADH dehydrogenase ubiquinone 1 subcomplex unknown 2 | 1.2 | 0.03101 | 0.04889336 |
| *Necap2* | NECAP endocytosis associated 2 | 0.79 | 0.00654 | 0.04061932 |
| *Nefl* | neurofilament light polypeptide | 1.85 | 0.02011 | 0.04701238 |
| *Nfat5* | nuclear factor of activated T cells 5 | 0.77 | 0.03209 | 0.01876704 |
| *Nfkbia* | nuclear factor of kappa light polypeptide gene enhancer in B cells inhibitor alpha | 0.71 | 0.01773 | 0.00045457 |
| *Nid67* | putative small membrane protein NID67 | 0.84 | 0.03494 | 0.00200396 |
| *Nit1* | nitrilase 1 | 1.31 | 0.03572 | 0.02975194 |
| *Nolc1* | nucleolar and coiled body phosphoprotein 1 | 0.62 | 0.00118 | 0.03279024 |
| *Nov* | nephroblastoma overexpressed gene | 1.8 | 0.04922 | 0.03962524 |
| *Npff* | neuropeptide FF amide peptide precursor | 0.82 | 0.04309 | 0.0021608 |
| *Nrcam* | neuronal cell adhesion molecule | 0.36 | 0.00335 | 0.01422313 |
| *Nrxn3* | neurexin 3 | 1.78 | 0.00888 | 0.00062172 |
| *Nubp1* | nucleotide binding protein 1 | 0.8 | 0.02408 | 0.02441772 |
| *Nudt4* | nudix nucleoside diphosphate linked moiety X type motif 4 | 1.51 | 0.00531 | 0.02016849 |
| *Nup205* | nucleoporin 205 | 0.74 | 0.00484 | 0.04471137 |
| *Olfm1* | olfactomedin 1 | 0.99 | 0.02842 | 0.01447947 |
| *Pabpn1* | polyA binding protein nuclear 1 | 0.8 | 0.00325 | 0.00185762 |
| *Pdzk6* | inturned planar cell polarity effector homolog (Drosophila) | 1.16 | 0.0212 | 0.02301793 |
| *Pemt* | phosphatidylethanolamine N methyltransferase | 1.71 | 0.02362 | 0.00096932 |
| *Pfkfb1* | 6 phosphofructo 2 kinase/fructose 2 6 biphosphatase 1 | 0.68 | 0.00681 | 0.02882449 |
| *Phf17* | PHD finger protein 17 | 0.1 | 0.04389 | 0.01562948 |
| *Phlpp* | PH domain and leucine rich repeat protein phosphatase | 1.4 | 0.01817 | 0.01157657 |
| *Phyhd1* | phytanoyl CoA dioxygenase domain containing 1 | 0.51 | 0.03301 | 0.00241156 |
| *Pik3r2* | phosphoinositide 3 kinase regulatory subunit 2 beta | 0.8 | 0.04231 | 0.00600821 |
| *Pkd1* | polycystic kidney disease 1 homolog | 0.62 | 0.00563 | 0.03347549 |
| *Pkn1* | protein kinase N1 | 0.76 | 0.03125 | 0.00043179 |
| *Plagl2* | pleiomorphic adenoma gene like 2 | 0.77 | 0.01776 | 0.01503202 |
| *Plcb1* | phospholipase C beta 1 phosphoinositide specific | 1.45 | 0.0471 | 0.00185402 |
| *Plcg1* | phospholipase C gamma 1 | 0.76 | 0.04366 | 0.00031628 |
| *Plekhm1* | pleckstrin homology domain containing family M with RUN domain member 1 | 2.43 | 0.00054 | 0.01876945 |
| *Pltp* | phospholipid transfer protein | 1.84 | 0.02009 | 0.0452281 |
| *Pmch* | pro melanin concentrating hormone | 1.79 | 0.00722 | 0.0090401 |
| *Pnoc* | prepronociceptin | 5.22 | 0.00299 | 0.01858795 |
| *Pomt1* | protein O mannosyltransferase 1 | 0.73 | 0.0024 | 0.03536981 |
| *Ppfia2* | protein tyrosine phosphatase receptor type f polypeptide PTPRF interacting protein liprin alpha 2 | 1.44 | 0.0494 | 0.01596872 |
| *Ppfibp2* | PTPRF interacting protein binding protein 2 liprin beta 2 | 1.7 | 0.04059 | 0.00425069 |
| *Ppm1b* | protein phosphatase 1B magnesium dependent beta isoform | 1.39 | 0.00241 | 0.01510499 |
| *Ppp2r2b* | protein phosphatase 2 formerly 2A regulatory subunit B PR 52 beta isoform | 1.38 | 0.04303 | 0.03158144 |
| *Ppp4r1* | protein phosphatase 4 regulatory subunit 1 | 0.87 | 0.03985 | 0.01353927 |
| *Prosc* | proline synthetase co transcribed homolog bacterial | 1.31 | 0.04302 | 0.0132179 |
| *Pscd2* | cytohesin 2 | 0.7 | 0.00565 | 0.03071564 |
| *Psmd4* | proteasome prosome macropain 26S subunit non ATPase 4 | 0.75 | 0.0086 | 0.02387147 |
| *Ptk9* | twinfilin, actin-binding protein, homolog 1 (Drosophila) | 0.67 | 0.01449 | 0.01808486 |
| *Ptp4a1* | protein tyrosine phosphatase 4a1 | 0.71 | 0.02888 | 0.00925271 |
| *Ptpn1* | protein tyrosine phosphatase non receptor type 1 | 0.54 | 0.00539 | 0.03275053 |
| *Ptpn11* | protein tyrosine phosphatase non receptor type 11 | 0.67 | 0.03647 | 0.00285545 |
| *Rab10* | RAB10 member RAS oncogene family | 0.81 | 0.01467 | 0.0334289 |
| *Rab31* | RAB31 member RAS oncogene family | 1.21 | 0.03219 | 0.01130159 |
| *Rbm13* | MAK16 homolog (S. cerevisiae) | 0.48 | 0.00116 | 0.00666608 |
| *Rbp1* | retinol binding protein 1 cellular | 1.41 | 0.03232 | 0.00109564 |
| *Rbp4* | retinol binding protein 4 plasma | 2.54 | 0.00197 | 0.00388769 |
| *Rent1* | UPF1 regulator of nonsense transcripts homolog (yeast) | 0.6 | 0.0186 | 0.02640763 |
| *Rg9mtd3* | RNA guanine 9 methyltransferase domain containing 3 | 1.45 | 0.02031 | 0.02066561 |
| *Rgs4* | regulator of G protein signaling 4 | 1.87 | 0.00547 | 0.01837229 |
| *Rhebl1* | Ras homolog enriched in brain like 1 | 0.77 | 0.03346 | 0.01475886 |
| *Rhoq* | ras homolog gene family member Q | 0.61 | 0.04682 | 0.03304222 |
| *Rnf126* | ring finger protein 126 | 0.7 | 0.04875 | 0.04817735 |
| *Rnf14* | ring finger protein 14 | 0.96 | 0.00165 | 0.03034117 |
| *Rpa3* | replication protein A3 | 1.35 | 0.02403 | 0.00194346 |
| *S100a4* | S100 calcium binding protein A4 | 1.71 | 0.03528 | 0.00802634 |
| *Sall1* | sal like 1 Drosophila | 0.61 | 0.00419 | 0.00467313 |
| *Sall2* | sal like 2 Drosophila | 2.93 | 0.01454 | 0.00650532 |
| *Scg3* | secretogranin III | 1.26 | 0.00787 | 1.22E-06 |
| *Sdccag3* | serologically defined colon cancer antigen 3 | 0.64 | 0.00489 | 0.00067909 |
| *Sdhc* | succinate dehydrogenase complex subunit C integral membrane protein | 1.5 | 0.00568 | 0.00689848 |
| *Sec61g* | SEC61 gamma subunit | 1.27 | 0.04254 | 0.00610552 |
| *Set* | SET nuclear oncogene | 1.36 | 0.02 | 0.02508876 |
| *Sf3b2* | splicing factor 3b subunit 2 | 1.32 | 0.01382 | 0.00560504 |
| *Sfrs5* | splicing factor arginine/serine rich 5 | 1.41 | 9.00E-05 | 0.0108499 |
| *Sfxn4* | sideroflexin 4 | 0.87 | 0.01819 | 0.00681336 |
| *Sirt7* | sirtuin 7 silent mating type information regulation 2 homolog 7 S. cerevisiae | 0.57 | 0.00432 | 0.03849408 |
| *Skp1a* | S-phase kinase-associated protein 1A | 1.01 | 0.0416 | 0.04545062 |
| *Slc12a2* | solute carrier family 12 sodium/potassium/chloride transporters member 2 | 0.47 | 0.00028 | 0.04143041 |
| *Slc17a6* | solute carrier family 17 sodium dependent inorganic phosphate cotransporter member 6 | 0.78 | 0.01626 | 0.02520807 |
| *Slc1a2* | solute carrier family 1 glial high affinity glutamate transporter member 2 | 1.72 | 0.01013 | 0.01039631 |
| *Slc20a1* | solute carrier family 20 phosphate transporter member 1 | 0.41 | 0.00649 | 3.50E-05 |
| *Slc25a1* | solute carrier family 25 mitochondrial carrier citrate transporter member 1 | 0.57 | 0.0161 | 0.02968087 |
| *Slc25a5* | solute carrier family 25 mitochondrial carrier; adenine nucleotide translocator member 5 | 0.73 | 0.01251 | 8.29E-05 |
| *Slc2a1* | solute carrier family 2 facilitated glucose transporter member 1 | 0.68 | 0.04363 | 0.01719496 |
| *Slc35b1* | solute carrier family 35 member B1 | 0.52 | 0.00164 | 0.01212658 |
| *Slc4a4* | solute carrier family 4 anion exchanger member 4 | 2.62 | 0.00222 | 0.02337363 |
| *Slc7a4* | solute carrier family 7 cationic amino acid transporter y+ system member 4 | 0.42 | 0.00045 | 0.02841307 |
| *Slc7a8* | solute carrier family 7 cationic amino acid transporter y+ system member 8 | 1.43 | 0.02861 | 0.04176063 |
| *Slit1* | slit homolog 1 Drosophila | 0.59 | 0.04599 | 0.00423778 |
| *Snrp70* | small nuclear ribonucleoprotein 70 (U1) | 0.71 | 0.01112 | 0.00196648 |
| *Snrpd1* | small nuclear ribonucleoprotein D1 | 1.11 | 0.04848 | 0.04741629 |
| *Snx10* | sorting nexin 10 | 1.42 | 0.01645 | 0.02363051 |
| *Snx7* | sorting nexin 7 | 1.64 | 0.00434 | 0.00055751 |
| *Sorcs1* | sortilin related VPS10 domain containing receptor 1 | 0.58 | 0.01659 | 0.0228297 |
| *Sorcs3* | sortilin related VPS10 domain containing receptor 3 | 2.18 | 0.00095 | 0.04446356 |
| *Sox10* | SRY sex determining region Y box 10 | 2.81 | 0.01143 | 0.00626766 |
| *Spg20* | spastic paraplegia 20 Troyer syndrome homolog human | 1.78 | 0.00616 | 0.00513758 |
| *Spg21* | spastic paraplegia 21 homolog human | 0.77 | 0.04867 | 0.00304958 |
| *Spire1* | spire homolog 1 Drosophila | 0.74 | 0.01936 | 2.20E-06 |
| *Srprb* | signal recognition particle receptor B subunit | 0.88 | 0.03786 | 0.04291723 |
| *Srrm2* | serine/arginine repetitive matrix 2 | 0.78 | 0.0108 | 0.00170064 |
| *Stat3* | signal transducer and activator of transcription 3 | 0.72 | 0.02887 | 0.04363306 |
| *Stk38* | serine/threonine kinase 38 | 0.56 | 8.00E-05 | 0.04213629 |
| *Stk39* | serine/threonine kinase 39 STE20/SPS1 homolog yeast | 0.7 | 0.01741 | 0.01676574 |
| *Stx1a* | syntaxin 1A brain | 0.73 | 0.02463 | 0.01039169 |
| *Syncrip* | synaptotagmin binding cytoplasmic RNA interacting protein | 0.74 | 0.04194 | 0.00634081 |
| *Syngr1* | synaptogyrin 1 | 1.31 | 0.02313 | 0.02663765 |
| *Tac1* | tachykinin 1 | 3.54 | 0.03497 | 0.00840031 |
| *Tbc1d19* | TBC1 domain family member 19 | 0.73 | 0.02865 | 0.04722545 |
| *Thbs4* | thrombospondin 4 | 0.11 | 0 | 0.02883819 |
| *Timm23* | translocase of inner mitochondrial membrane 23 homolog yeast | 0.81 | 0.00917 | 0.01860374 |
| *Timm8b* | translocase of inner mitochondrial membrane 8 homolog b yeast | 1.27 | 0.01189 | 0.00137103 |
| *Tk2* | thymidine kinase 2 mitochondrial | 1.32 | 0.00918 | 0.02559315 |
| *Tkt* | transketolase | 0.99 | 0.01086 | 0.00786017 |
| *Tle2* | transducin like enhancer of split 2 Esp1 homolog Drosophila | 0.57 | 0.00018 | 0.02977369 |
| *Tm7sf2* | transmembrane 7 superfamily member 2 | 0.56 | 0.04019 | 5.83E-05 |
| *Tmeff2* | transmembrane protein with EGF like and two follistatin like domains 2 | 1.78 | 0.02697 | 0.04465719 |
| *Tomm40* | translocase of outer mitochondrial membrane 40 homolog yeast | 0.56 | 0.01408 | 0.00148918 |
| *Tparl* | transmembrane protein 165 | 0.76 | 0.0287 | 0.04521314 |
| *Tpbg* | trophoblast glycoprotein | 0.73 | 0.03579 | 0.01321732 |
| *Tra1* | tumor rejection antigen gp96 | 0.51 | 0.02257 | 0.01832322 |
| *Trim45* | tripartite motif containing 45 | 1.76 | 0.02197 | 0.03853589 |
| *Trps1* | trichorhinophalangeal syndrome I homolog human | 1.44 | 0.0085 | 0.00431657 |
| *Ubap1* | ubiquitin associated protein 1 | 0.91 | 0.0083 | 0.00705 |
| *Uck1* | uridine cytidine kinase 1 | 0.79 | 0.00766 | 0.0003561 |
| *Vil2* | ezrin | 1.27 | 0.04341 | 0.00054574 |
| *Vmp* | neurensin 1 | 1.44 | 0.02711 | 0.03765428 |
| *Wbp5* | WW domain binding protein 5 | 0.83 | 0.03971 | 0.03529747 |
| *Wdr24* | WD repeat domain 24 | 0.65 | 0.00134 | 0.01009031 |
| *Wdr31* | WD repeat domain 31 | 1.56 | 0.02855 | 0.03377634 |
| *Wdr61* | WD repeat domain 61 | 0.82 | 0.00688 | 0.03116855 |
| *Wfdc2* | WAP four disulfide core domain 2 | 1.66 | 0.01062 | 5.16E-05 |
| *Wif1* | Wnt inhibitory factor 1 | 1.44 | 0.03174 | 0.00097149 |
| *Xpo1* | exportin 1 CRM1 homolog yeast | 0.67 | 0.00223 | 0.00965897 |
| *Ywhaz* | tyrosine 3 monooxygenase/tryptophan 5 monooxygenase activation protein zeta polypeptide | 0.81 | 0.04589 | 0.03086541 |
| *Zbtb1* | zinc finger and BTB domain containing 1 | 0.45 | 0.01733 | 0.00332934 |
| *Zbtb8os* | zinc finger and BTB domain containing 8 opposite strand | 1.54 | 0.04534 | 0.03124986 |
| *Zcchc11* | zinc finger CCHC domain containing 11 | 1.61 | 0.00259 | 0.00114235 |
| *Zcchc14* | zinc finger CCHC domain containing 14 | 0.45 | 0.03886 | 3.81E-05 |
| *Zdhhc23* | zinc finger DHHC type containing 23 | 0.53 | 0.02649 | 0.02626503 |
| *Zmynd10* | zinc finger MYND type containing 10 | 0.77 | 0.01659 | 0.01274931 |
| *Znf324* | zinc finger protein 324 | 0.28 | 0.00997 | 0.01347813 |
