# Supplementary material for: Increasing membrane cholesterol of neurons in culture recapitulates Alzheimer’s disease early phenotypes
Source: Mol Neurodegener. 2014 Dec 18;9:60. doi: 10.1186/1750-1326-9-60 (PMC4280040; doi:10.1186/1750-1326-9-60)
Supplement: Supplementary file 4 — Additional file 4: Gene Ontologies for the genes from cluster 1. (DOCX 17 KB) [file 13024_2014_566_MOESM4_ESM.docx]

**Additional file 4.** Gene Ontologies for the genes from cluster 1.

| **GO ID term** | **Number**  **of genes** |
| --- | --- |
| **Cellular component** | |
| mitochondrial part | 13 |
| mitochondrion | 16 |
| mitochondrial envelope | 9 |
| mitochondrial membrane | 8 |
| organelle membrane | 13 |
| organelle envelope | 9 |
| envelope | 9 |
| mitochondrial inner membrane | 6 |
| organelle inner membrane | 6 |
| mitochondrial membrane part | 4 |
| mitochondrial ribosome | 3 |
| organellar ribosome | 3 |
| clathrin coated vesicle membrane | 3 |
| axon | 4 |
| coated vesicle membrane | 3 |
| respiratory chain | 3 |
| ribosome | 4 |
| mitochondrial lumen | 4 |
| mitochondrial matrix | 4 |
| mitochondrial small ribosomal subunit | 2 |
| organellar small ribosomal subunit | 2 |
| synapse part | 4 |
| synaptic vesicle membrane | 2 |
|  |  |
| **Biological Process** | |
| synaptic transmission | 7 |
| transmission of nerve impulse | 7 |
| neurological system process | 13 |
| response to endogenous stimulus | 7 |
| oxidative phosphorylation | 4 |
| monovalent inorganic cation transport | 6 |
| nitrogen compound biosynthetic process | 6 |
| cell-cell signaling | 8 |
| purine ribonucleotide biosynthetic process | 4 |
| ribonucleotide biosynthetic process | 4 |
| purine ribonucleotide metabolic process | 4 |
| ribonucleotide metabolic process | 4 |
| purine nucleotide biosynthetic process | 4 |
| response to organic nitrogen | 3 |
| transmembrane transport | 7 |
| reproductive cellular process | 4 |
| retinol metabolic process | 2 |
| spermatogenesis | 5 |
| male gamete generation | 5 |
| generation of precursor metabolites and energy | 5 |
| purine nucleotide metabolic process | 4 |
| nucleotide biosynthetic process | 4 |
| nucleobase, nucleoside, nucleotide and nucleic acid biosynthetic process | 4 |
| nucleobase, nucleoside and nucleotide biosynthetic process | 4 |
| multicellular organism reproduction | 6 |
| reproductive process in a multicellular organism | 6 |
| ATP biosynthetic process | 3 |
| neuropeptide signaling pathway | 3 |
| cellular respiration | 3 |
| response to extracellular stimulus | 4 |
| purine ribonucleoside triphosphate biosynthetic process | 3 |
| ribonucleoside triphosphate biosynthetic process | 3 |
| purine nucleoside triphosphate biosynthetic process | 3 |
| response to hormone stimulus | 5 |
| positive regulation of transport | 4 |
| germ cell development | 3 |
| nucleoside triphosphate biosynthetic process | 3 |
| response to amino acid stimulus | 2 |
| ATP metabolic process | 3 |
| positive regulation of insulin secretion | 2 |
| response to organic substance | 7 |
| positive regulation of secretion | 3 |
| cation transport | 6 |
| urogenital system development | 3 |
| learning or memory | 3 |
| gonad development | 3 |
| gamete generation | 5 |
| cell morphogenesis involved in differentiation | 4 |
| electron transport chain | 3 |
| purine ribonucleoside triphosphate metabolic process | 3 |
| ribonucleoside triphosphate metabolic process | 3 |
| purine nucleoside triphosphate metabolic process | 3 |
| L-amino acid transport | 2 |
| multicellular organism growth | 2 |
| diterpenoid metabolic process | 2 |
| vitamin A metabolic process | 2 |
| retinoid metabolic process | 2 |
| positive regulation of peptide secretion | 2 |
| ion transport | 7 |
| reproductive developmental process | 4 |
| reproductive structure development | 3 |
| response to acid | 2 |
| development of primary sexual characteristics | 3 |
| gluconeogenesis | 2 |
| terpenoid metabolic process | 2 |
| nucleoside triphosphate metabolic process | 3 |
|  |  |
| **Molecular Function** | |
| structural molecule activity | 8 |
| P-P-bond-hydrolysis-driven transmembrane transporter activity | 4 |
| primary active transmembrane transporter activity | 4 |
| structural constituent of ribosome | 4 |
| ATPase activity, coupled to transmembrane movement of ions | 3 |
| retinol binding | 2 |
| retinal binding | 2 |
| alkali metal ion binding | 4 |
| structural constituent of eye lens | 2 |
| ATPase activity, coupled to transmembrane movement of substances | 3 |
| ATPase activity, coupled to movement of substances | 3 |
| hydrolase activity, acting on acid anhydrides, catalyzing transmembrane movement of substances | 3 |
| retinoid binding | 2 |
| isoprenoid binding | 2 |
| proton-transporting ATPase activity, rotational mechanism | 2 |
| potassium ion binding | 3 |
