# Supplementary material for: Increasing membrane cholesterol of neurons in culture recapitulates Alzheimer’s disease early phenotypes
Source: Mol Neurodegener. 2014 Dec 18;9:60. doi: 10.1186/1750-1326-9-60 (PMC4280040; doi:10.1186/1750-1326-9-60)
Supplement: Supplementary file 5 — Additional file 5: Gene Ontologies for the genes from cluster 4. (DOCX 16 KB) [file 13024_2014_566_MOESM5_ESM.docx]

**Additional file 5.** Gene Ontologies for the genes from cluster 4.

| **GO ID term** | **Number**  **of genes** |
| --- | --- |
| **Cellular component** |  |
| nucleoplasm | 12 |
| cytoplasmic vesicle | 11 |
| cytoplasmic membrane-bounded vesicle | 10 |
| vesicle | 11 |
| membrane-bounded vesicle | 10 |
| nuclear lumen | 13 |
| organelle lumen | 15 |
| membrane-enclosed lumen | 15 |
| melanosome | 4 |
| pigment granule | 4 |
| intracellular organelle lumen | 14 |
| cytosol | 13 |
| nucleoplasm part | 7 |
| cell projection membrane | 3 |
| membrane raft | 4 |
| plasma membrane | 19 |
| cell fraction | 10 |
| basolateral plasma membrane | 4 |
| cell projection | 8 |
| Cajal body | 2 |
| lateral plasma membrane | 2 |
|  |  |
| **Biological Process** |  |
| protein kinase cascade | 8 |
| response to osmotic stress | 4 |
| response to abiotic stimulus | 8 |
| intracellular signaling cascade | 12 |
| response to organic substance | 12 |
| response to endogenous stimulus | 9 |
| actin cytoskeleton organization | 5 |
| actin filament-based process | 5 |
| purine nucleotide catabolic process | 3 |
| response to hormone stimulus | 8 |
| cytoskeleton organization | 6 |
| positive regulation of transport | 5 |
| nucleotide catabolic process | 3 |
| in utero embryonic development | 5 |
| cellular response to hormone stimulus | 4 |
| positive regulation of cell projection organization | 3 |
| regulation of cell cycle | 5 |
| nucleobase, nucleoside, nucleotide and nucleic acid catabolic process | 3 |
| nucleobase, nucleoside and nucleotide catabolic process | 3 |
| intracellular transport | 7 |
| lipopolysaccharide-mediated signaling pathway | 2 |
| response to exogenous dsRNA | 2 |
| nitrogen compound catabolic process | 3 |
| hyperosmotic response | 2 |
| regulation of cell size | 4 |
| heterocycle catabolic process | 3 |
| detection of mechanical stimulus | 2 |
|  |  |
| **Molecular Function** |  |
| metal ion binding | 31 |
| cation binding | 31 |
| ion binding | 31 |
| transition metal ion binding | 22 |
| protein complex binding | 6 |
| insulin receptor binding | 3 |
| zinc ion binding | 17 |
| ATP binding | 15 |
| adenyl ribonucleotide binding | 15 |
| enzyme binding | 8 |
| profilin binding | 2 |
| adenyl nucleotide binding | 15 |
| DNA binding | 15 |
| purine nucleoside binding | 15 |
| nucleoside binding | 15 |
| purine ribonucleotide binding | 16 |
| ribonucleotide binding | 16 |
| protein domain specific binding | 6 |
| mannosyltransferase activity | 2 |
| purine nucleotide binding | 16 |
| oxidoreductase activity, acting on the CH-CH group of donors, NAD or NADP as acceptor | 2 |
| nucleotide binding | 18 |
