# Supplementary material for: Increasing membrane cholesterol of neurons in culture recapitulates Alzheimer’s disease early phenotypes
Source: Mol Neurodegener. 2014 Dec 18;9:60. doi: 10.1186/1750-1326-9-60 (PMC4280040; doi:10.1186/1750-1326-9-60)
Supplement: Supplementary file 6 — Additional file 6: Membrane cholesterol loading causes neuronal EEA1-positive early endosomes enlargement but does not impact on their number, as assessed by confocal microscopy. (A) and (B) Representative confocal images of cortical neurons (DIV 14) stained with an anti-EEA1 antibody in control conditions (A) or after cholesterol loading (B). (C) Mean number of endosomes per soma (ns stands for p > 0.05 in unpaired Student’s t-test). (D) Mean endosomal size (*** stands for p<0.001, Mann-Whitney test). (PPTX 187 KB) [file 13024_2014_566_MOESM6_ESM.pptx]

## Slide 1
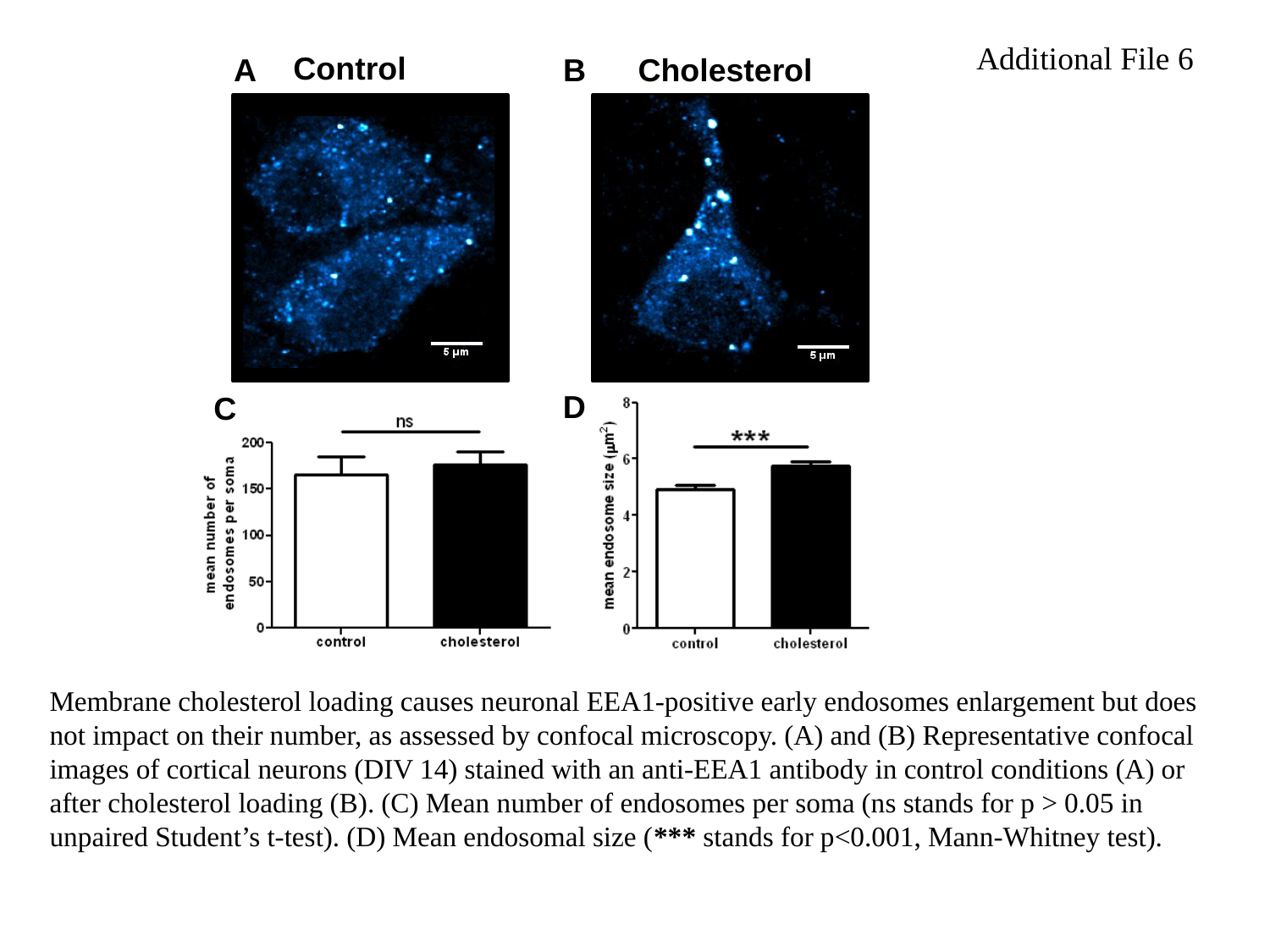

Additional File 6
Control
A
B
Cholesterol
D
C
Membrane cholesterol loading causes neuronal EEA1-positive early endosomes enlargement but does not impact on their number, as assessed by confocal microscopy. (A) and (B) Representative confocal images of cortical neurons (DIV 14) stained with an anti-EEA1 antibody in control conditions (A) or after cholesterol loading (B). (C) Mean number of endosomes per soma (ns stands for p > 0.05 in unpaired Student’s t-test). (D) Mean endosomal size (*** stands for p<0.001, Mann-Whitney test).
